# TGF-β induces GBM mesenchymal transition through upregulation of CLDN4 and nuclear translocation to activate TNF-α/NF-κB signal pathway

**DOI:** 10.1038/s41419-022-04788-8

**Published:** 2022-04-13

**Authors:** Tengfeng Yan, Yinqiu Tan, Gang Deng, Zhiqiang Sun, Baohui Liu, Yixuan Wang, Fanen Yuan, Qian Sun, Ping Hu, Lun Gao, Daofeng Tian, Qianxue Chen

**Affiliations:** Department of Neurosurgery, Renmin Hospital of Wuhan University, Wuhan, Hubei 430060 PR China

**Keywords:** CNS cancer, Epithelial-mesenchymal transition

## Abstract

Glioblastoma (GBM) is the most common and aggressive primary malignant brain tumor. The unregulated expression of Claudin-4 (CLDN4) plays an important role in tumor progression. However, the biological role of CLDN4 in GBM is still unknown. This study aimed to determine whether CLDN4 mediates glioma malignant progression, if so, it would further explore the molecular mechanisms of carcinogenesis. Our results revealed that CLDN4 was significantly upregulated in glioma specimens and cells. The inhibition of CLND4 expression could inhibit mesenchymal transformation, cell invasion, cell migration and tumor growth in vitro and in vivo. Moreover, combined with in vitro analysis, we found that CLDN4 can modulate tumor necrosis factor-α (TNF-α) signal pathway. Meanwhile, we also validated that the transforming growth factor-β (TGF-β) signal pathway can upregulate the expression of CLDN4, and promote the invasion ability of GBM cells. Conversely, TGF-β signal pathway inhibitor ITD-1 can downregulate the expression of CLDN4, and inhibit the invasion ability of GBM cells. Furthermore, we found that TGF-β can promote the nuclear translocation of CLDN4. In summary, our findings indicated that the TGF-β/CLDN4/TNF-α/NF-κB signal axis plays a key role in the biological progression of glioma. Disrupting the function of this signal axis may represent a new treatment strategy for patients with GBM.

## Introduction

Glioma is the most common primary malignant tumor of the central nervous system in adults [1]. Due to the diversity of its sources, it shows extremely high heterogeneity. Among them, glioblastoma (GBM) is the most malignant subtype, with a median survival time of less than one and a half years and a 5-year survival rate of less than 10% [2]. Despite a larger range of surgical resection, postoperative radiotherapy, and combined temozolomide chemotherapy, the prognosis is still unsatisfactory.

Claudin-4 (CLDN4) is an important member of the Claudin family. The Claudins protein is a family of four transmembrane proteins with a molecular weight of 20-27 kDa [3]. There are 27 family members that form tightly connected chains. They have four transmembrane domains and two extracellular structures. The two outer domains of ECL1 and ECL2 bind to the –NH2 and –COOH ends embedded in the plasma membrane to maintain its intercellular integrity and regulate paracellular ion transport [3, 4]. CLDNs are usually located on the cell membrane, and regulate the diffusion of solutes in the intercellular space through homotypic binding [5]. However, CLDN family members that do not form tight junctions are involved in intracellular signal transduction [6]. Overexpression or lack of expression of Claudins protein family members play a key role in the pathological process of diseases such as chronic inflammation and malignant tumors [7, 8]. In gastric cancer, CLDN4 was found to enhance the proliferation, invasion and epithelial-mesenchymal transition (EMT) of gastric cancer cells, and was reversed by miR-596 and miR-3620-3p [9]. Similarly, the overexpression of CLDN4 induced EMT of ovarian cancer cells through PI3K/Akt and the EMT transcription factor Twist1 signal pathway [10]. However, the biological function of CLDN4 is still unclear in GBM.

In this study, CLDN4 is a gene related to the progression of glioma which was determined by The Cancer Genome Atlas (TCGA). Then Kaplan–Meier analysis was performed by TCGA data set, and it was found that CLDN4 was closely related to the overall survival (OS) in glioma, suggesting the predictive value of CLDN4 in the prognosis of glioma patients. In vitro experiments, we explored the biological functions of CLDN4. Based on label-free quantitative proteomics, we confirmed that CLDN4 can regulate the NF-κB signal pathway. Specifically, we discovered CLDN4/TNF-α/NF-κB signal axis in glioma for the first time. In addition, combined with previous studies, we first identified that the classic TGF-β signal pathway can regulate the expression of CLDN4 and nuclear translocation in glioma. Collectively, our study provides a novel mechanism in the progress of GBM, indicating that CLDN4 and its nuclear localization may be a feasible predictive biomarker and potential therapeutic target in patients with GBM.

## Materials and methods

### Human tissue samples

This study was approved by the Ethics Committee of Wuhan University. Human clinical samples were collected from Department of Neurosurgery, Renmin Hospital of Wuhan University, after obtaining consent from the patients. The surgical specimens of patients with traumatic brain injury or tumor specimens were stored in liquid nitrogen for further experiment.

### Cell culture

GBM cell lines (U87, T98, U251, and LN229) and immortalized Normal human astrocyte (NHA) cell line, were provided from The First Affiliated Hospital, Nanjing Medical University. All the cells were cultured in Dulbeccos modified eagle medium (DMEM) supplemented with 10% fetal bovine serum (FBS), and 1% penicillin /streptomycin. All cell lines were maintained at 37°C with 5% CO_2_.

### Cell transfection

Lentivirus contained CLDN4 or CLDN4 shRNA was constructed by Genechem (Shanghai, China) using hU6-MCS-CBh-gcGFP-IRES-puromycin. The sequence of CLDN4 shRNA is: #1, 5ʹ-ccAAGTATTCTGCTGCCCGCT-3ʹ, and #2, 5ʹ-ccACAACATCATCCAAGACTT-3ʹ. The #1 sequence was chosen for the lentivirus package because of its perfect silencing efficiency. 293 T cells were transfected CLDN4 or CLDN4 shRNA by Lipofectamine 3000 reagent (Invitrogen, Carlsbad, CA, USA). The cells were collected and treated after 48 h of transfection. Twenty-four hours later, the medium was changed with fresh DMEM/10% FBS. The supernatants were collected every 24 h for two days and filtered with 0.45 μm nitrocellulose filter. Various supernatants were used to infect glioma cells for 24 h, and then selected with 2 μg/ml puromycin for two weeks. The stable pooled clones were verified by western blot.

### Subcellular protein extraction

The subcellular protein extraction kit (Beyotime, Shanghai, China) was used to extract cytosolic and nuclear proteins. Subcellular fractionation was performed as previously described [11].

### Protein isolation and Western blotting analysis

Total cell protein was extracted using SDS lysis buffer (Beyotime, Shanghai, China) mixed with PMSF (100×). The protein samples were electrophoresed on a 4-20% polyacrylamide gel (Genshare Biological, China), and then transferred to a polyvinylidene fluoride (PVDF) membrane (Merck Millipore, USA). CLDN4, E-cadherin, N-cadherin, Vimentin, p-IKKα/β (Ser176/180), IKKα/β, p-IκBα (Ser32), IκBα, p-NF-κB p65 (Ser536), NF- κB p65 was purchased from Cell Signaling Technology (Danvers, MA, USA). GAPDH and Lamin A/C was purchased from Abcam (UK). The secondary antibodies containing anti-rabbit horseradish peroxidase (HRP) and anti-mouse HRP were obtained from Santa Cruz Biotechnology (Santa Cruz, CA, USA). All antibody details are listed in Supplementary Material, Table S2.

### RNA extraction and qRT-PCR

TRIzol reagent (Takara, Japan) was used to extract total RNA. The SYBR Premix Ex Taq kit (Takara, Japan) was used to quantify the expression of CLDN4 mRNA on the ABI StepOne real-time PCR system (Applied Biosystems, USA). Primers were purchased from Ribobio (Guangzhou, China), and the fold changes in expression were calculated by relative quantification (2^−△△Ct^); U6 RNA was used as an endogenous control. The primer sequences were provided by Ribobio (Guangzhou, China). Primers sequences are provided in Supplementary Table S1.

### Cell proliferation assay

The GBM cells were seeded in 6-well plates. After 4 days, each dish was washed using PBS, fixed with paraformaldehyde (4%) for 30 min and then stained with crystal violet (0.1%). The 5-ethynyl-2-deoxyuridine (EdU) kit (Ribobio, China) was used to estimate proliferation of GBM cells. EdU assay was performed as previously described [12].

### Cell invasion assay

In the invasion assay, Matrigel (Corning) was paved in the transwell chamber (8 μm pore size) at 4 °C overnight. Cells were diluted in serum-free DMEM in the upper chamber with a density of 2.5 × 10^4^ cells per insert. DMEM containing 10% FBS was added into the transwell chamber. The remaining cells in the transwell chamber were removed gently with cotton swabs after 24 h of culture, and the invaded cells on the bottom surface were fixed with 4% paraformaldehyde and then stained with 0.1% crystal violet for 20 min, finally washed for three times. All experiments were performed in triplicate.

### Compounds

To prepare the stock solution, TGF-β1 (Invitrogen Shanghai, China) stock solution was dissolved according to the manufacturer’s instructions. The ITD-1 (Selleck, Shanghai, China) was prepared in 5 mM in DMSO. In vitro studies, ITD-1 (5 mM) stock solution was diluted 1:200 in PBS and added to the culture medium to a final dilution of 1:1000 (5 µM). TNF-α was purchased from Sigma-Aldrich (Shanghai, China), and dissolved according to the manufacturer’s instructions.

### Wound healing assay

Single-cell suspensions were seeded into six-well plates to culture. When the cells reached full confluence, the complete medium was removed and 200 µl of sterile pipette tip head was used to perform a scratch and washed it with PBS. Subsequently, the cells were cultured with serum-free medium and further captured in 0 and 24 h at 37 °C. The percentage of wound closure was calculated as follows: (area of original wound − area of actual wound)/area of original wound × 100.

### In vivo studies

The animal study was approved by the Experimental Animal Ethics Committee of Wuhan University. BALB/c nude mice were purchased from the Animal Laboratory Center of Wuhan University (Wuhan, China). For the xenograft model, U87 cells which carrying luciferase were injected into the lateral ventricle of the mouse. Four-week-old nude mice were purchased from the Shanghai Experimental Animal Center of the Chinese Academy of Sciences. The nude mice were randomly divided into 10 mice per group (total mice, *n* = 30). Then paraffin-embedded tissue sections were determined with haematoxylin–eosin (H&E) and Immunohistochemistry (IHC). Tumor growth was determined by bioluminescence imaging.

### Statistical analysis

The statistical analyses were performed with GraphPad Prism 7. Student’s *t* test was used to compare the two groups. One-way analysis of variance (ANOVA) was used to compare the means difference of multiple groups. Kaplan–Meier analysis (log-rank test) was used to determine nude mice’s overall survival. *P* < 0.05 (*) were considered statistically significant, and the data were presented as the mean ± standard deviation (SD) in all results.

## Results

### CLDN4 is upregulated in glioma cells and tissues

**Fig. 1:**
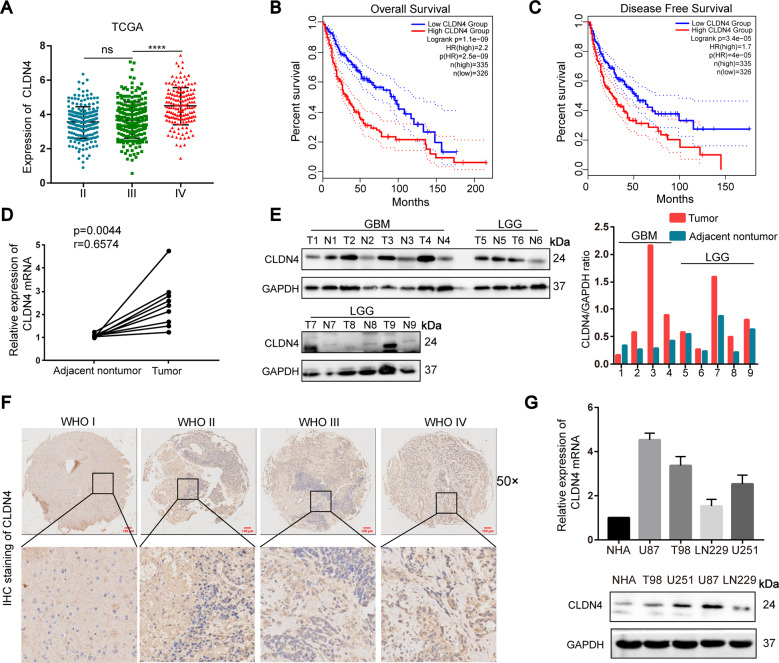
CLDN4 is upregulated in glioma cells and tissues. **A** CLDN4 expression in glioma tissues from the TCGA database (****p < 0.0001, Student’s *t*-test). **B**, **C** The correlation between CLDN4 expression and overall survival (OS) and disease-free survival (DFS) in glioma patients. **D** RT-PCR and Western blotting (N adjacent normal tissue, T tumor tissue) were used to determine CLDN4 protein levels in nine paired GBM samples. **E**, **F** The IHC scores of CLDN4 in patients with different grades of glioma were analyzed, and representative images are shown (50×). **G** The expression of CLDN4 in GBM and NHA cell lines as determined by RT-qPCR and western blotting. (*p < 0.05, **p < 0.01).

Firstly, we found that CLDN4 was highly expressed in high-grade glioma in the Cancer Genome Atlas (TCGA) data set (Fig. 1A). However, compared with normal group, the expression of CLDN4 was significantly increased in glioma group (Fig. S1A). There is no difference in the expression of CLDN4 in the REMBRANT data set (Fig.S1B). In the CGGA data set, CLDN4 was highly expressed in high-grade glioma, compared with WHO grade II glioma (Fig. S1C, D). Kaplan–Meier analysis showed that the expression of CLDN4 was negatively correlated with OS and DFS (*P* < 0.001 for OS and *P* < 0.01 for DFS) (Fig. 1B, C and Fig. S1E, F). In addition, the expression of CLDN4 was higher in GBM tissues than in adjacent normal tissues (Fig. 1D, E). IHC staining was used to detect the expression of CLDN4 in different grades of glioma. We observed that the expression of CLDN4 was highest in GBM patients compared wtih low-grade glioma brain tissues (Fig. 1F). In renal cell carcinoma, when CLDN4 expression was observed in the nucleus, the cancer progressed significantly [13]. This has greatly attracted our attention, but we lack sufficient comprehensive histopathology. Of note, the expression of CLDN4 in the GBM cell lines was significantly increased, compared to the NHA cell line. These results indicated that CLDN4 expression is increased in glioma and negatively correlated with prognosis.

### CLDN4 enhanced the proliferation, migration and invasion capabilities of GBM cells in vitro

**Fig. 2:**
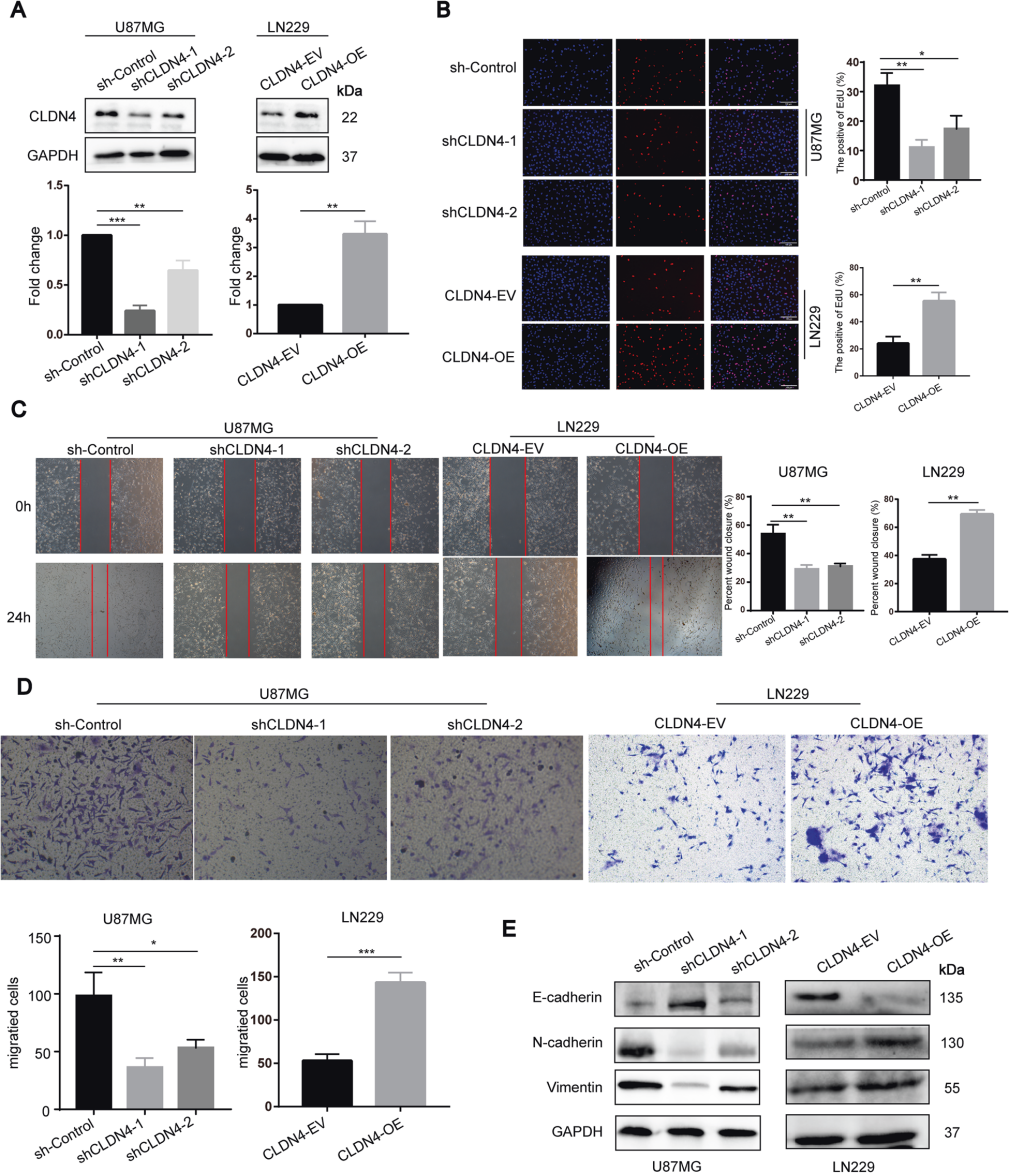
CLDN4 enhances the proliferation, migration and invasion capabilities of GBM cells in vitro. **A** The CLDN4 expression was determined after transfecting cells with corresponding vectors by western blotting in U87 and LN229 cells. **B** The cell proliferation activity was examined by EdU assays. (200×). **C** The migration ability was determined by wound healing. **D** The invasion ability was determined by Transwell assay. **E** The EMT markers were determined by western blotting. Data are represented as the mean ± standard deviation of three independent experiments. Student’s *t*-test, compared to the Control group. *p < 0.05, **p < 0.01.

To further study the effect of CLDN4 on the biological behavior of GBM, silent and ectopic expression models were established in U87 cell and LN229 cell lines in the following studies (Fig. 2A). Compared with the control group, CLDN4 knockdown significantly inhibited cell proliferation, migration and invasion (Fig. 2B–D). In contrast, the overexpression of CLDN4 promoted cell proliferation, migration, and invasion (Fig. 2B–D). Importantly, previous studies have shown that CLDN4 is involved in tumor EMT regulation [10, 14]. To further verify the role of CLDN4 in mesenchymal transition, western blotting analysis was performed in GBM cells. The results showed that overexpressed CLDN4 significantly increased the expression of mesenchymal-related genes in GBM, while epithelial biomarkers were significantly decreased (Fig. 2E). These results indicated that CLDN4 significantly promotes the proliferation, migration and invasion of GBM cells, and that CLDN4 is essential to maintain the mesenchymal properties of GBM.

### CLDN4 gene enrichment analysis

**Fig. 3:**
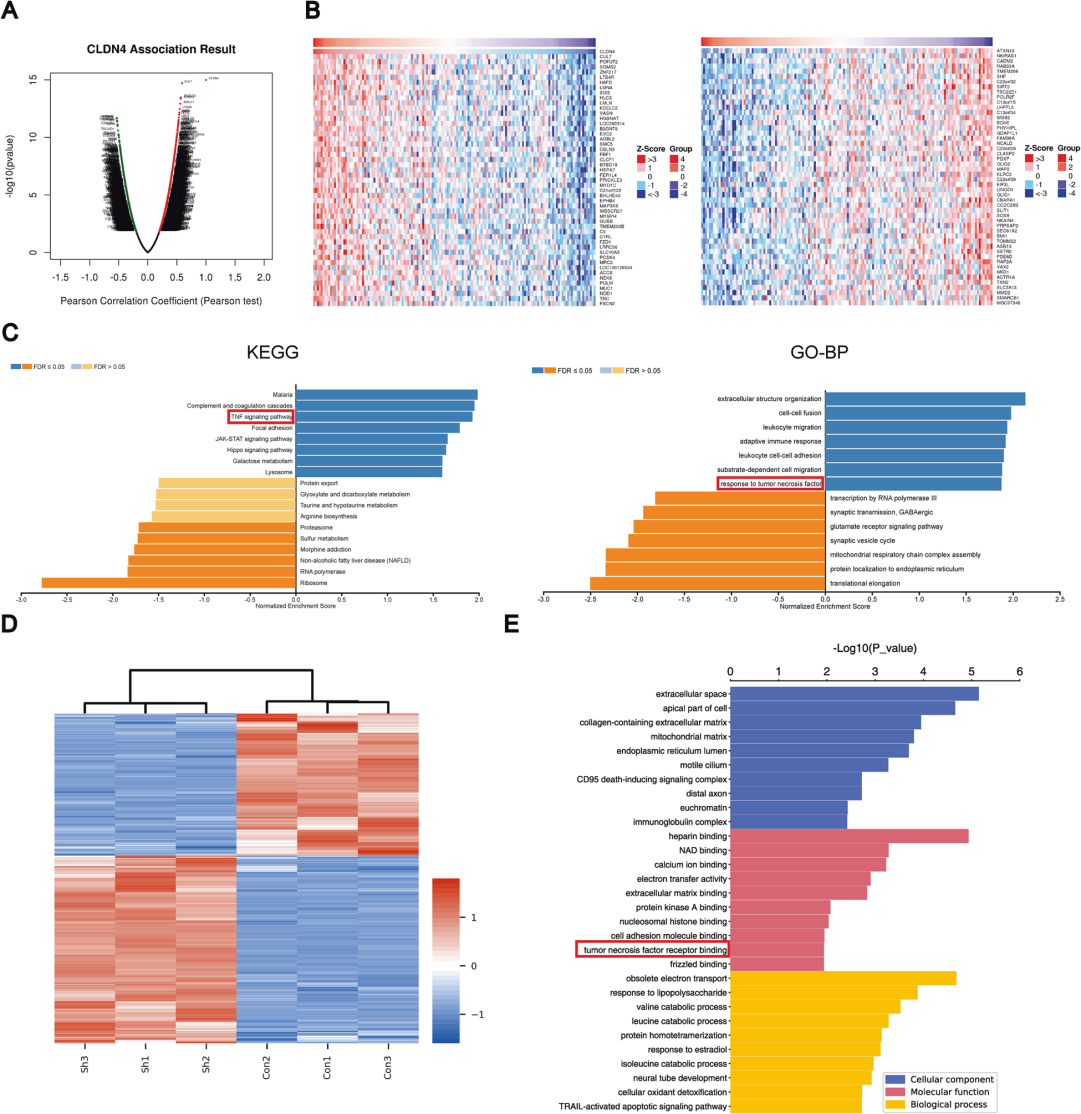
CLDN4 gene enrichment analysis. **A** Volcano plot showing DEGs based on Pearson correlation coefficient in the TCGA data set. **B** Heat map showing relative expression levels (*z*-score) of top 50 most significant DEGs. **C** GO-BP and KEGG pathway analysis of indicated DEGs (FDR < 0.05 and FDR > 0.05). **D** Clustering of DEGs were identified by proteomics. **E** GO analysis performed downregulated genes in proteomics.

The linkeomics website (http://www.linkedomics.org) was used to analyze the sequencing results of 544 GBM patients in the TCGA database. The differentially expressed genes (DEGs) related to CLDN4 expression were represented by volcano plot and heatmap (Fig. 3A, B). KEGG and GO enrichment analysis was performed based on DEGs, the results showed that when CLDN4 expression was downregulated, the expression of TNF-α pathway related genes was significantly downregulated (Fig. 3C). In order to further understand the role of CLDN4 in GBM cells, U87MG-shCLDN4 and U87MG-Con cells were subjected to 4D label-free proteomics analysis (constructed by Shanghai *Genechem* Co., Ltd., China). In the significant difference analysis of quantitative results, we screened the data in the sample group, in which at least half of the repeated experimental data were non-empty values for statistical analysis (fold change >2, *p* < 0.05, regarded as differentially expressed proteins). The results showed that 615 proteins were upregulated, while 467 proteins were downregulated when CLDN4 was knocked down (Supplementary Table S3). Differentially expressed proteins associated with CLDN4 expression were represented by heatmap (Fig. 3D). GO analysis showed that CLDN4 downregulation was mainly related to cell adhesion and TNF-α signal pathway, which was consistent with the bioinformatics analysis results of TCGA database (Fig. 3E). Thus, these molecular pathways may be potentially important signatures of the oncogenic processes of CLDN4 in GBM.

### CLDN4 can regulate NF-κB activity induced by TNF-α

**Fig. 4:**
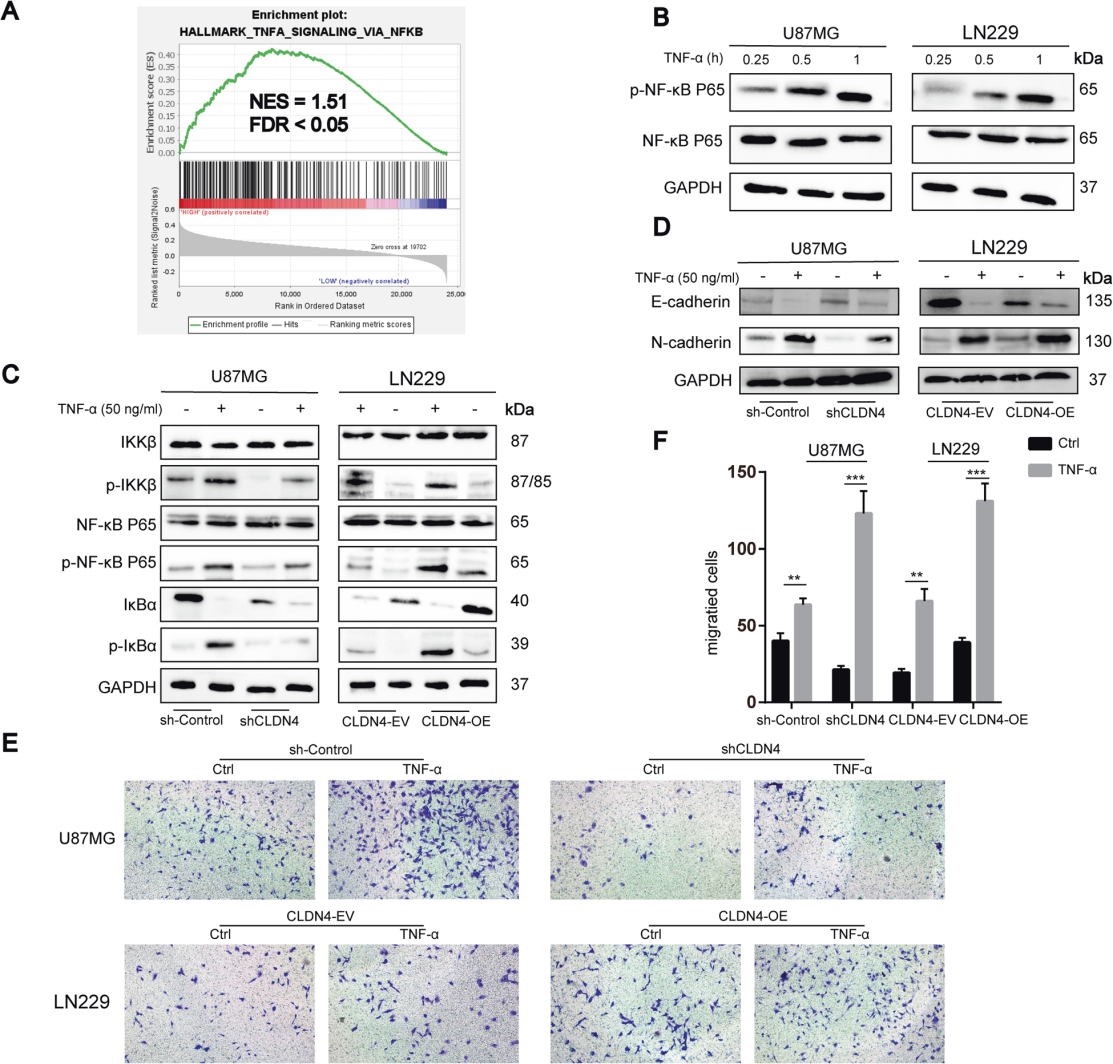
CLDN4 can regulate NF-κB activity induced by TNF-α. **A** According to GSEA, the high expression of CLDN4 in the TNF-α signaling via NF-κB pathway was enhanced from CGGA-693 data set. **B** NF-κB P65 and p-NF-κB P65 expression were determined by western blotting. **C** NF-κB p65, p-NF-κB P65, IκBα, p-IκBα, IKKβ and p-IKKβ were determined by western blotting. **D** Western blotting analysis of EMT markers in GBM cells. **E**, **F** Invasion ability was determined by Transwell assay. Representative images of three cases were shown. Data are represented as the mean ± standard deviation of three independent experiments. Student’s *t*-test, compared to the Control group. **P < 0.01, ***P < 0.001.

We previously used the results of TCGA and protein profiling to determine that CLDN4 may regulate the TNF-α signal pathway (Fig. 3C–E). Based on GSEA, high CLDN4 expression was enriched in the TNF-α signaling by NF-κB pathway in CGGA-693 data set (Fig. 4A). Previous studies have shown that TNF-α regulate the NF-κB signal pathway by activating the IKK complex [15]. To test this hypothesis, the phosphorylation of IκBα, IKKβ and NF-κB p65 were detected by Western blotting after treatment with TNF-α. Then, we found that TNF-α increased NF-κB P65 levels in a time-dependent manner (Fig. 4B). In addition, we investigated whether CLDN4 modulates the NF-κB activity stimulated by TNF-α. Furthermore, CLDN4 knockdown rescued the phosphorylation of IKKβ, IKBα, and NF-κB P65 induced by TNF-α in GBM cells (Fig. 4C). In addition, we also found that TNF-α can induce mesenchymal transition of GBM cells (Fig. 4D). Next, we found that TNF-α can enhance the migration ability of GBM cells (Fig. 4F). Finally, we used the NF-κB luciferase reporter gene to determine the activity of NF-κB. The results showed that CLDN4 knockdown could inhibit the activation of NF-κB signal induced by TNF-α, while overexpression of CLDN4 could amplify the activation effect of TNF-α on NF-κB (Fig. S2). These results indicated knockdown of CLDN4 can inhibit the activation of NF-κB signal pathway in GBM cells.

### TGFβ1 upregulates CLDN4 in GBM cells

**Fig. 5:**
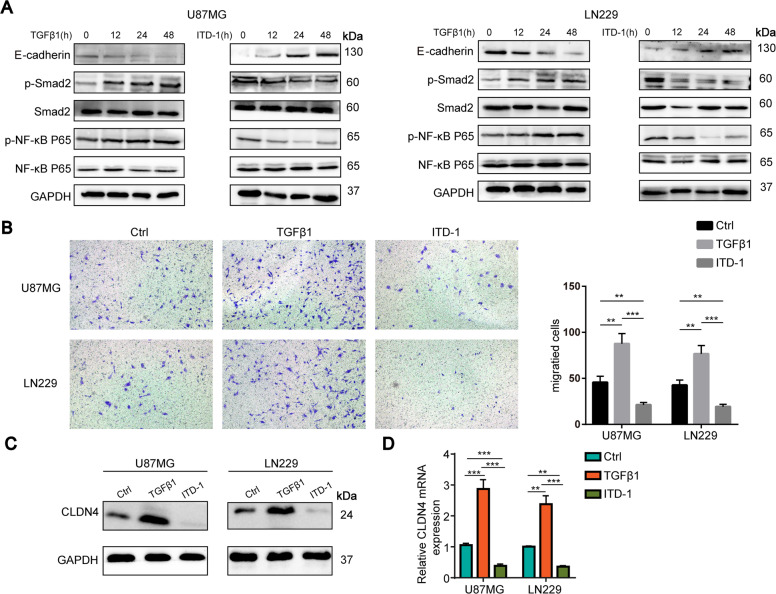
TGFβ1 upregulates CLDN4 in GBM cells. **A** western blotting analysis of E-cadherin, p-Smad2, Smad2, p-NF-κB P65, NF-κB P65 expression in GBM cells after 0 h, 12 h, 24 h, 48 h treatment with TGFβ1 (10 ng·mL−1) or ITD-1 (5uM). **B** The invasion ability of GBM cells was determined by Transwell assay (magnification, ×200). **C** The CLDN4 expression was determined after treated with or without TGFβ1 (10 ng·mL^−1^) or ITD-1 (5uM) by western blotting in GBMcells. **D** The CLDN4 expression was determined after treated with or without TGFβ1 (10 ng·mL^−1^) or ITD-1 (5uM) by RT-qPCR in GBM cells. Representative images of three cases were shown. Data are represented as the mean ± standard deviation of three independent experiments. Student’s *t*-test, compared to the Control group. **P < 0.01, ***P < 0.001.

Further exploration of the upstream signal pathway of CLDN4 is crucial for the treatment of GBM. In the colon, classical TGFβ signal pathway regulates the expression of the connexin CLDN4 [16]. To understand the mechanism of classic TGFβ signal pathway regulating CLDN4 expression in GBM, we conducted a time course experiment in vitro. GBM cells were treated with TGFβ or ITD-1 for 0, 12, 24, and 48 hours. ITD-1 can block the P-Smad2/3 induced by TGFβ2 [17, 18]. The Smad-mediated TGF-β signal is referred to as the classical pathway [19]. Previous studies have shown that TGFβ1 regulate GBM progression and EMT [20]. Next, we found that TGFβ1 promoted Smad2 phosphorylation, EMT-related proteins expression, NF-κB p65 activity and aggravated the invasion of GBM cells (Fig. 5A, B). On the contrary, ITD-1 inhibited the phosphorylation of Smad2 and the expression of EMT-related proteins, NF-κB p65 activity as well as inhibited the invasion of GBM cells (Fig. 5A, B). Furthermore, we found that after treatment of GBM cells with TGFβ for 12 hours, the expression of CLDN4 protein increased, which depended on the duration of TGFβ treatment (Fig. 5C). On the other hand, after GBM cells were treated with ITD-1, CLDN4 protein was significantly reduced. At the same time, in order to clarify whether the change of CLDN4 protein is transcriptional change or translational change, we performed an RT-PCR experiment. We found that the change of CLDN4 mRNA depended on the TGFβ or ITD-1 treatment (Fig. 5D). These results provide valuable clues and reveal that the classical TGFβ signal pathway can participate in the regulation of CLDN4 expression.

### TGFβ1 can promote CLDN4 nuclear accumulation

**Fig. 6:**
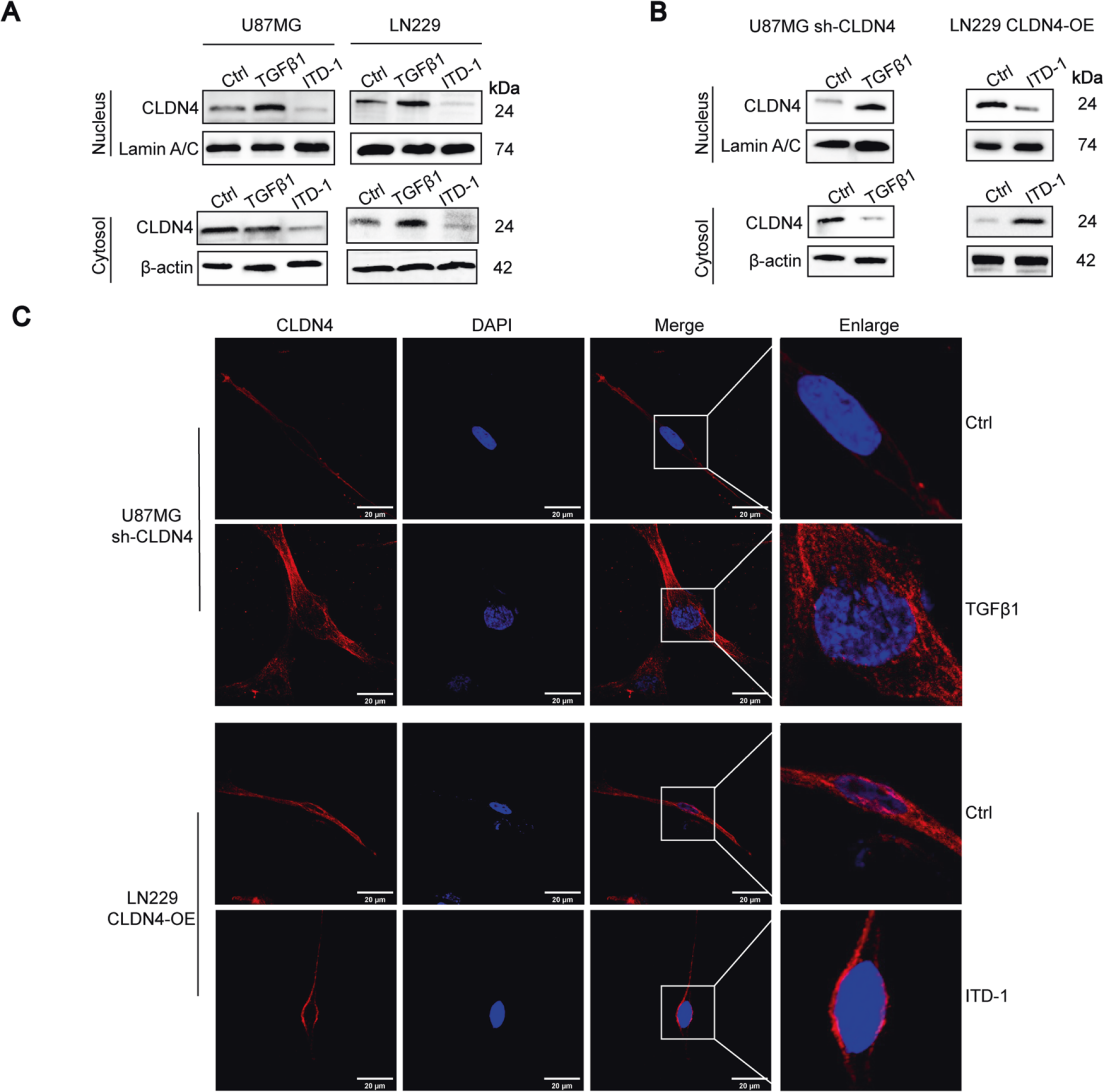
TGFβ1 can promote CLDN4 nuclear accumulation. **A**, **B** The cytosolic and nuclear CLDN4 were determined by western blotting assays. **C** Double immunofluorescence staining of CLDN4 (red) and DAPI (blue) in GBM cells derived from the untreated or treated experimental groups. Representative images of three cases were shown. Scale bar represents 20 μm.

Previous studies have demonstrated that the nuclear accumulation of CLDN4 promotes the malignant progression of kidney cancer and EMT [21]. In addition, PKCε induced the co-nuclear translocation of YAP and CLDN4 in renal cell carcinoma [13]. We found that TGFβ1 promoted the entry of CLDN4 into nucleus, while ITD-1 played the opposite role (Fig. 6A). Meanwhile, by immunofluorescence analysis, TGFβ1 and ITD-1 reversed the expression of cellular nucleus CLDN4 in shCLDN4 and CLDN4-OE, respectively (Fig. 6B, C). In Fig. 6B, ITD-1 promoted the transfer of CLDN4 from nucleus to cytoplasm, and previous studies have shown that ITD-1 can reduce the total protein expression of CLDN4, indicating that nuclear translocation mechanism may be the main way to regulate the expression of CLDN4.

### Knockdown of CLDN4 significantly inhibits tumor growth

**Fig. 7:**
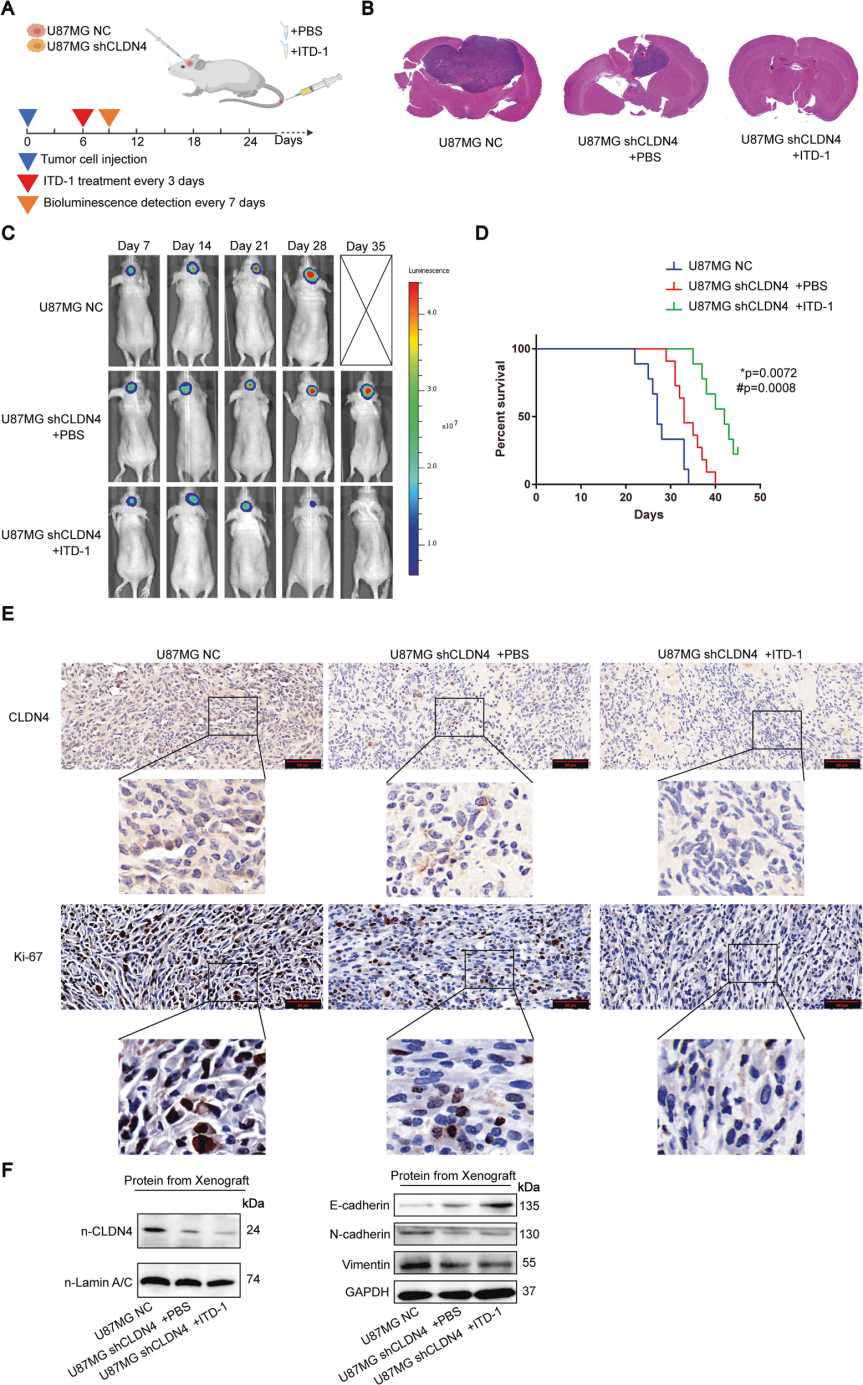
Knockdown of CLDN4 significantly inhibits tumor growth in vivo. **A** Schematic diagram of targeted therapy in vivo. Xenograft models were established by intracranial injection of luciferase-labeled CLDN4 NC or shCLDN4 U87 (1.5 × 10^6^ cells), which were directly inoculated into the lateral ventricle of nude mice. Mice underwent vivo bioluminescence imaging seven days after implantation, and were implemented the same examination in the next every seven days, IVIS imaging was performed to track tumor progression. ITD-1 or PBS was treated six days after xenotransplantation, and treatment was repeated three times a day. **B** H&E staining representative images from brain sections of the untreated or treated experimental groups. **C** Representative cropped bioluminescence images from IVIS imaging showing luminescence of individual mice pictured here over the first 35 days, *n* ≥ 3 mice per cell type. **D** Kaplan–Meier curve representing the percent survival of the untreated or treated experimental groups. **E** IHC representative images of CLDN4 and Ki-67. **F** Western blotting analysis of the xenograft tumors. (*p, comparing the U87MG NC with the U87MG shCLDN4 + PBS group, ^#^*p*, comparing the U87MG shCLDN4 +PBS with U87MG shCLDN4 +ITD-1, Log-Rank test).

In order to further study the anticancer effect of inhibiting CLDN4 expression on GBM progression in vivo, xenograft models were established by intracranial injection of luciferase-labeled CLDN4 NC or shCLDN4 U87 cells, which were directly inoculated into the lateral ventricle of nude mice. In vivo bioluminescence imaging was performed on the 7th day after implantation, and then the same examination was implemented every seven days (Fig. 7A). ITD-1 was treated six days after xenotransplantation, and repeated three times a day. In addition, in order to explore the role of ITD-1, PBS and ITD-1 were added to the shCLDN4 group. The xenograft tumor volume of the shCLDN4 + ITD-1 group was significantly reduced, and the tumor development of GBM was inhibited, compared with CLDN4 NC group and control group (Fig. 7B, C). The overall survival rate of the shCLDN4 + ITD-1 group was also higher than that of the other two groups (Fig. 7D). In addition, proliferating cell-associated antigen Ki67 staining was performed to study the proliferation level of subcutaneous tumors. The Ki67 staining results of the xenograft tumors showed that the Ki67 positive rate in the shCLDN4 + ITD-1 group was significantly reduced, suggesting that the CLDN4 knockdown can inhibit the progression of GBM tumors (Fig. 7E). Finally, we extracted the xenograft protein and found that the shCLDN4 + ITD-1 group significantly inhibited the mesenchymal transformation of GBM cells (Fig. 7F). Therefore, these results prove that the stable downregulation of CLDN4 inhibited the growth and invasion of glioma in xenograft mice, and that combined treatment with ITD-1 can achieve better results.

## Discussion

CLDN4 has been reported to be frequently upregulated in variety of cancers [22, 23]. In this study, we found for the first time that CLDN4 was upregulated in GBM tissues and cells, compared with paired adjacent normal tissues and NHA cell. In addition, patients with high expression of CLDN4 experienced a shorter overall survival time. The results of wound healing and Transwell assays showed that CLDN4 promoted GBM cells migration and invasion. Since EMT contributes to cell motility and invasion, we further examined the EMT markers. Changes in the pool of adhesion molecules expressed and localized on the cell surface are closely related to EMT [24]. For example, the “cadherin switch” is a sign of EMT, accompanied by the downregulation of E-cadherin and the accompanying upregulation of N-cadherin, and is related to the enhancement of tumor invasion and metastasis ability [25, 26]. In kidney cancer, the nuclear accumulation of CLDN4 promotes the malignant progression of tumors and induces EMT [13]. Consistent with previous studies, we also found that CLDN4 regulated the expression of EMT marker proteins.

In order to further explore the function of CLDN4, we conducted bioinformatics prediction and proteomics analysis. Subsequently, we compared proteomic data with TCGA gene sequencing data. We found that both DEGs were involved in the TNF-α signal pathway. There are two identified TNF-α receptors, TNF-α receptor 1 (TNFR1) and TNF-α receptor 2 (TNFR2), both of which belong to the TNFR superfamily [27]. TNF-α is known to activate three signal pathways, namely mitochondrial activated protein kinase (MAPK), nuclear factor kappa B (NF-κB) and apoptosis signal pathway [28]. The NF-κB signal pathway is categorized into “classical” pathway and “non-classical” pathway [29, 30]. The classic NF-κB pathway activation mainly involves the phosphorylation and degradation of IκBα, which promotes the p65 nuclear translocation of NF-κB and the expression of NF-κB-dependent genes [31, 32]. Previous studies have indicated that TNF-α usually activates the typical NF-κB signal pathway in cancers [15, 30]. To explore the mechanism of CLDN4 regulates the TNF-α pathway, we measured IκBα and IKKβ phosphorylation, IκBα degradation and NF-κB p65 activity. Similarly, we also found that CLDN4 knockdown rescued the degradation of IκBα and inhibited the phosphorylation of NF-κB p65, IκBα and IKKβ.

**Fig. 8:**
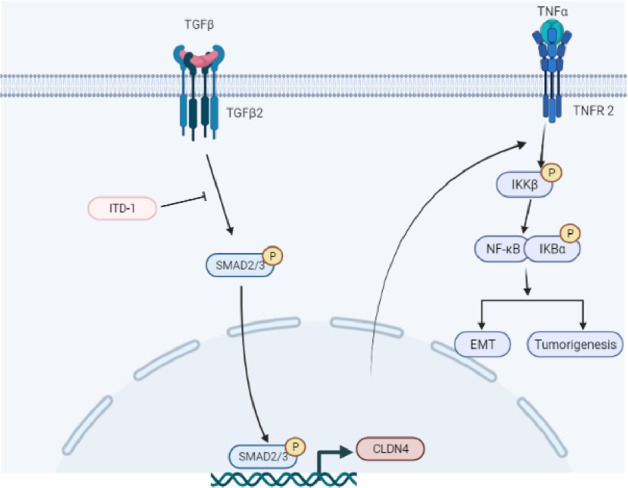
Schematic diagram of the malignant phenotype mechanism of GBM mediated by CLDN4. CLDN4 plays a key role in the progression of GBM tumors. On the one hand, CLDN4 induces mesenchymal transition in GBM cells by regulating the NF-κB signal pathway which is downstream signal pathway of TNF-α. On the other hand, the TGF-β/smad2 signal pathway can regulate the expression of CLDN4 and nuclear translocation.

The TGF-β signaling pathway controls key cellular processes under physiological conditions and regulates many pathological processes including cancer [33–36]. TGF-β plays a variety of biological functions in the body mainly through two ways: the classic SMAD-dependent pathway and the non-SMAD-dependent pathway [37, 38]. Given that TGF-β can disrupt tight junctions, TGF-β and ITD-1 were used for in vitro study. TGF-β signal plays a critical role in tumor EMT [39, 40]. In addition, in this study, we found that TGF-β could promote the expression of CLDN4, and induce mesenchymal transition, as well as NF-κB P65 phosphorylation, while ITD-1 has the opposite result. In triple-negative breast cancer, low expression of CLDN4 was found to be accompanied by high expression of TGF-β [41]. This raises our concerns about why CLDN4 is highly expressed in GBM. Combined with previous studies, CLDN4 has been revealed to be nuclear translocation in tumors [13, 21]. Interestingly, we further found that TGF-β promoted the nuclear translocation of CLDN4. In short, the results presented here provided the first insight into CLDN4, and proposed pro-oncogenic mechanism of CLDN4 (Fig. 8). More importantly, the mechanism of nuclear translocation is still not unclear, it is worthy to further reveal how CLDN4 dynamically regulates the early to late stages of cancer.

## Supplementary information

Western blotting

Supplemental Table3

Supplementary FIGURE LEGENDS

Checklist

Figure S1

Figure S2

Supplemental Table

## Data Availability

The data that supports the findings of this study are available from the corresponding author upon reasonable request.
