# Supplementary figures and images for: TGF-β induces GBM mesenchymal transition through upregulation of CLDN4 and nuclear translocation to activate TNF-α/NF-κB signal pathway
Source: Cell Death Dis. 2022 Apr 13;13(4):339. doi: 10.1038/s41419-022-04788-8 (PMC9008023; doi:10.1038/s41419-022-04788-8)

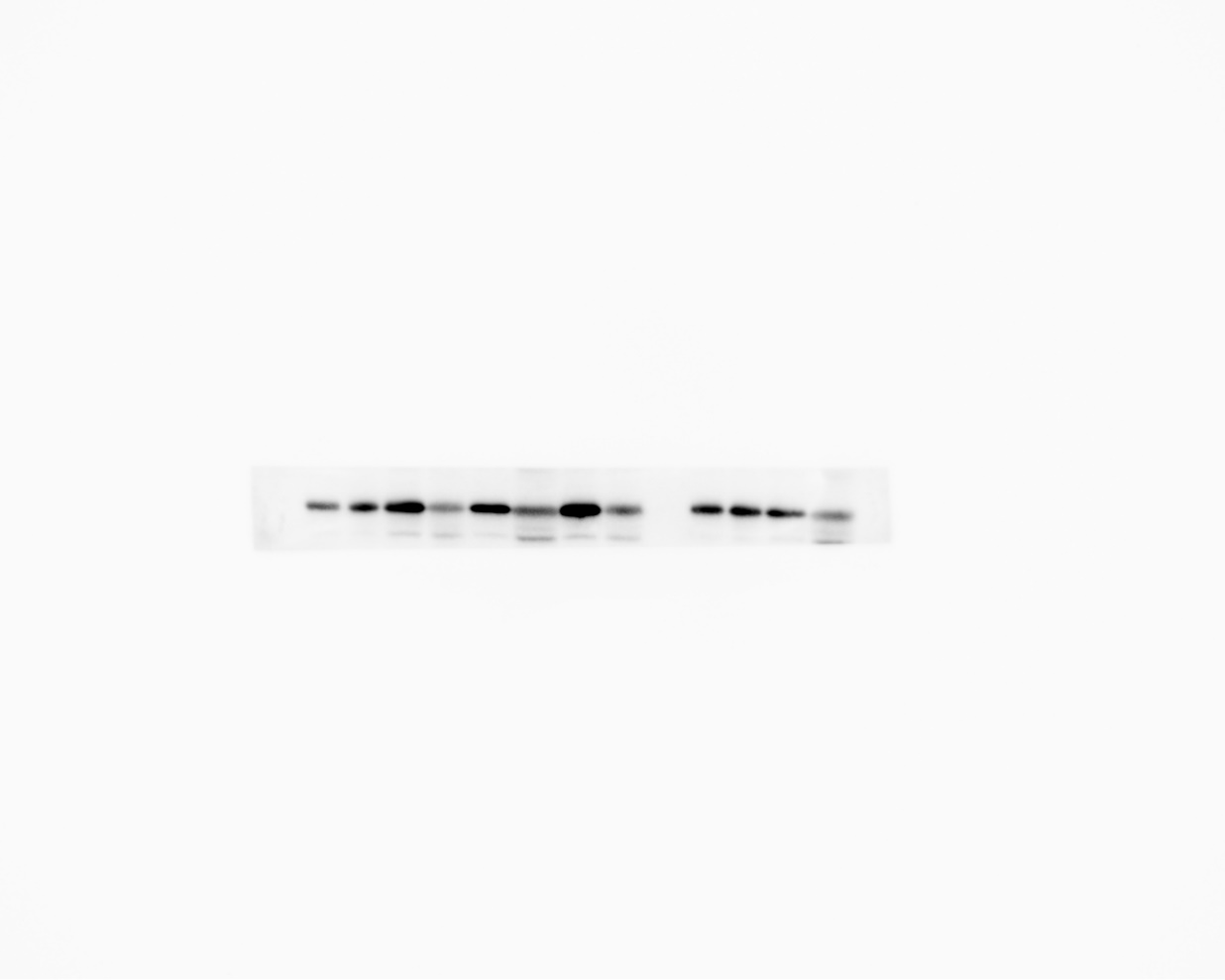

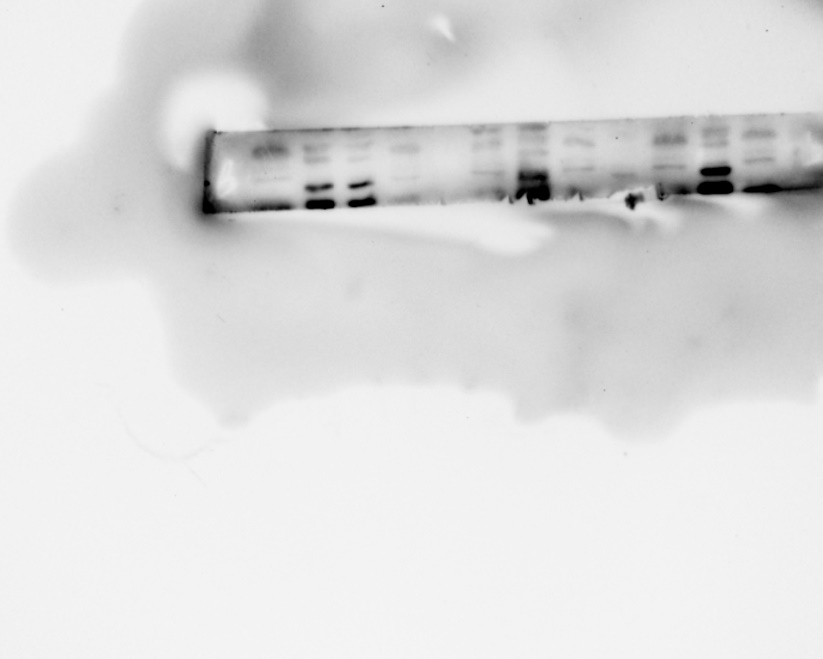

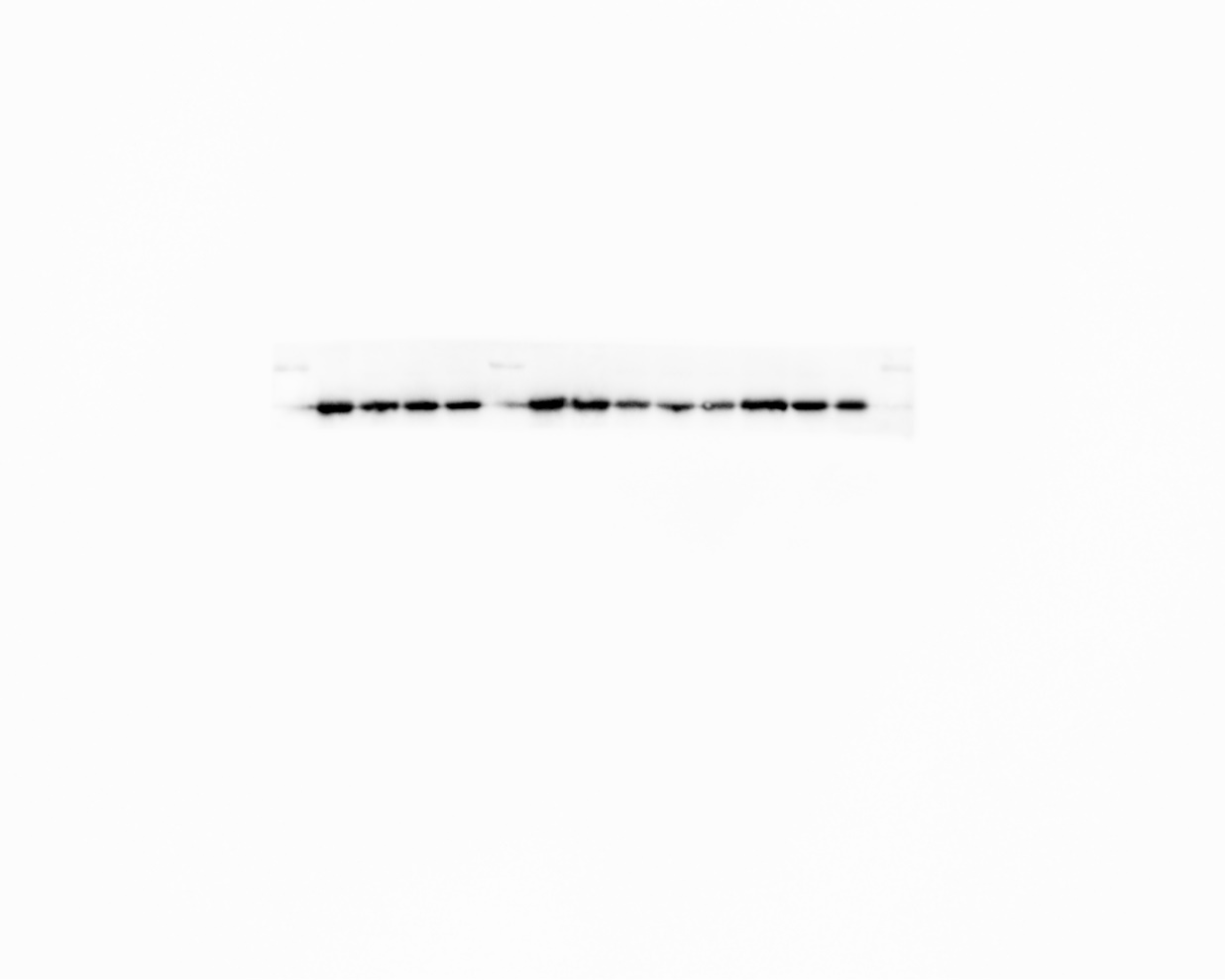

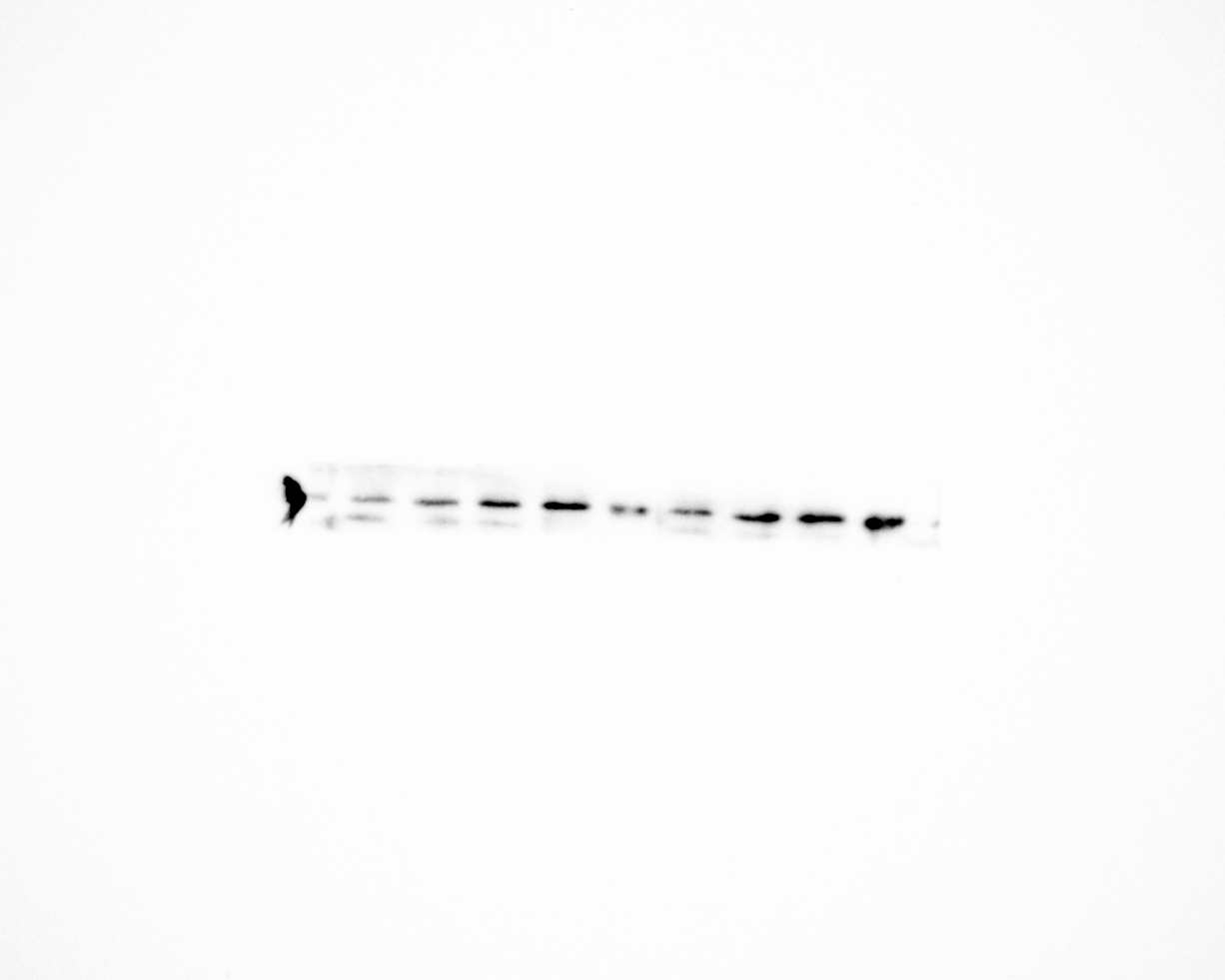

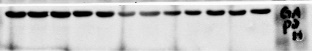

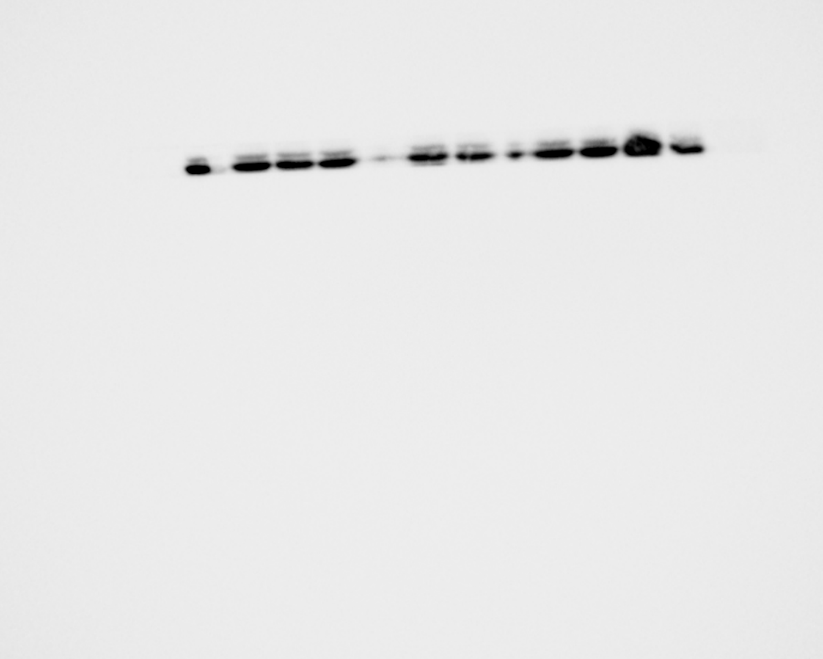

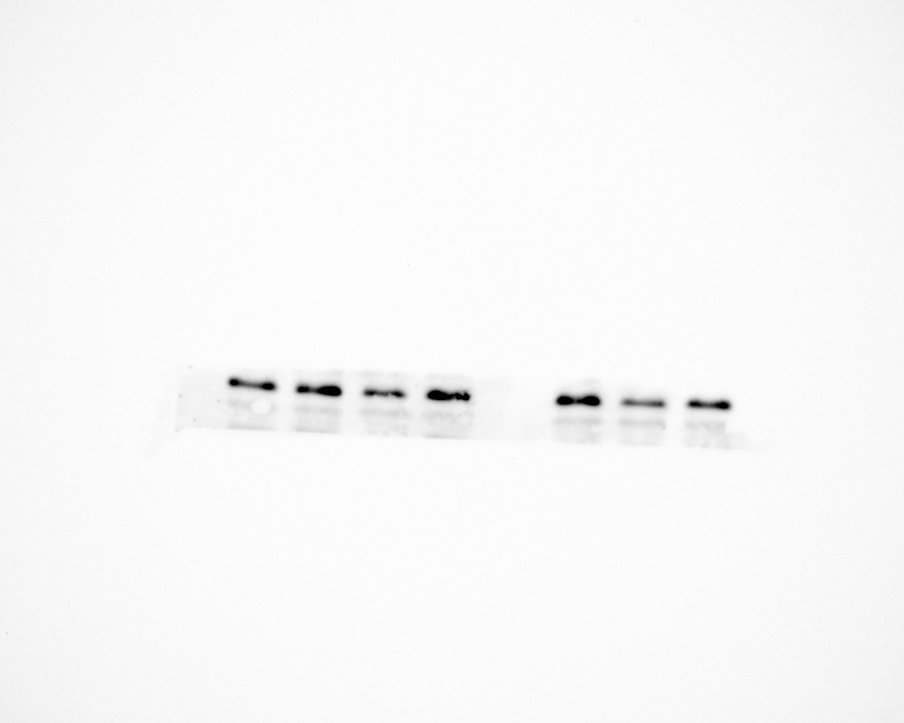

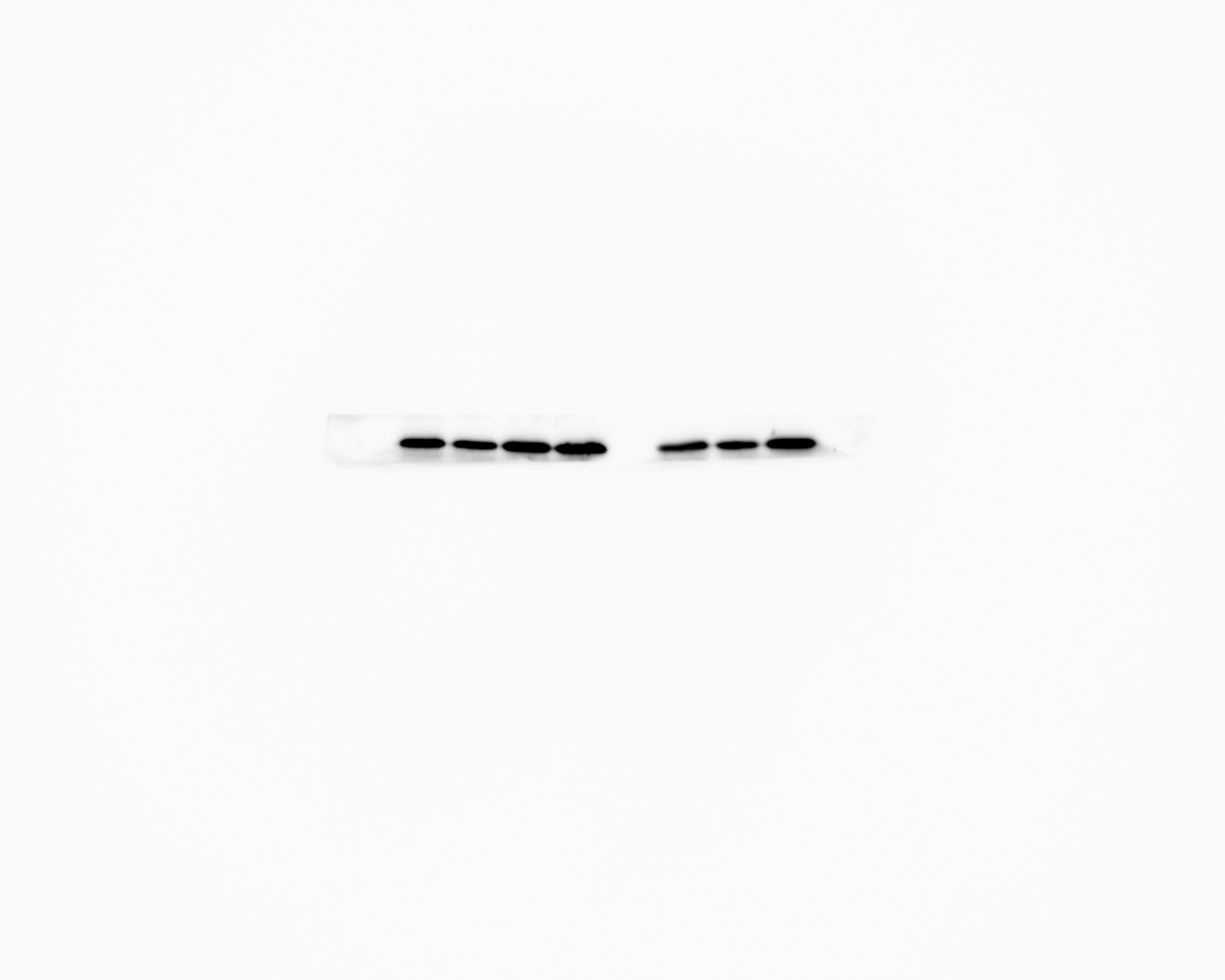

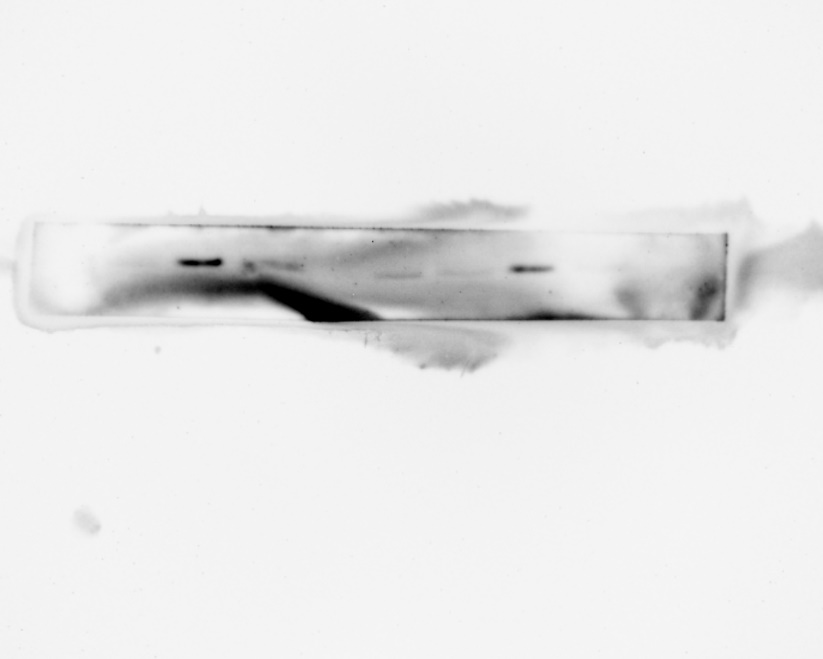

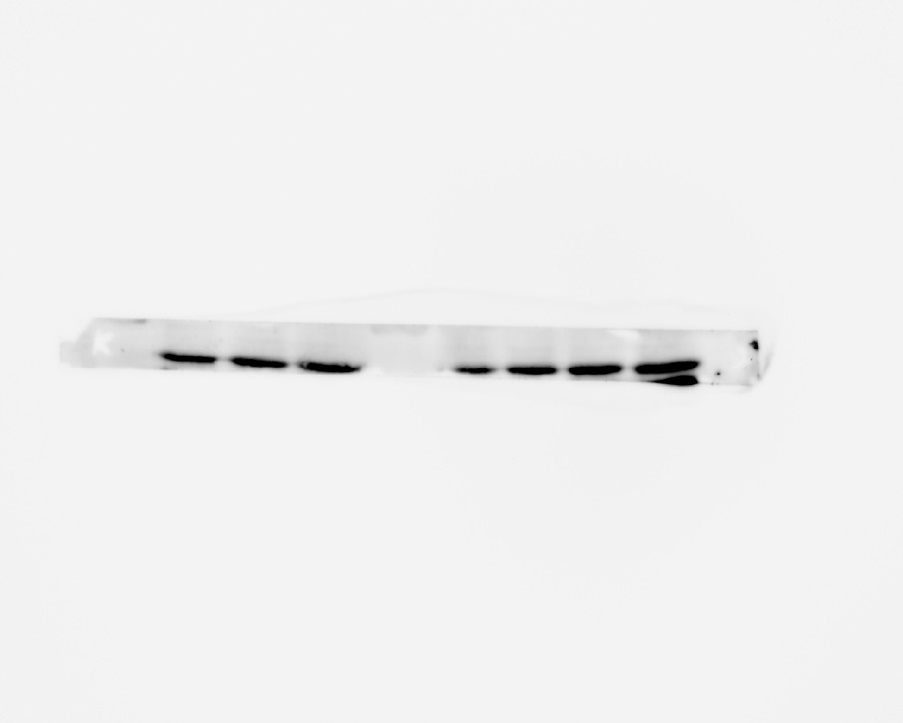

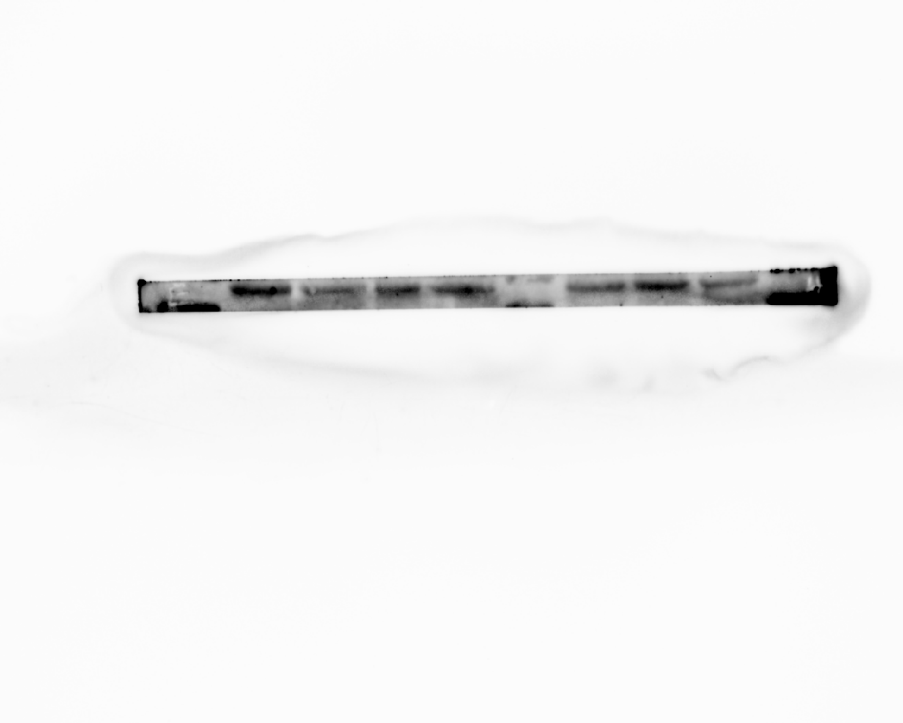

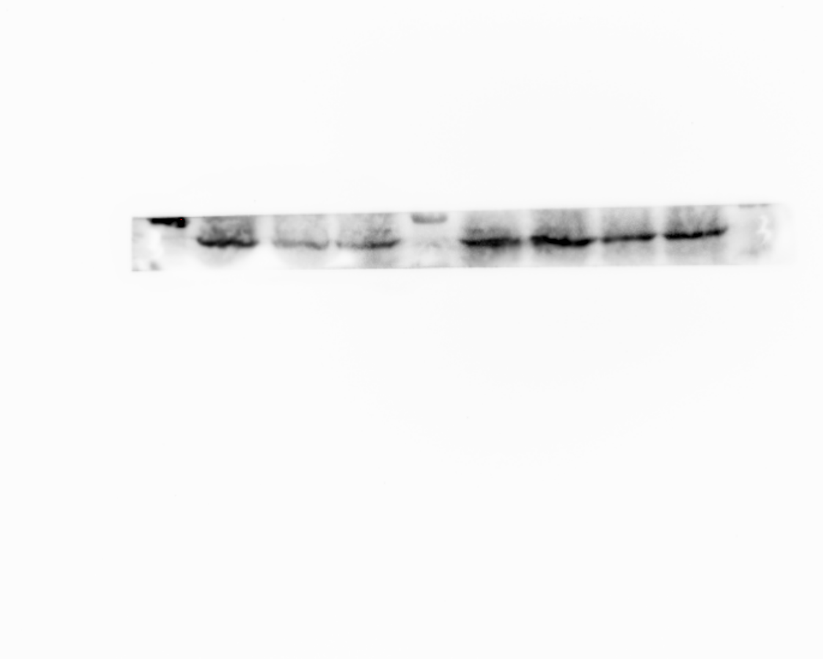

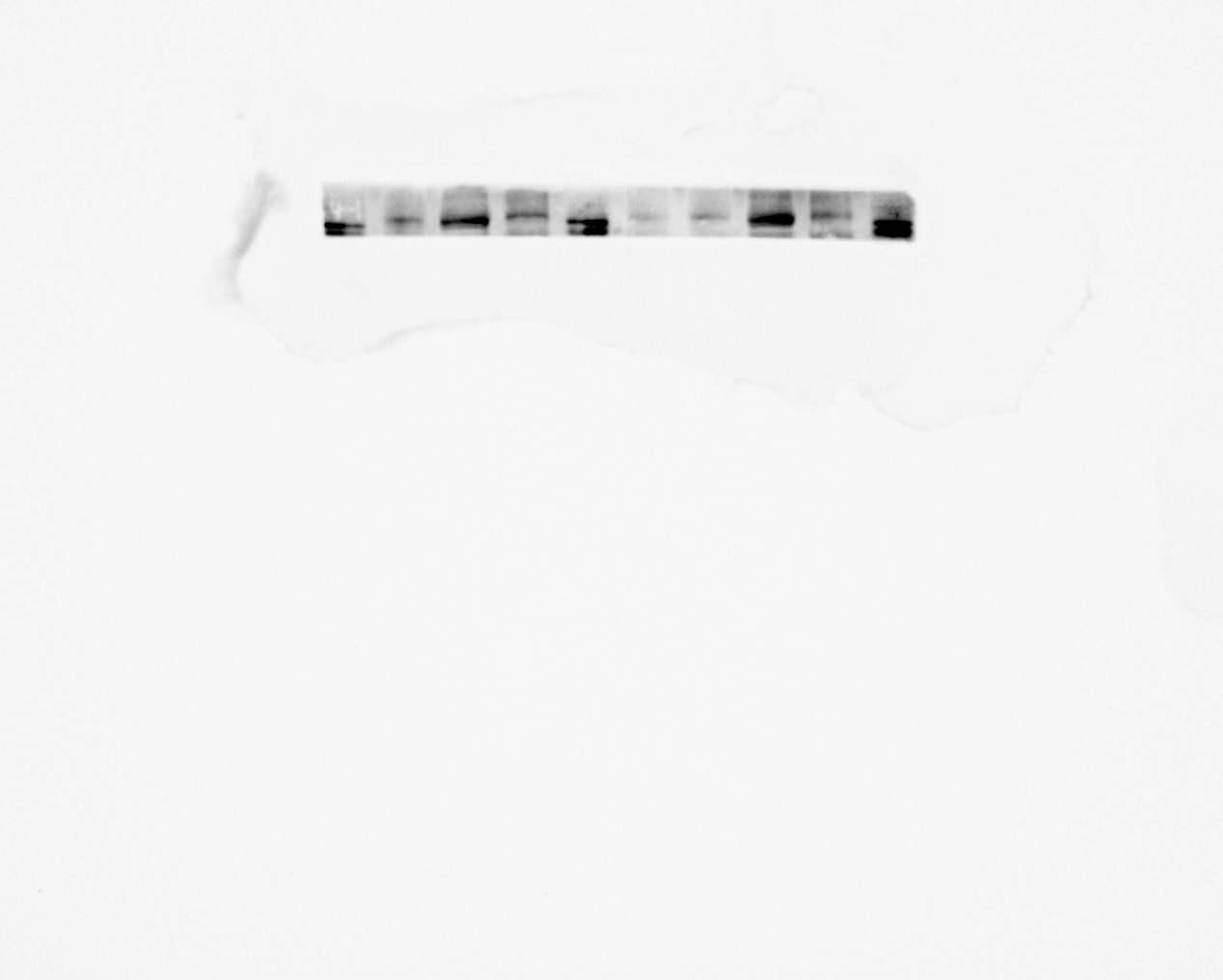

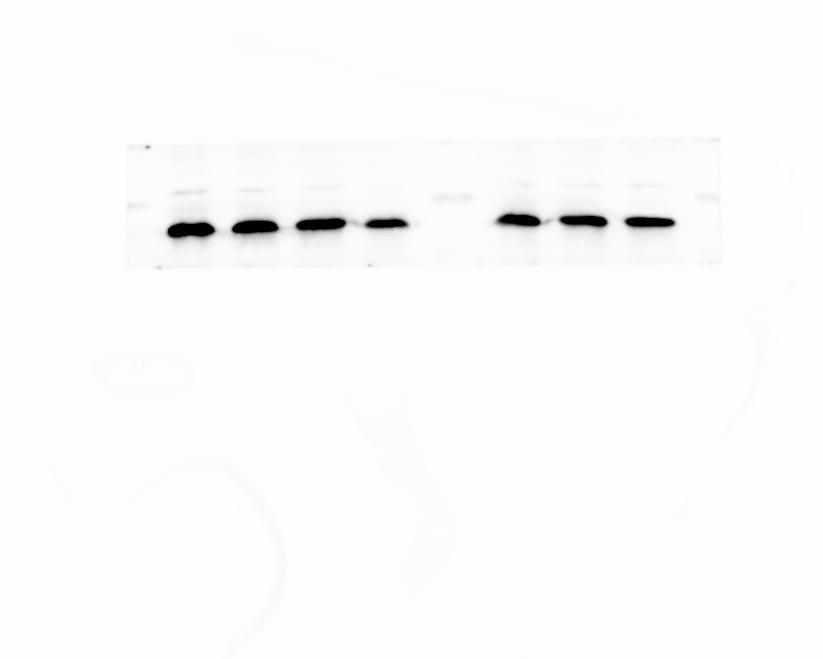

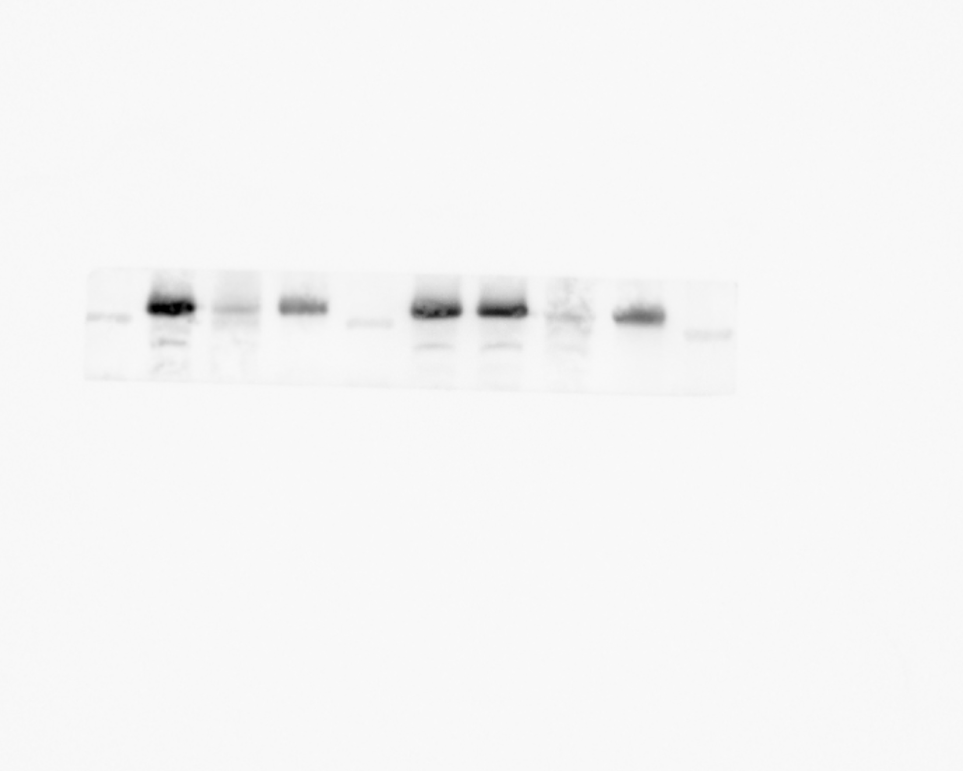

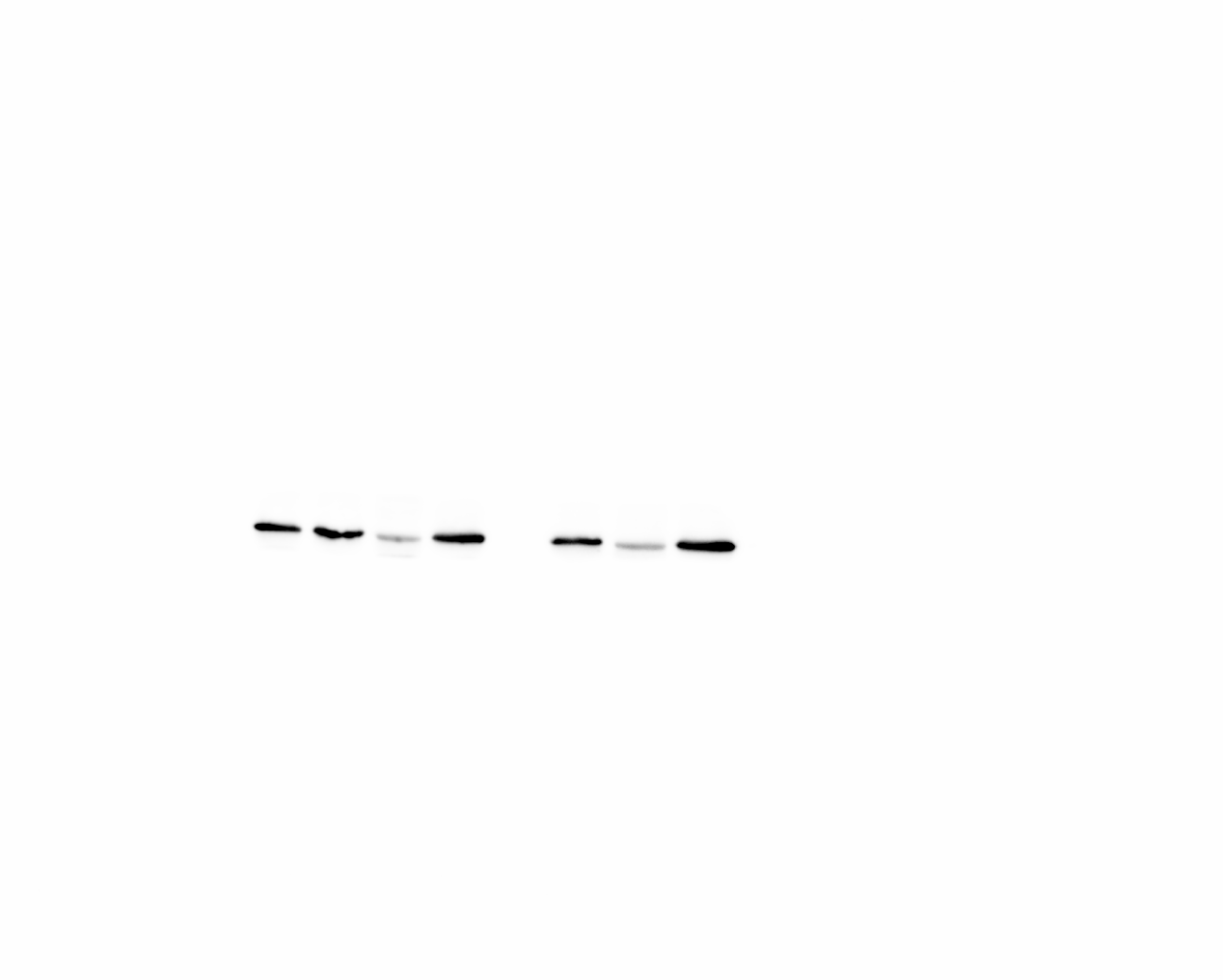

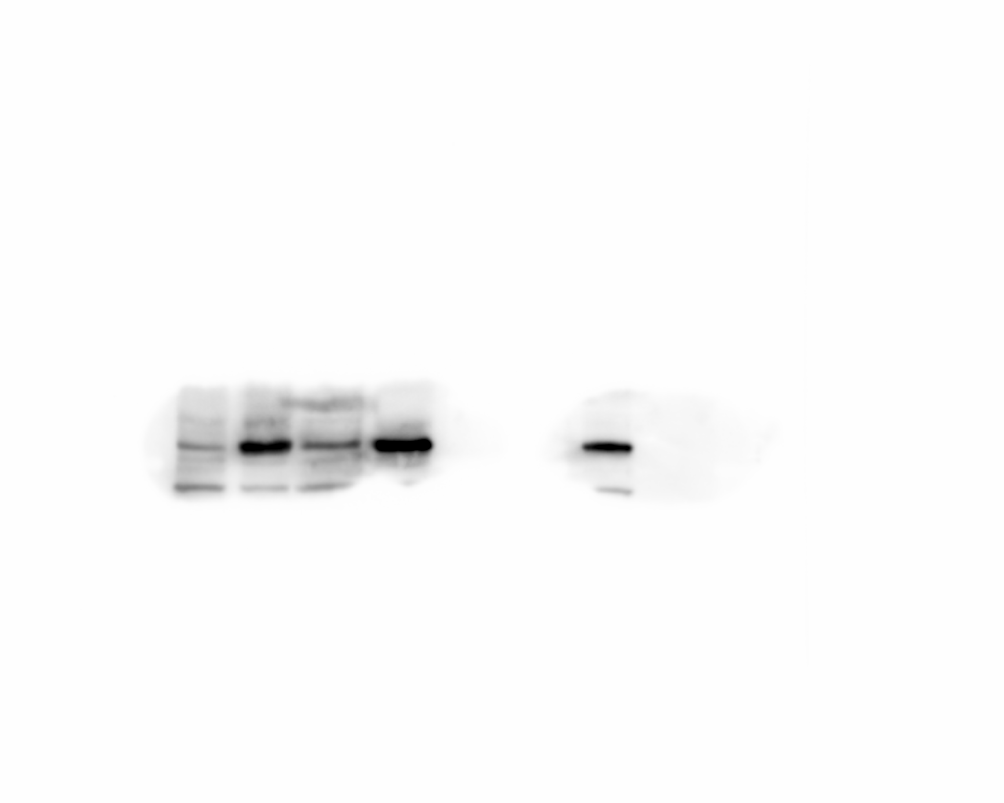

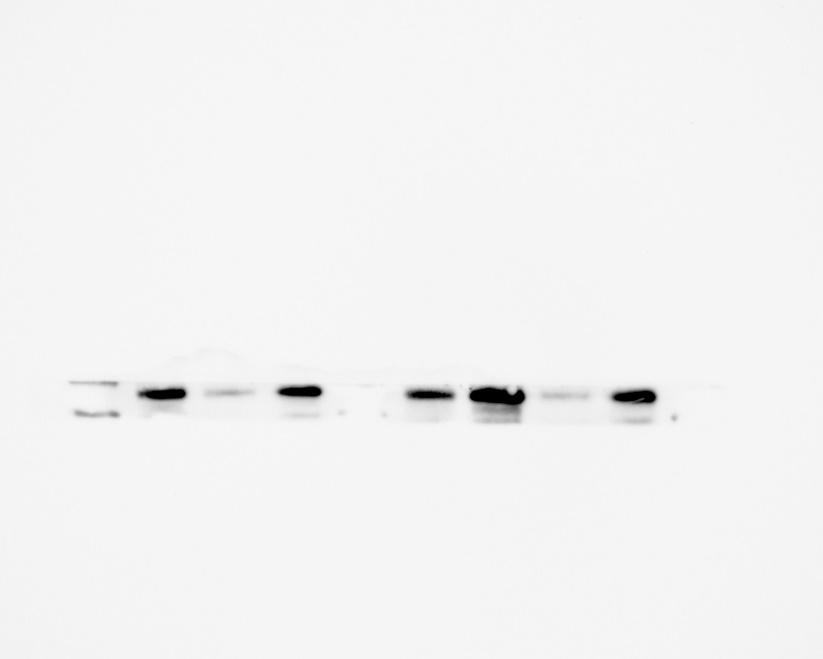

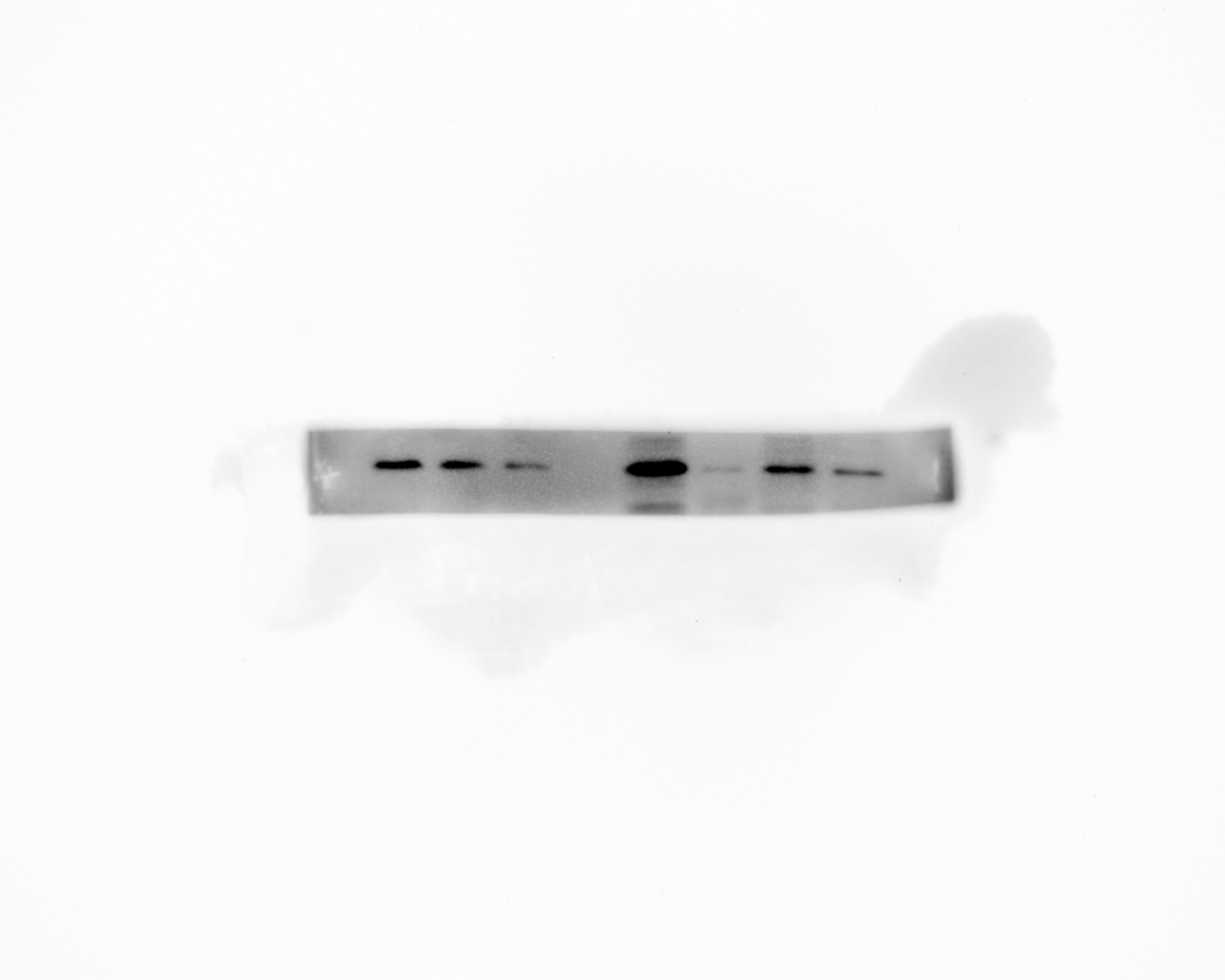

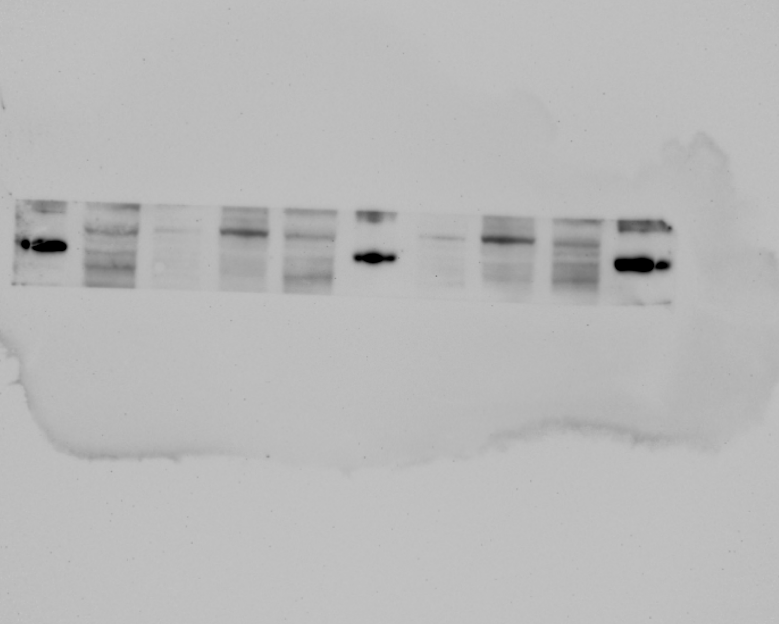

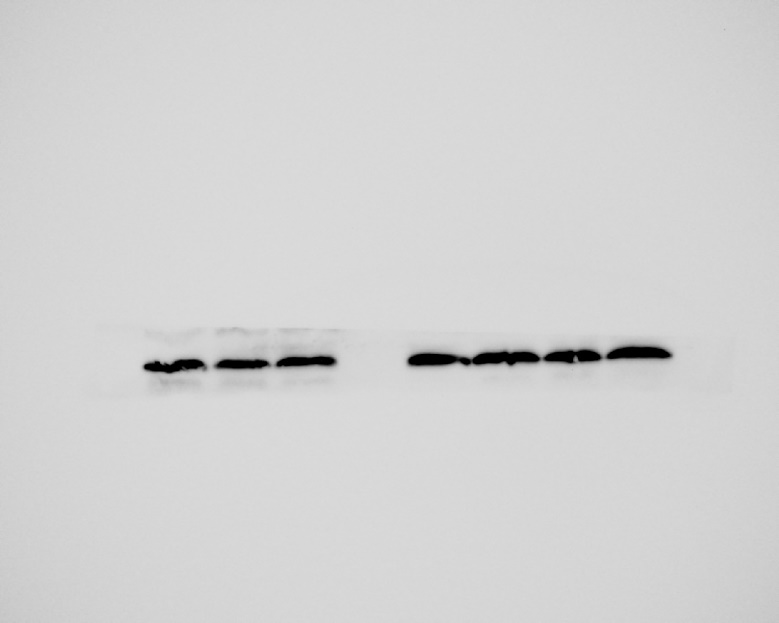

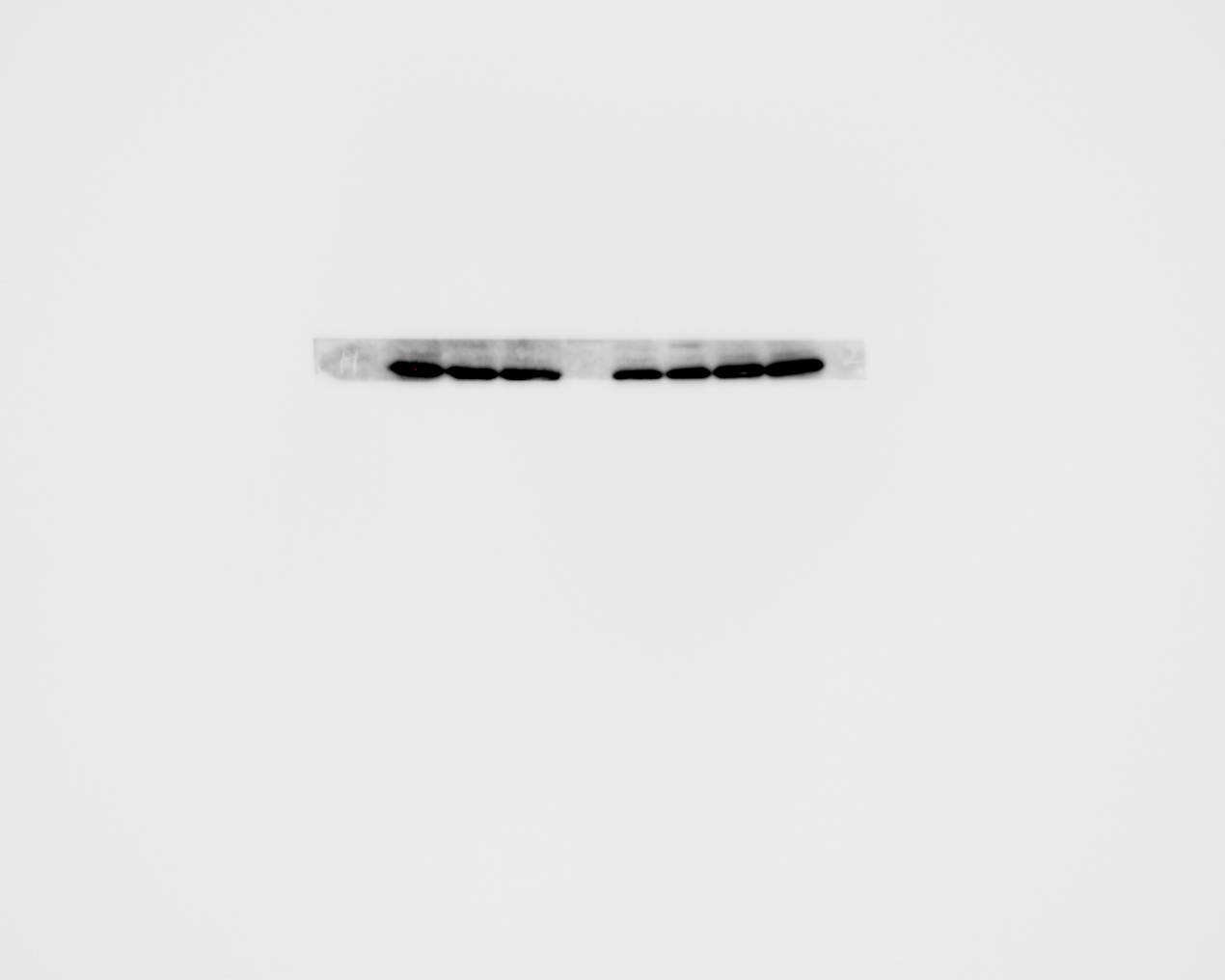

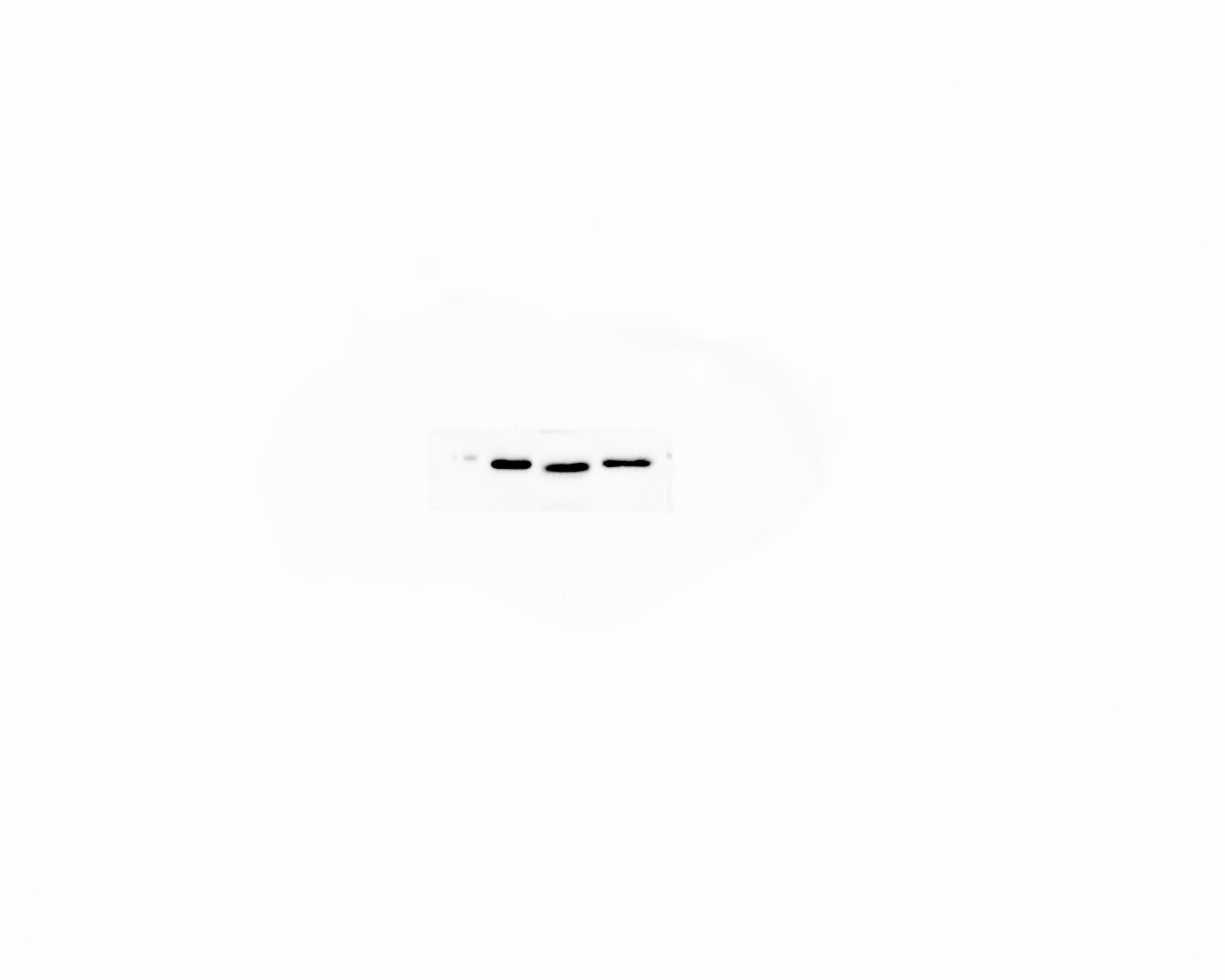

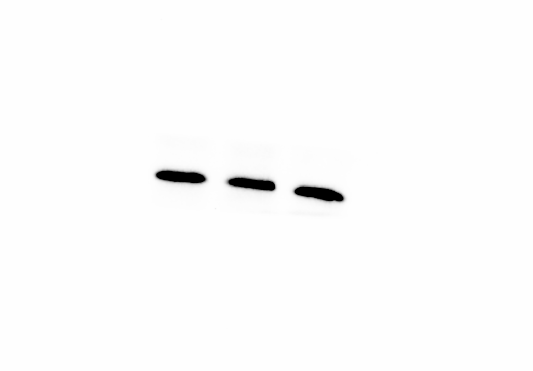

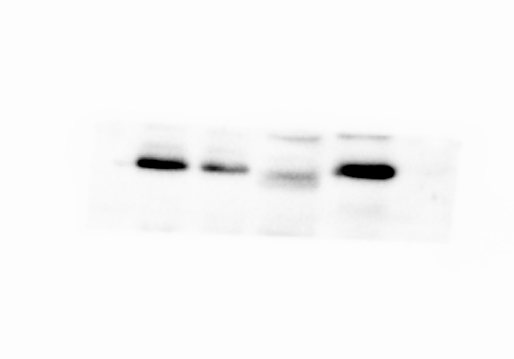

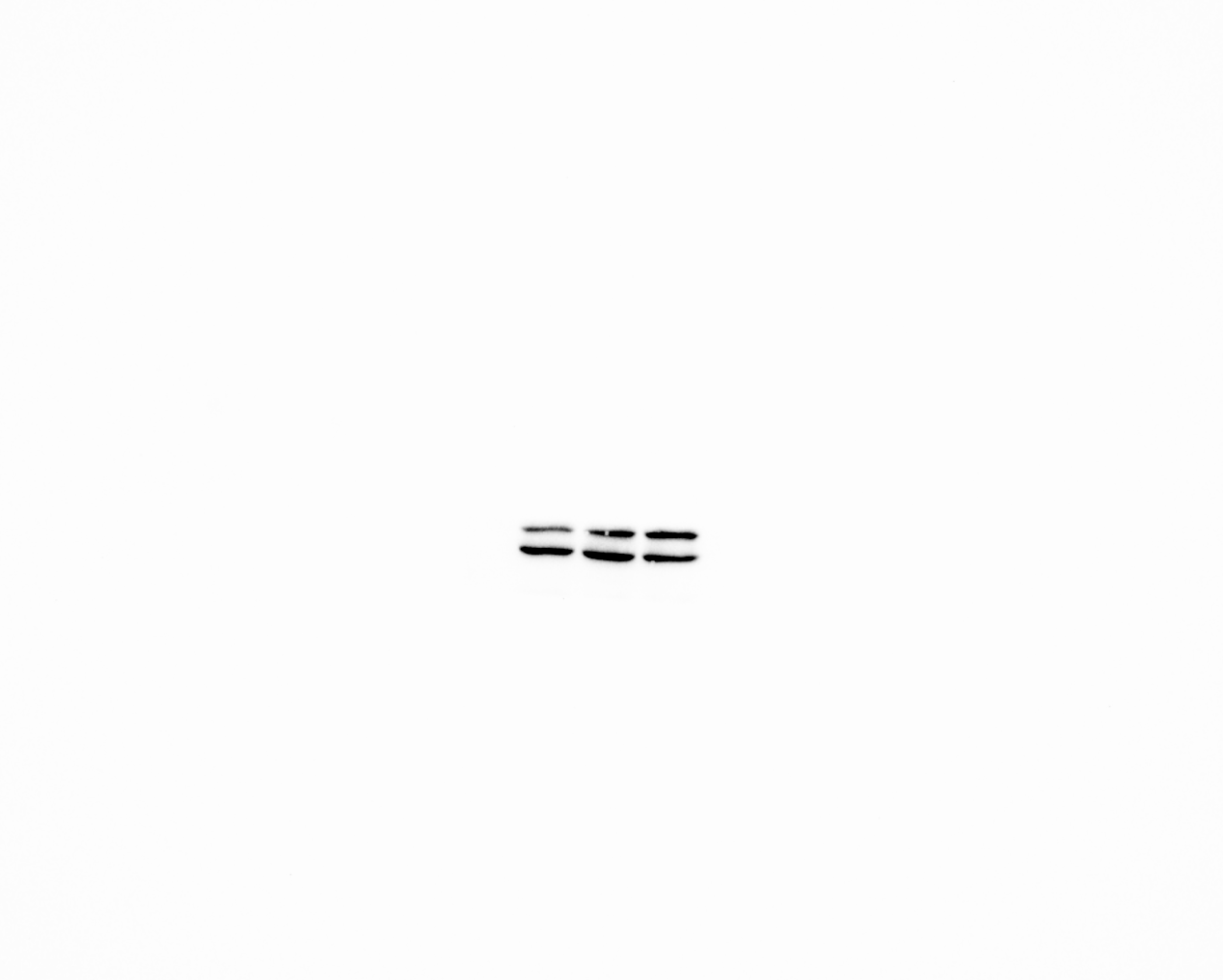

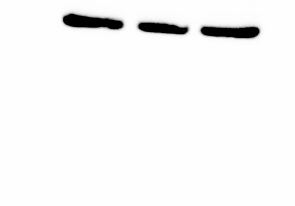

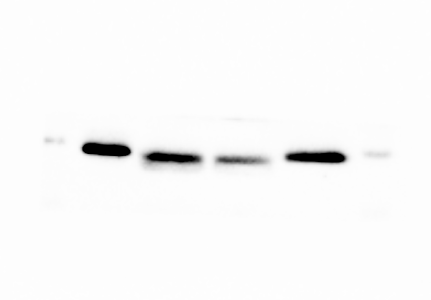

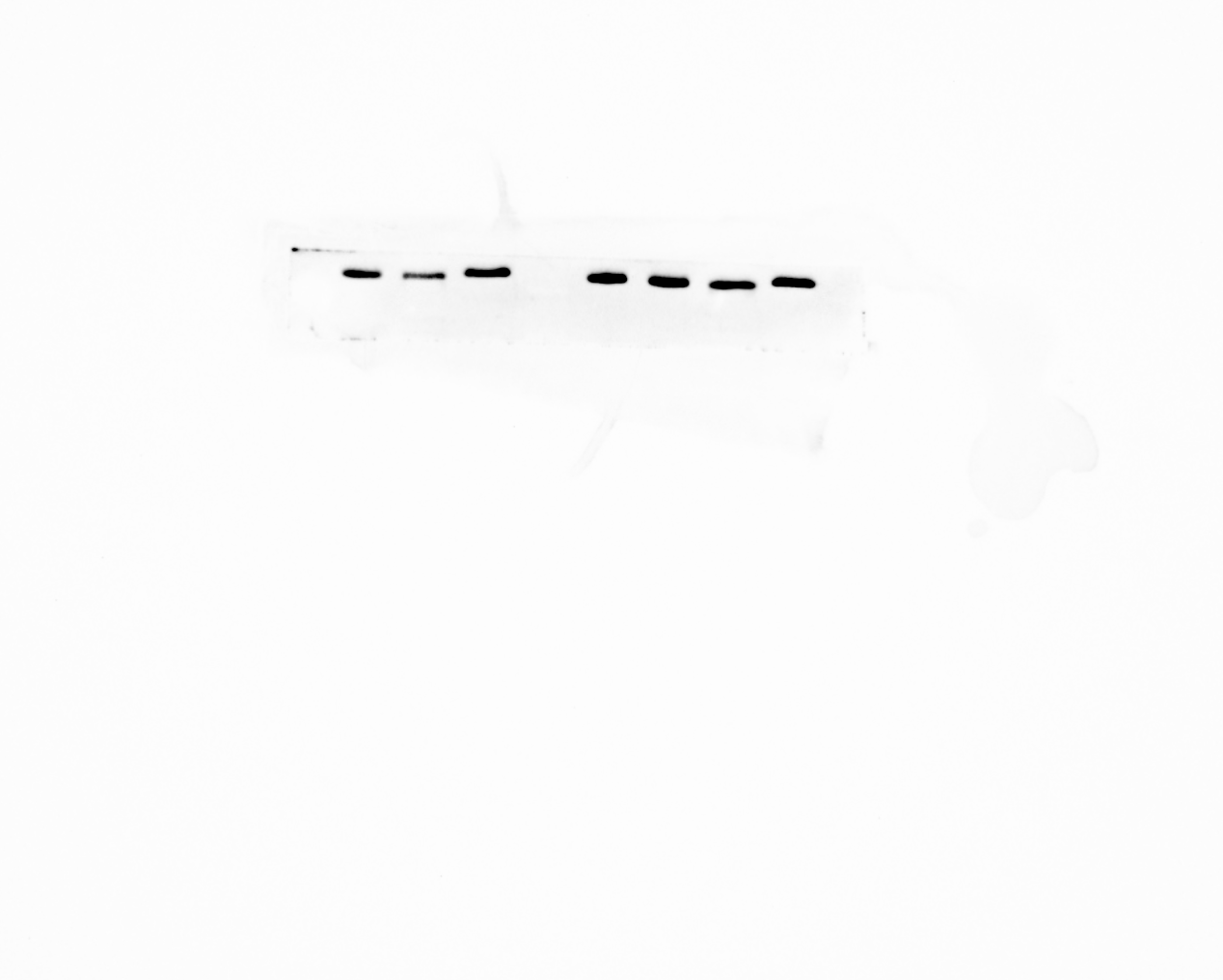

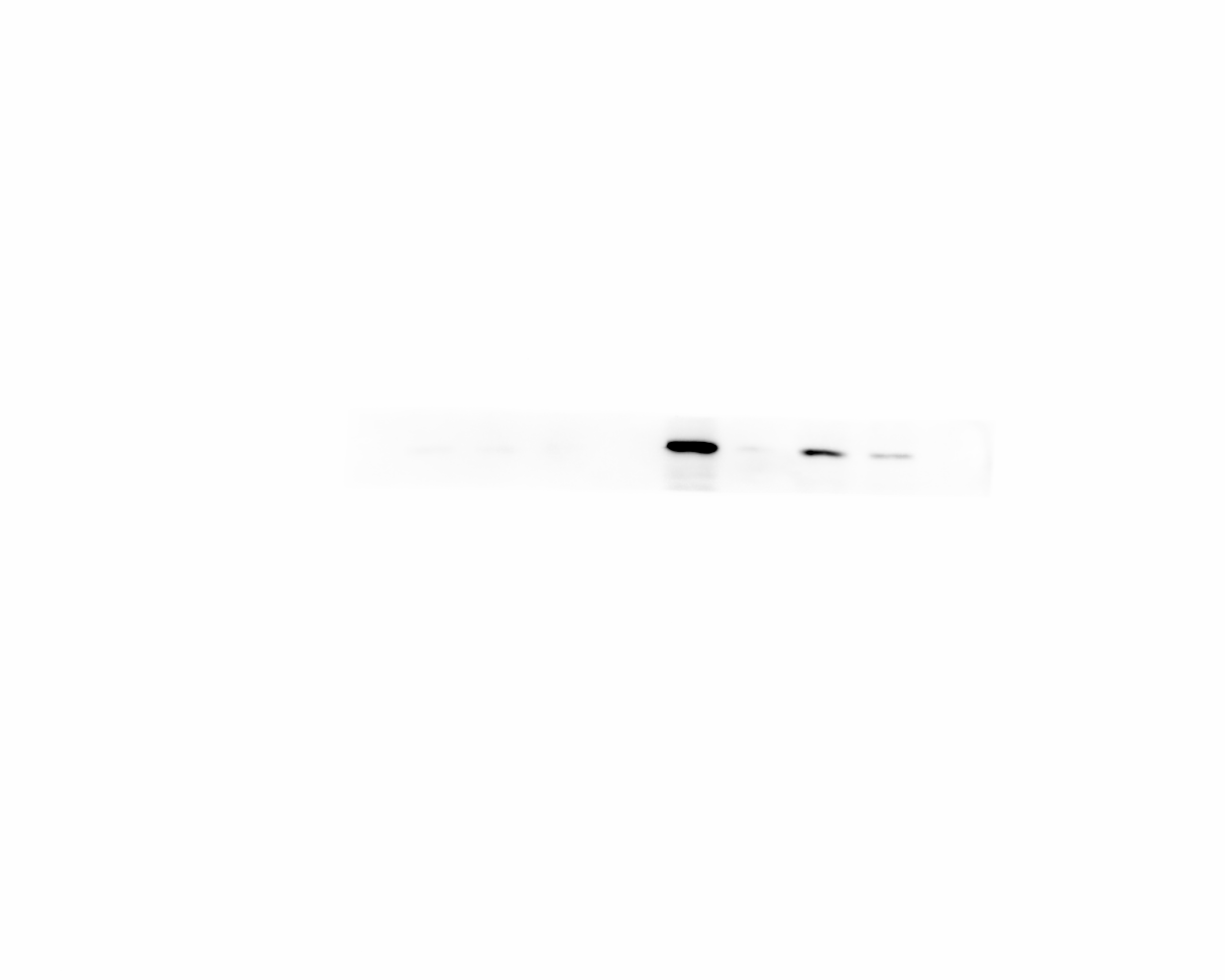

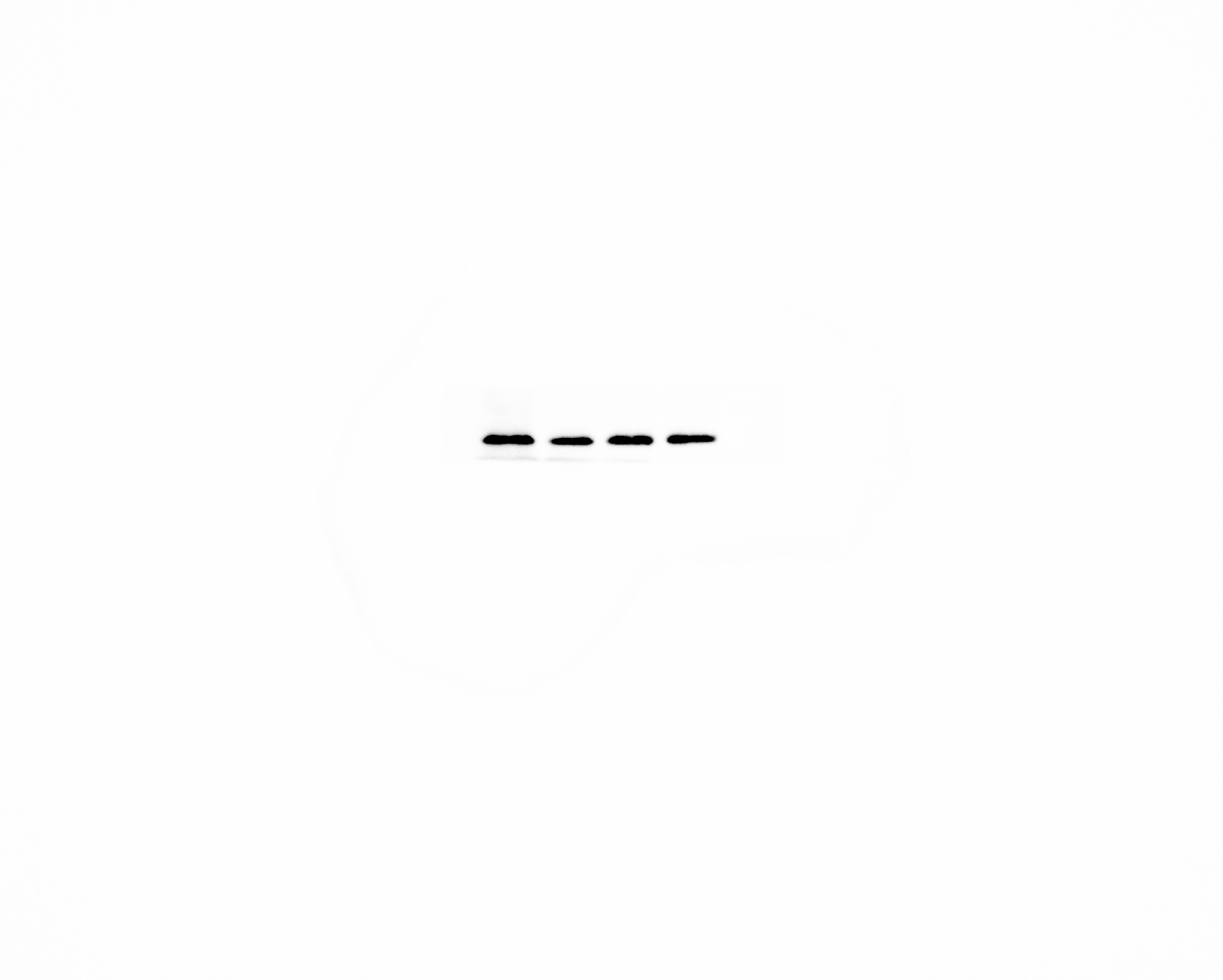

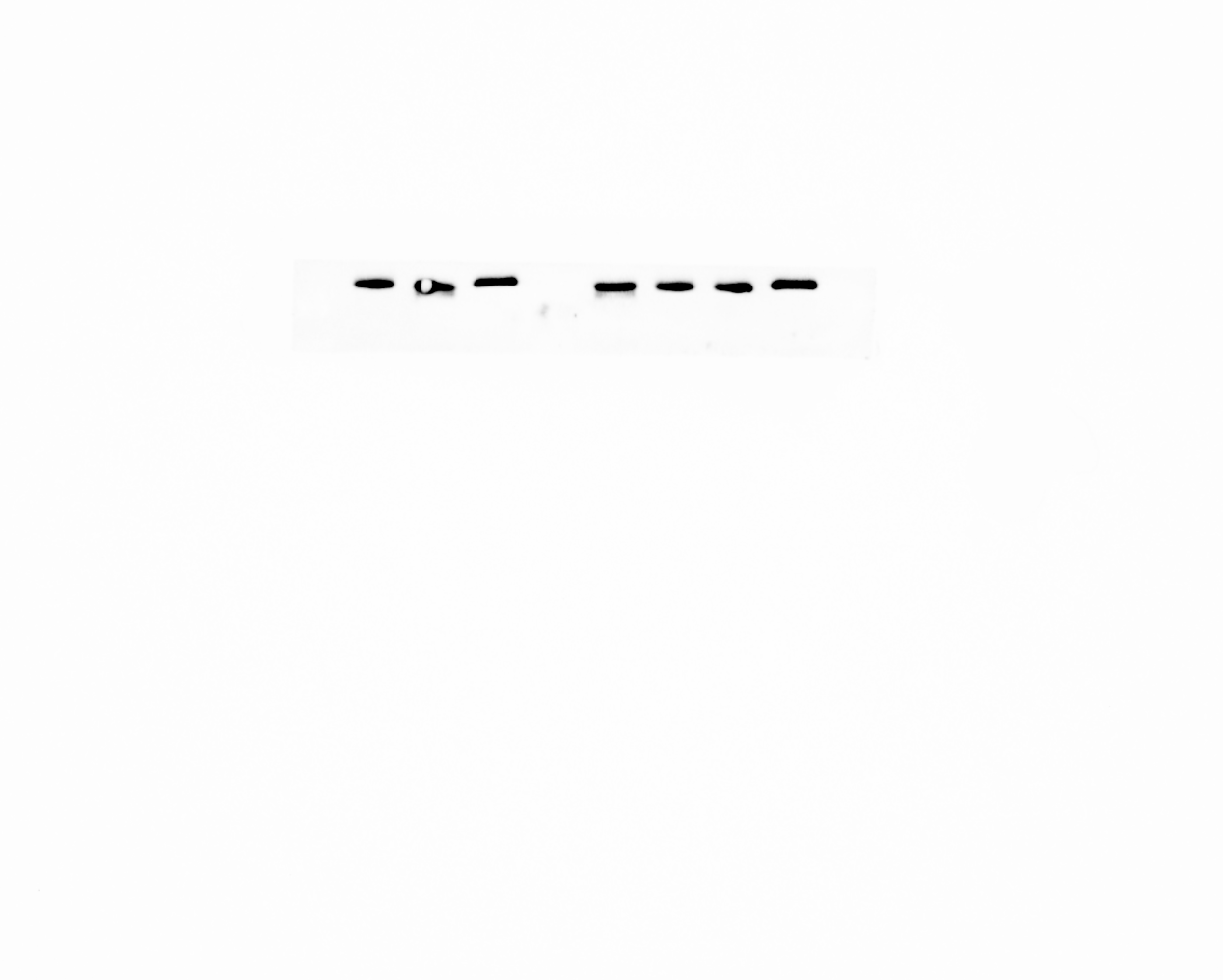

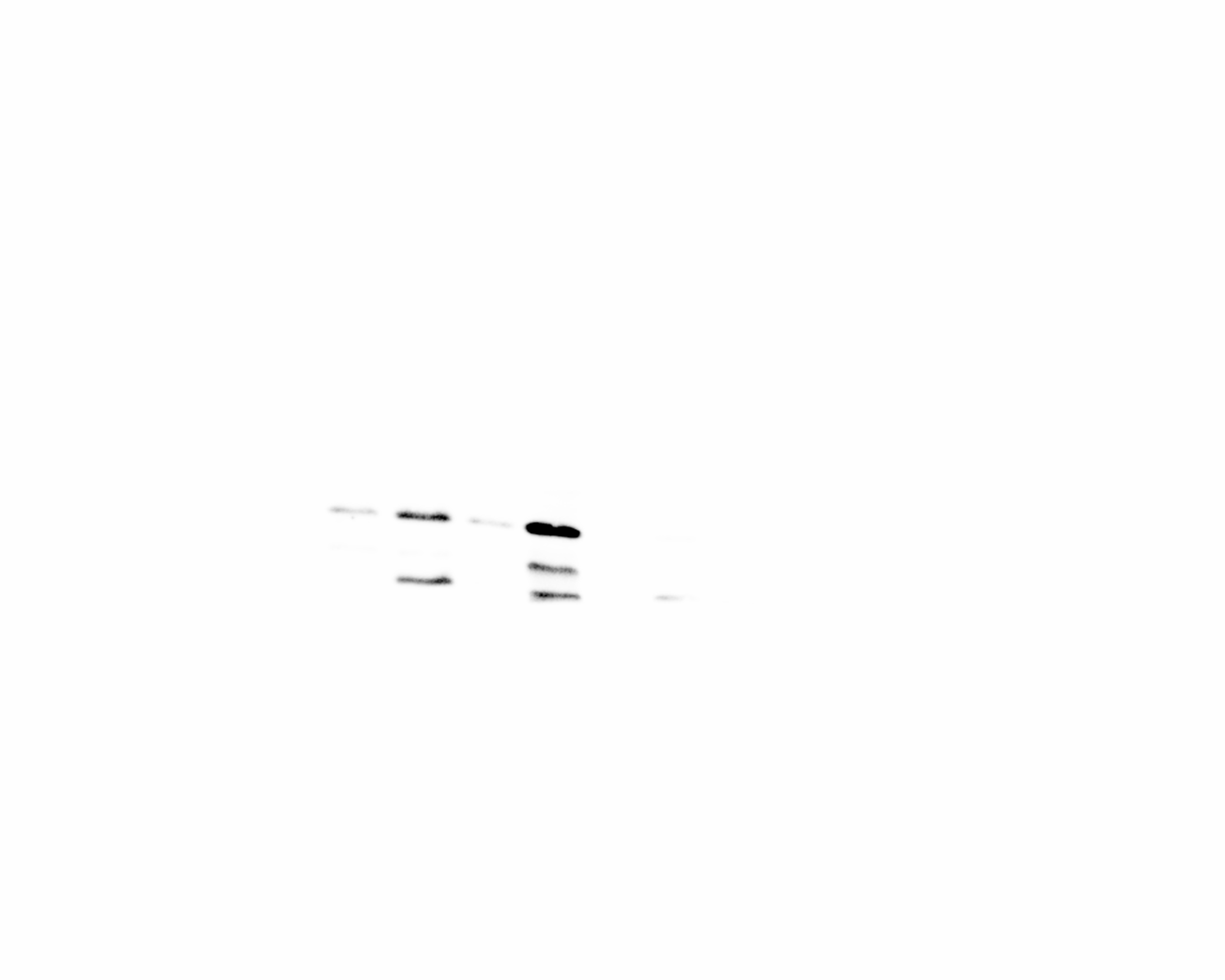

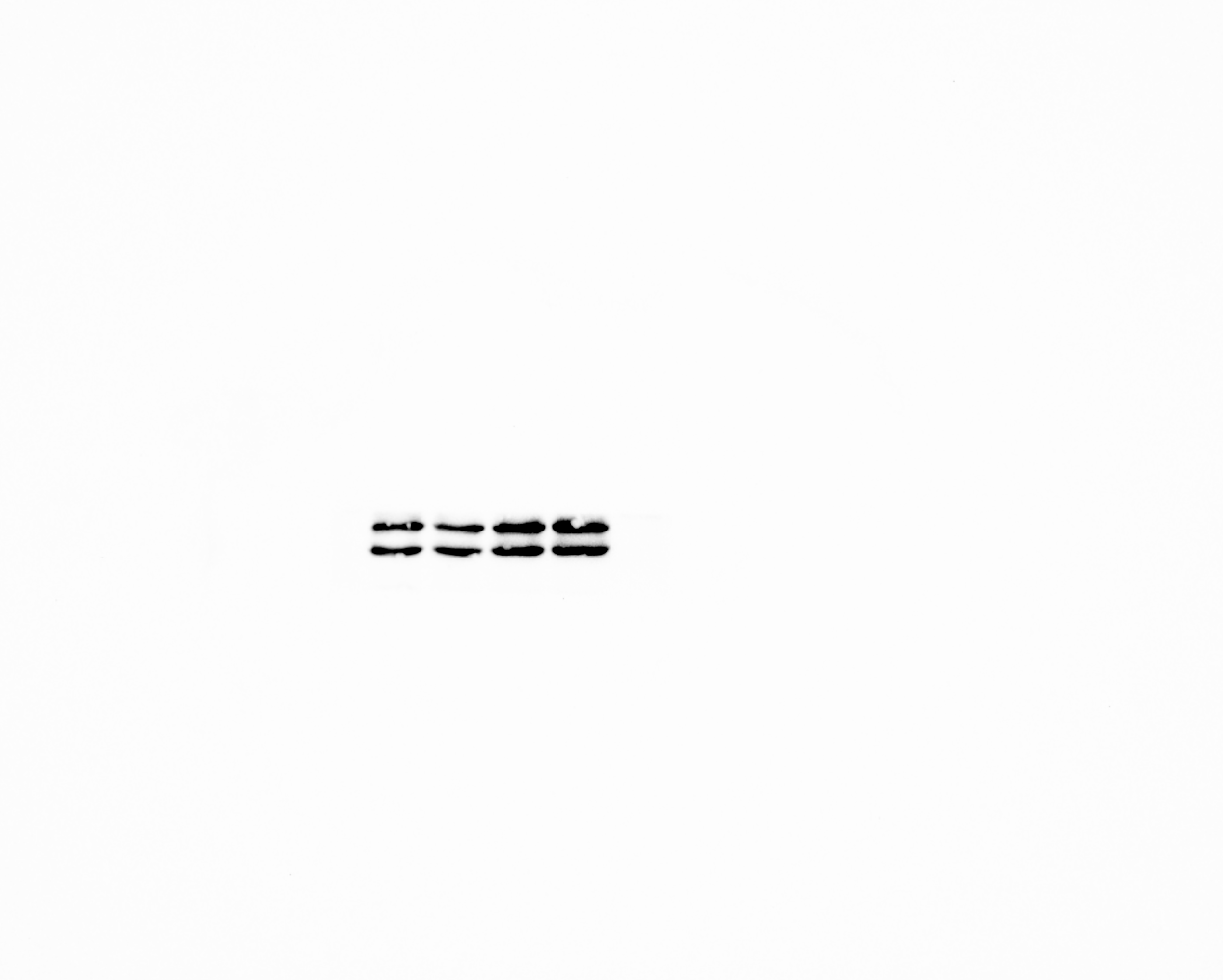

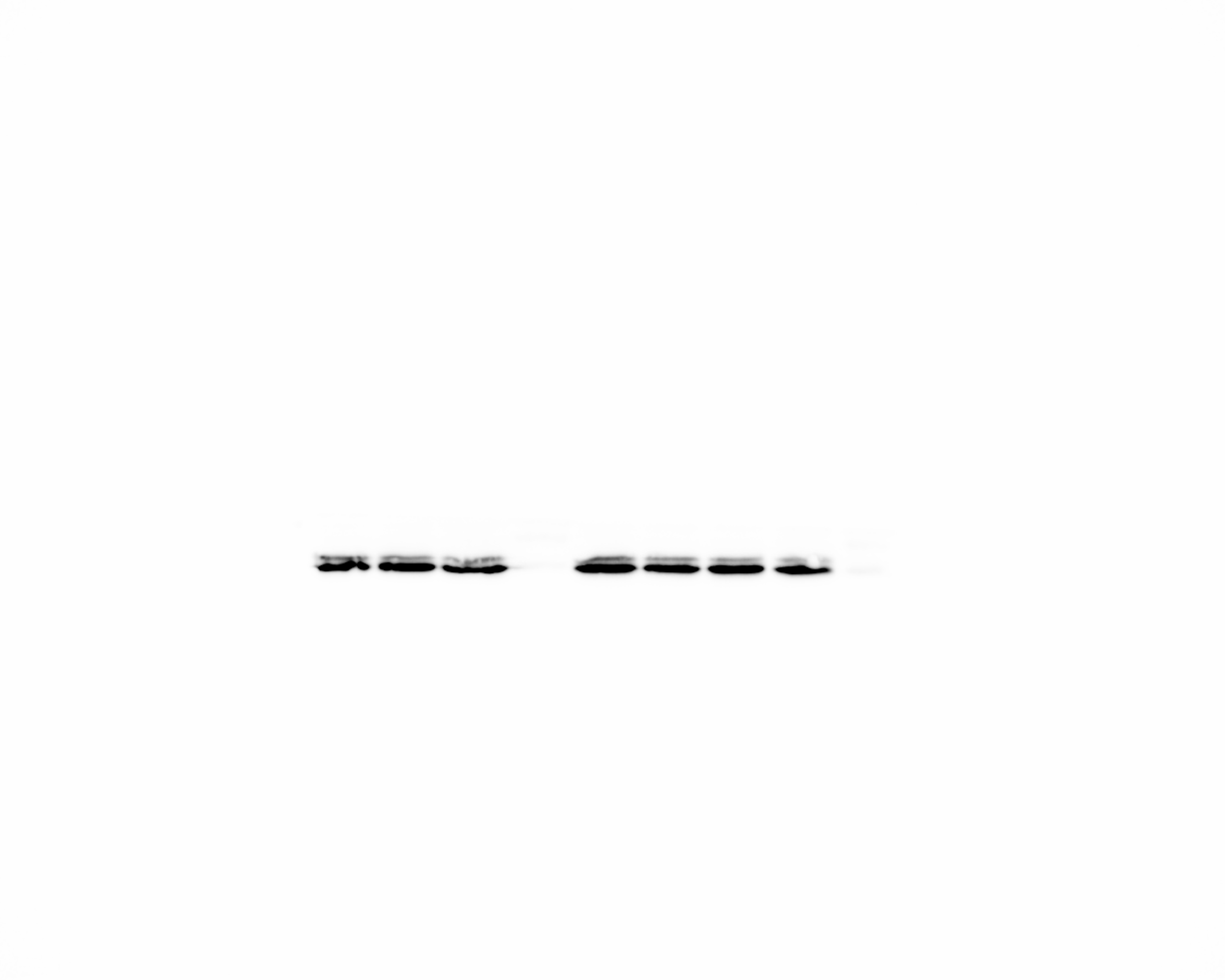

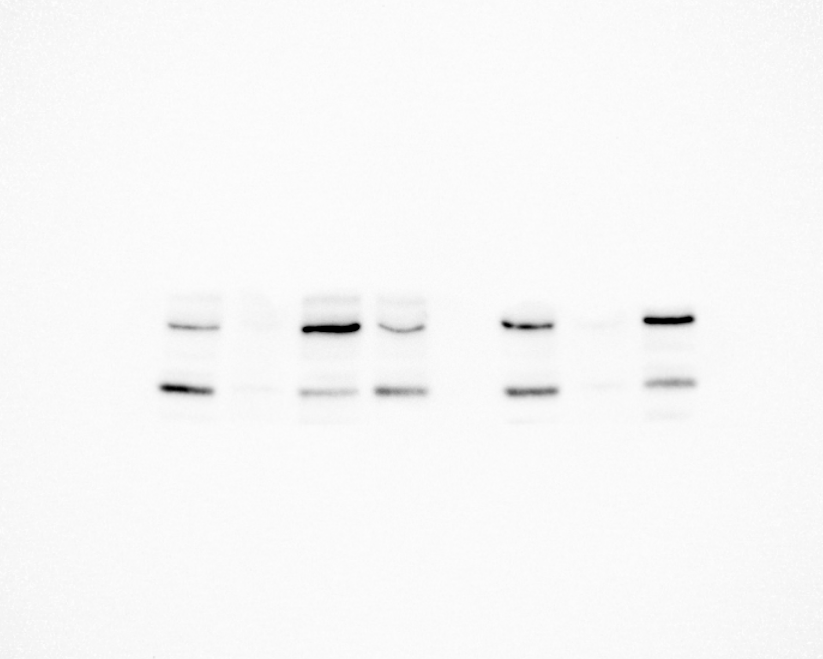

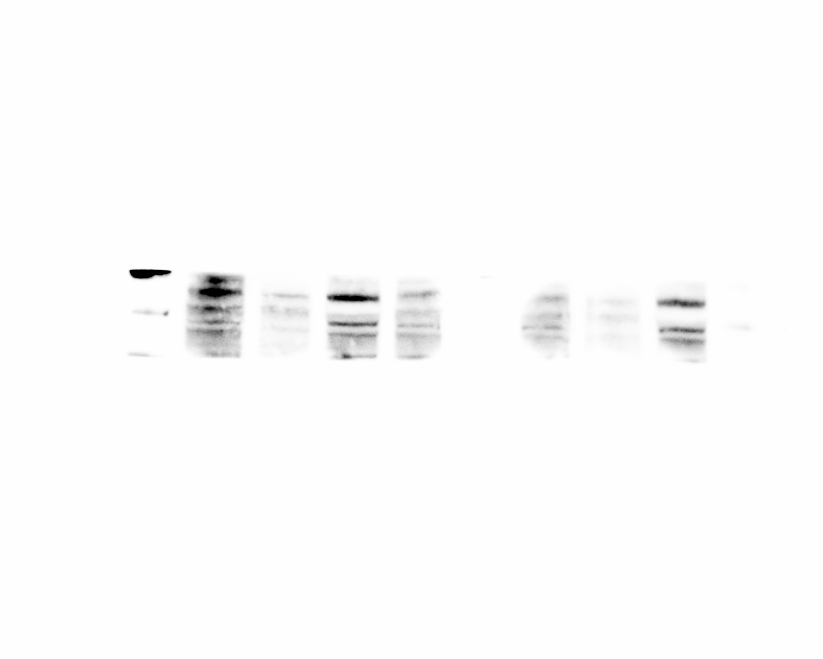

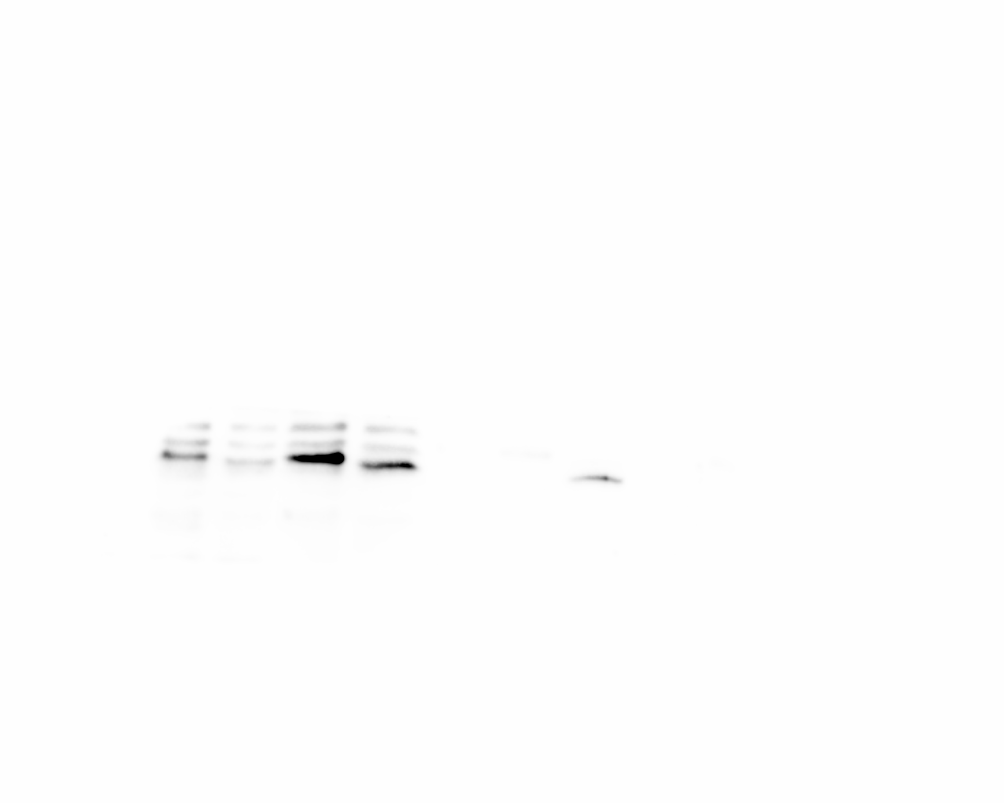

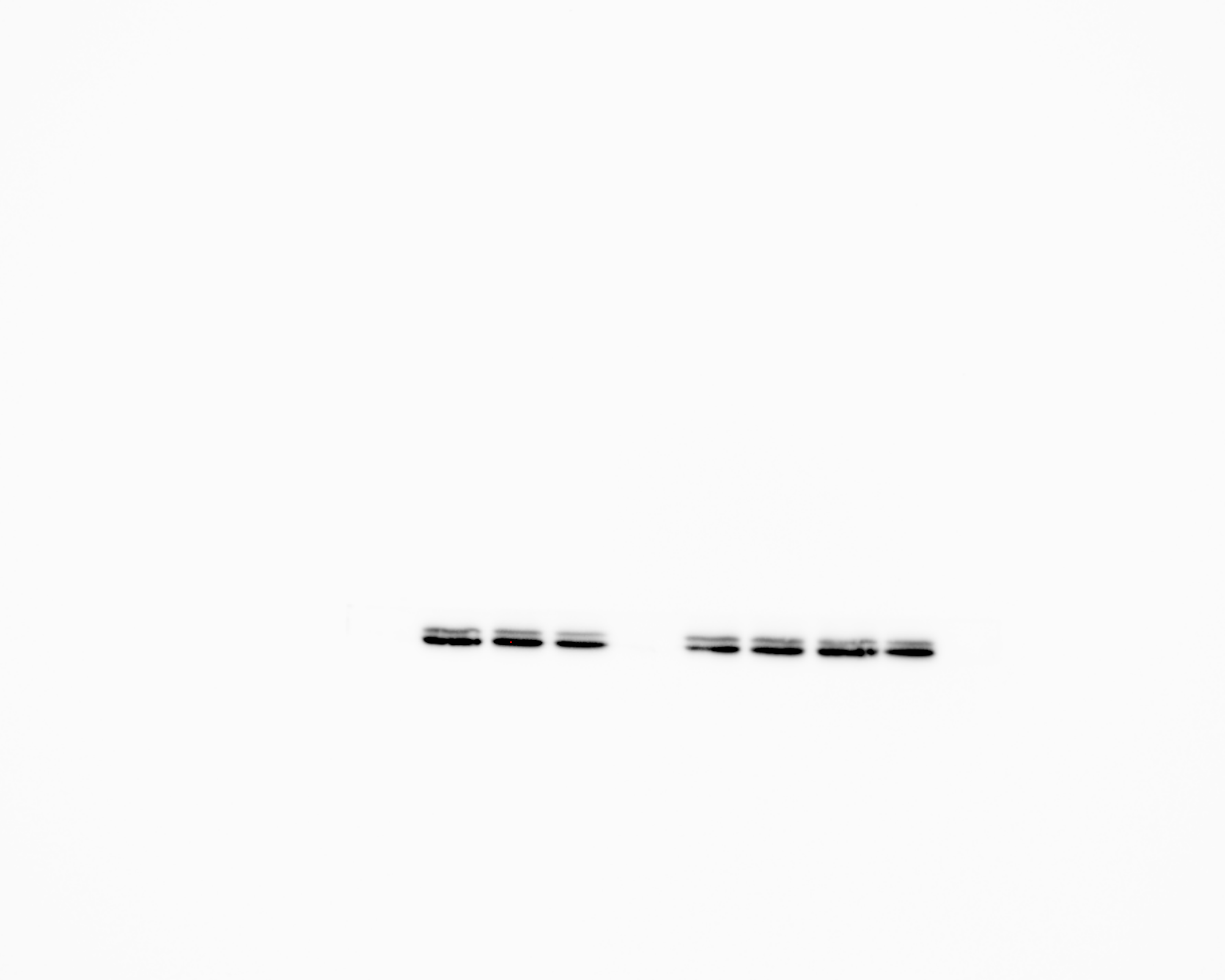

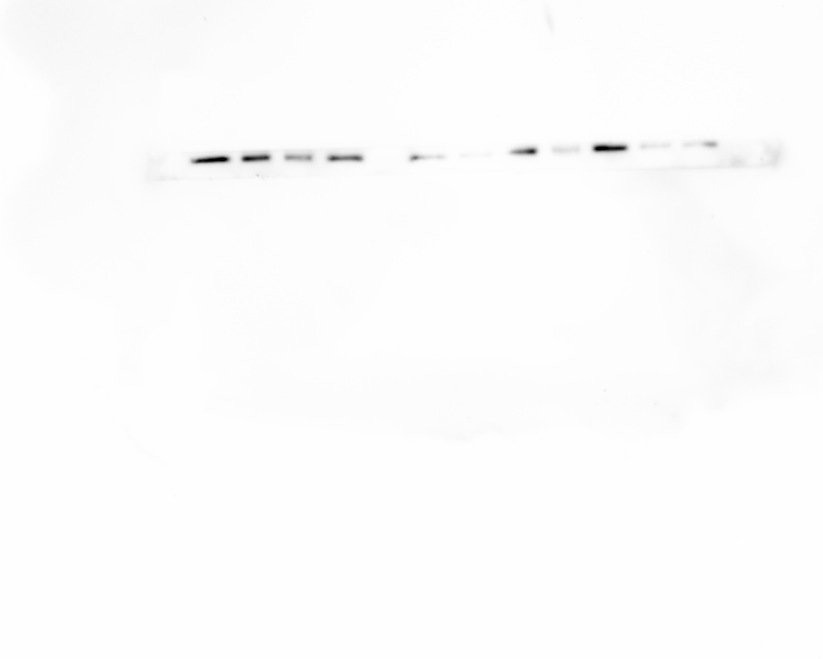

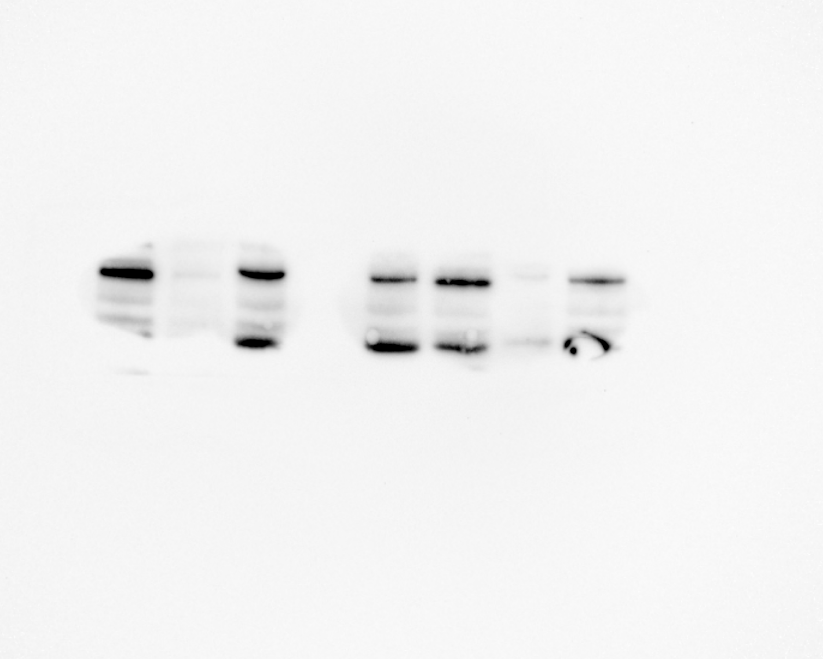

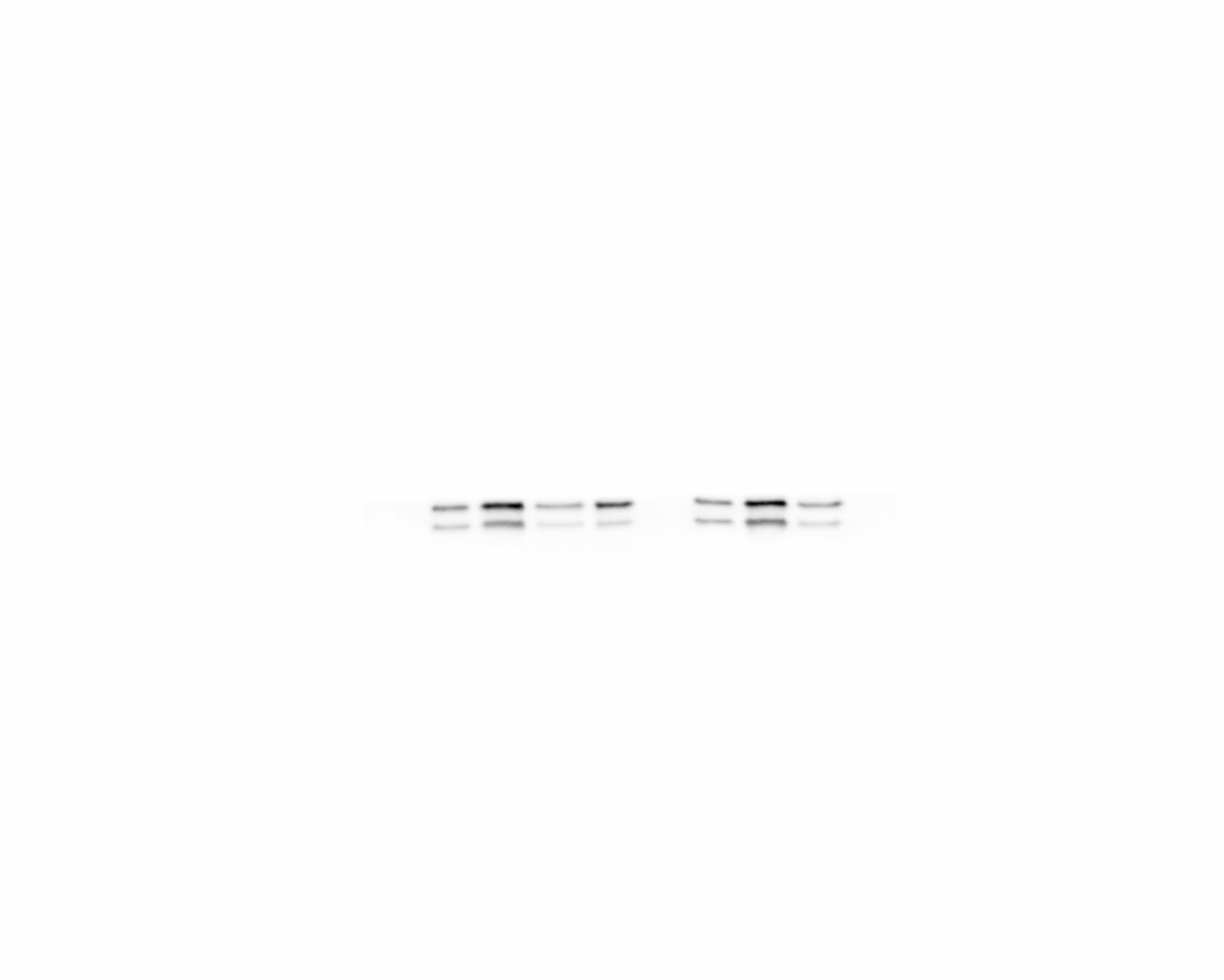

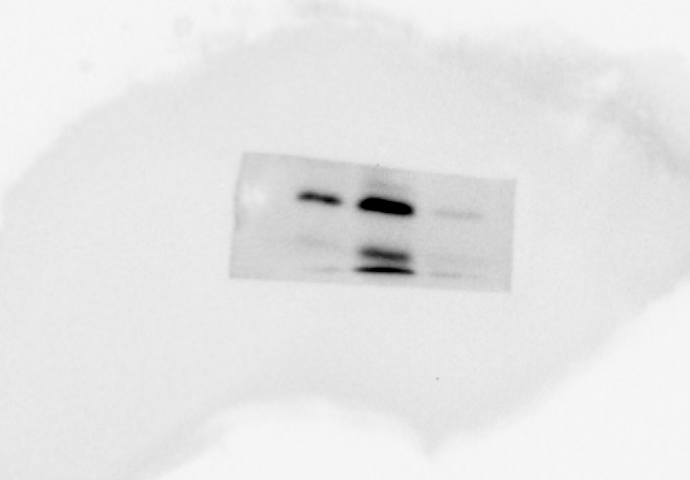

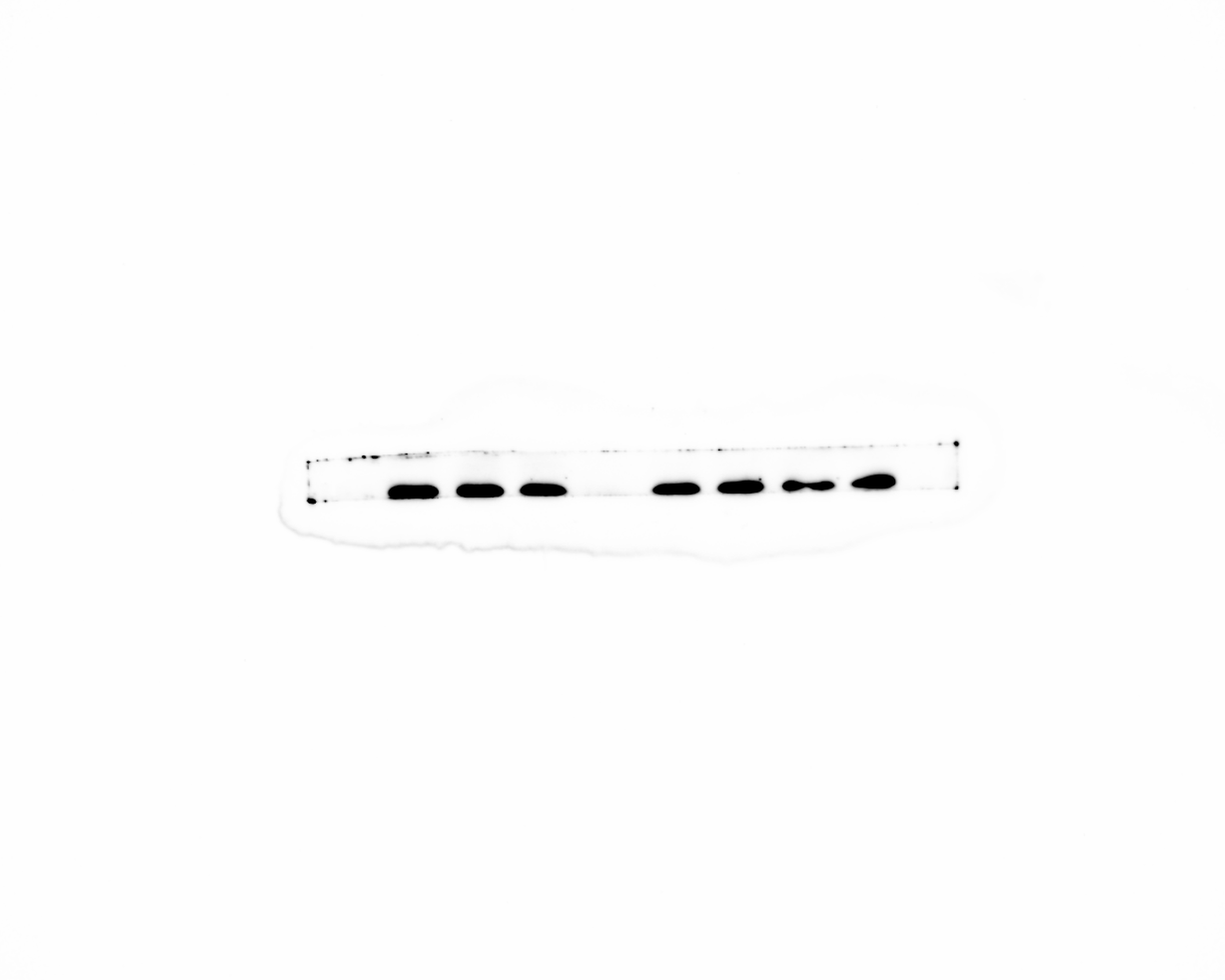

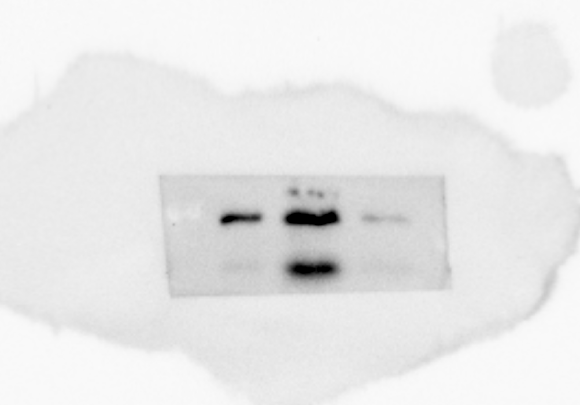

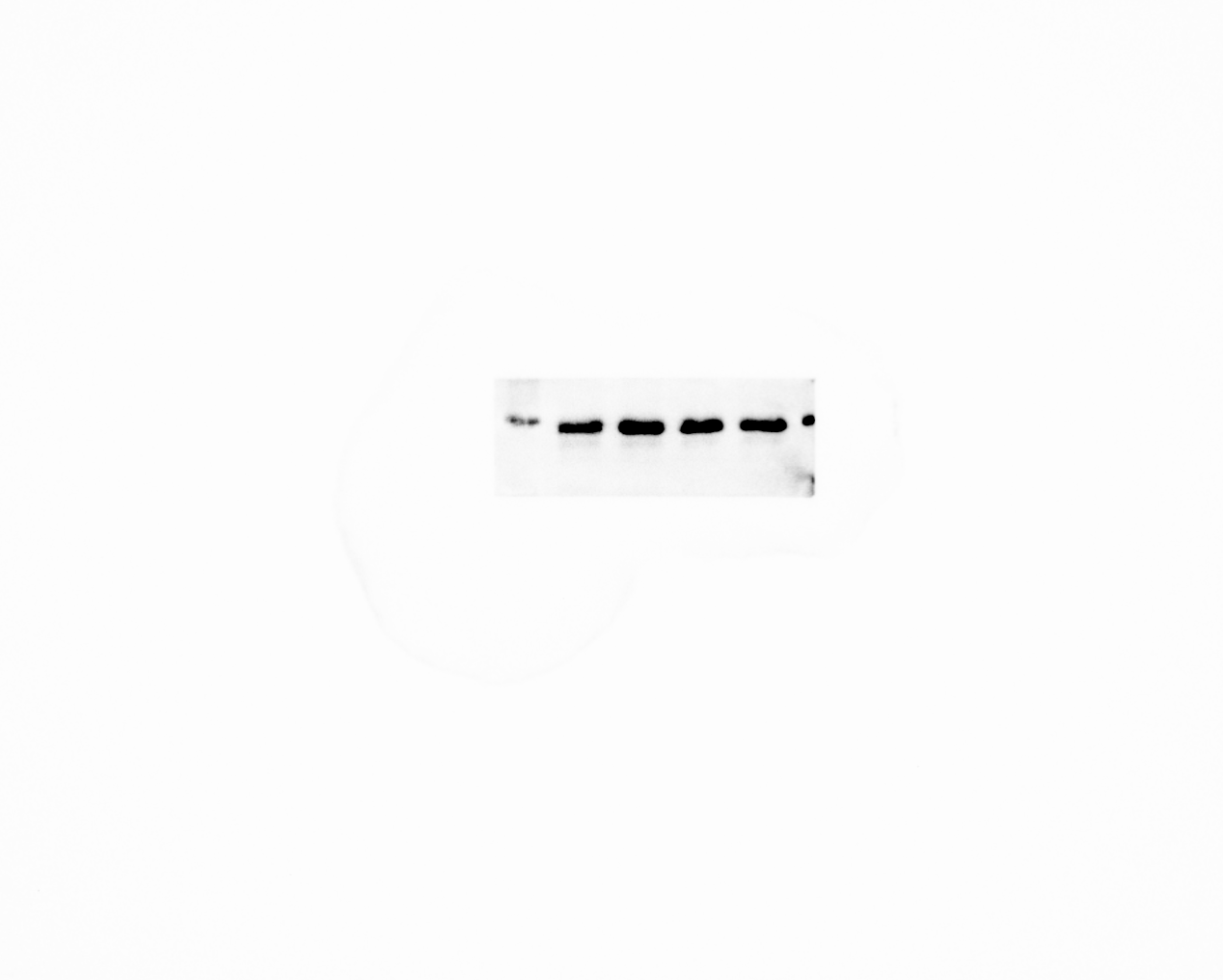

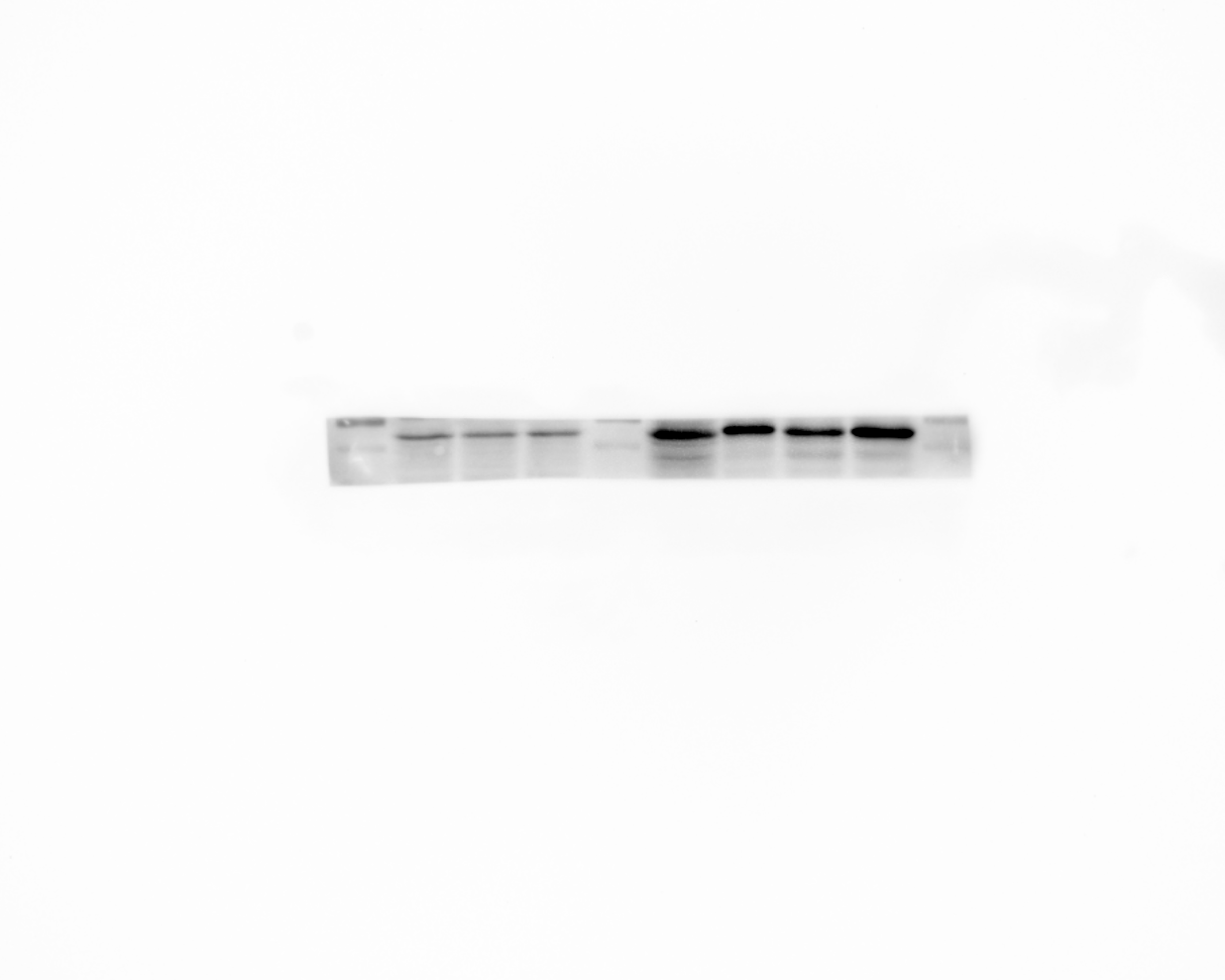

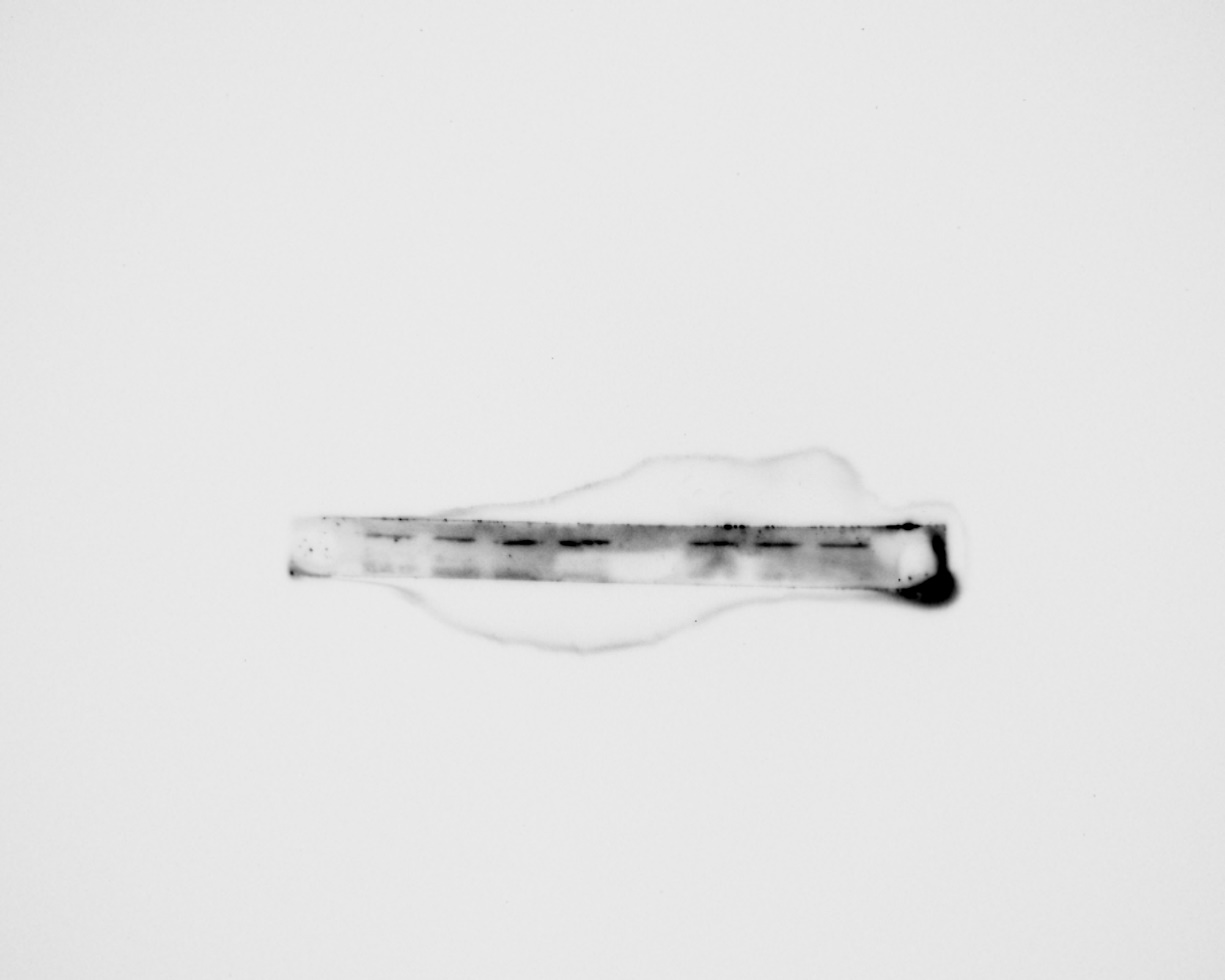

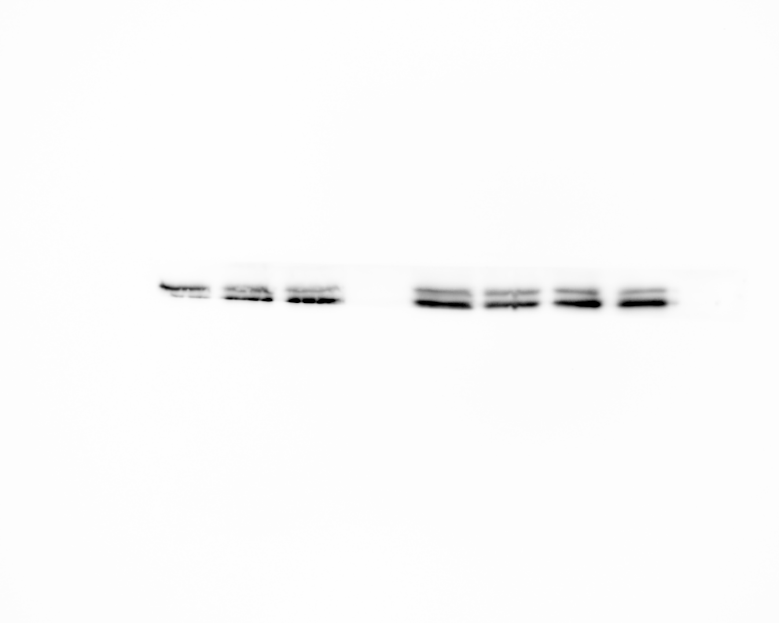

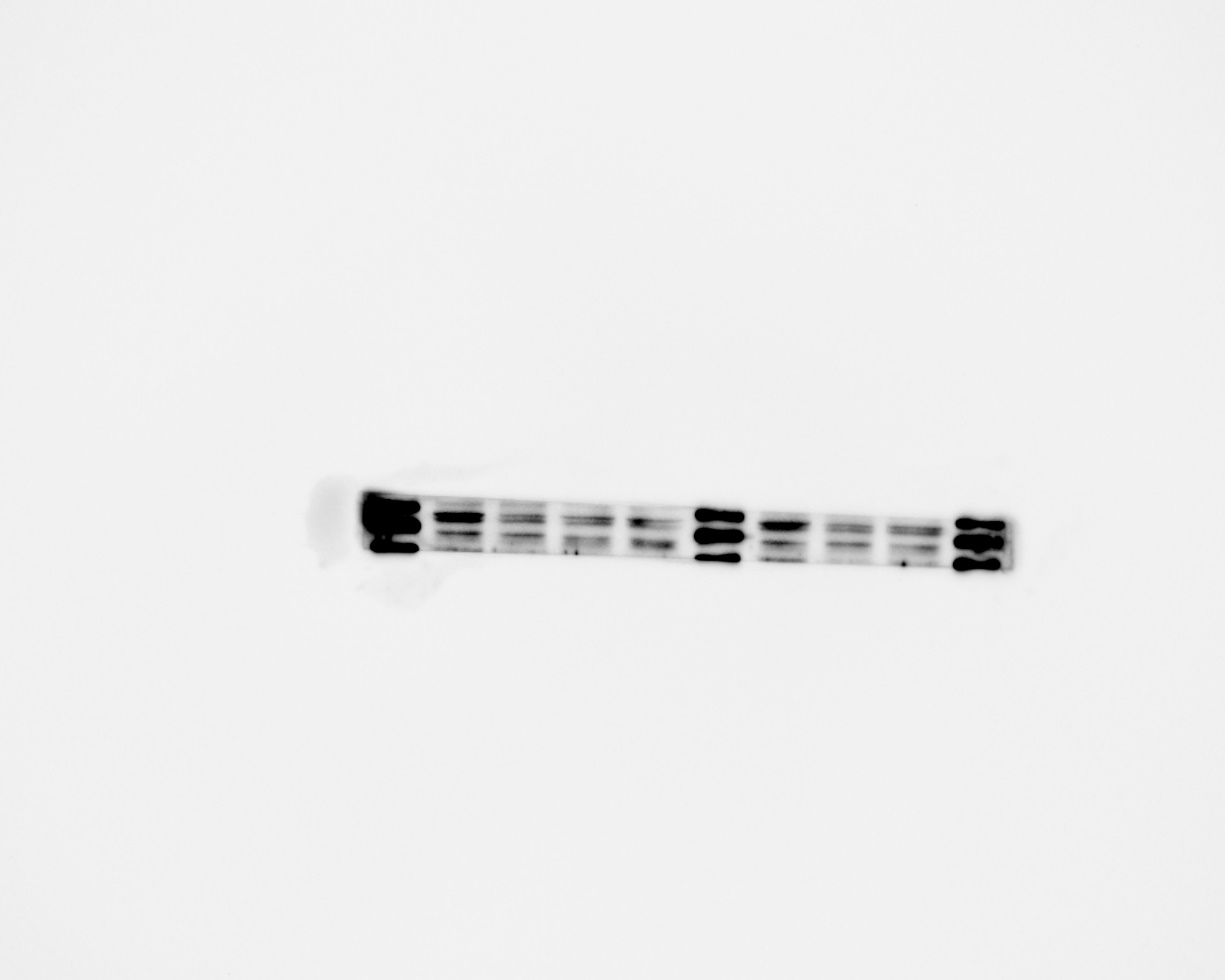

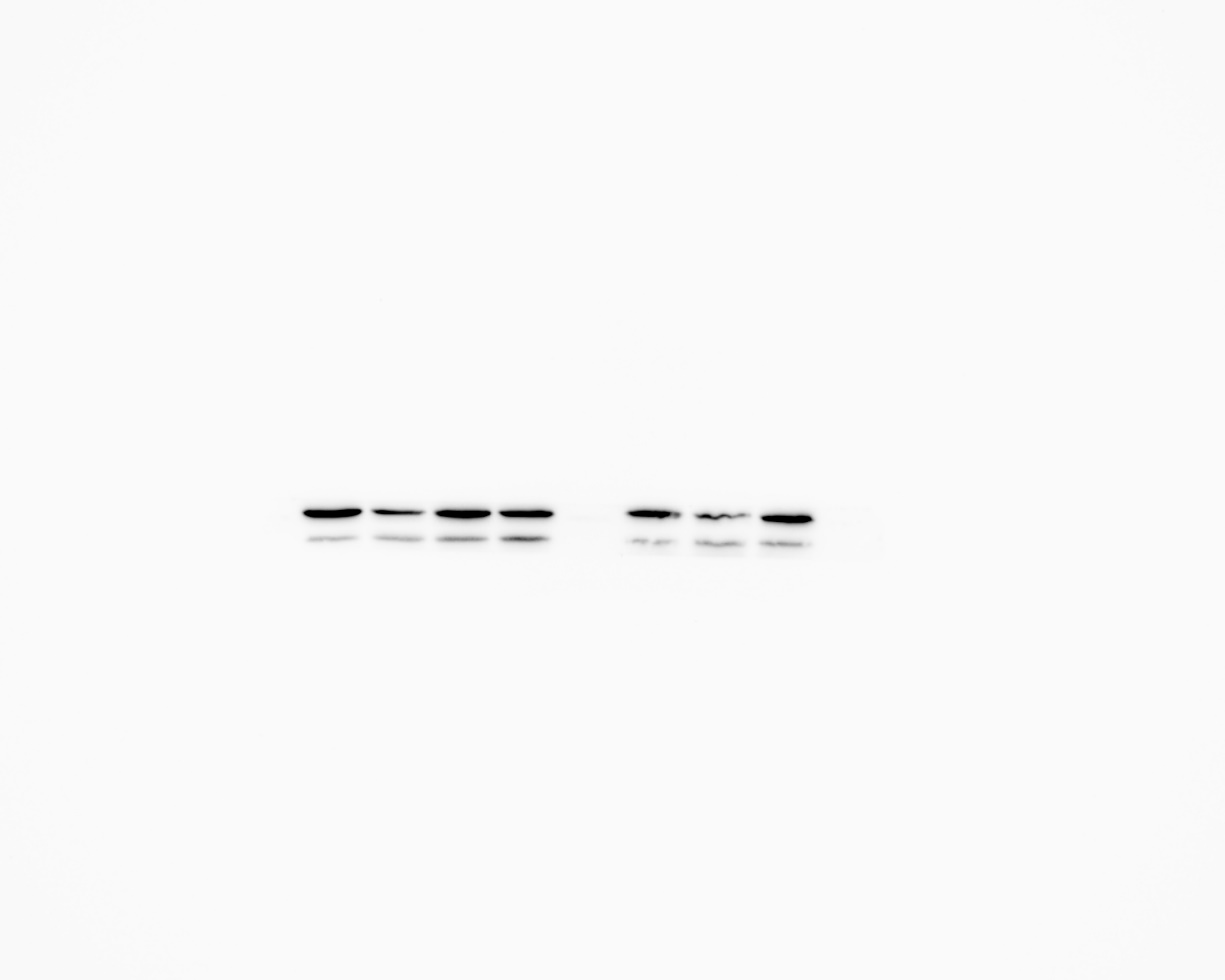

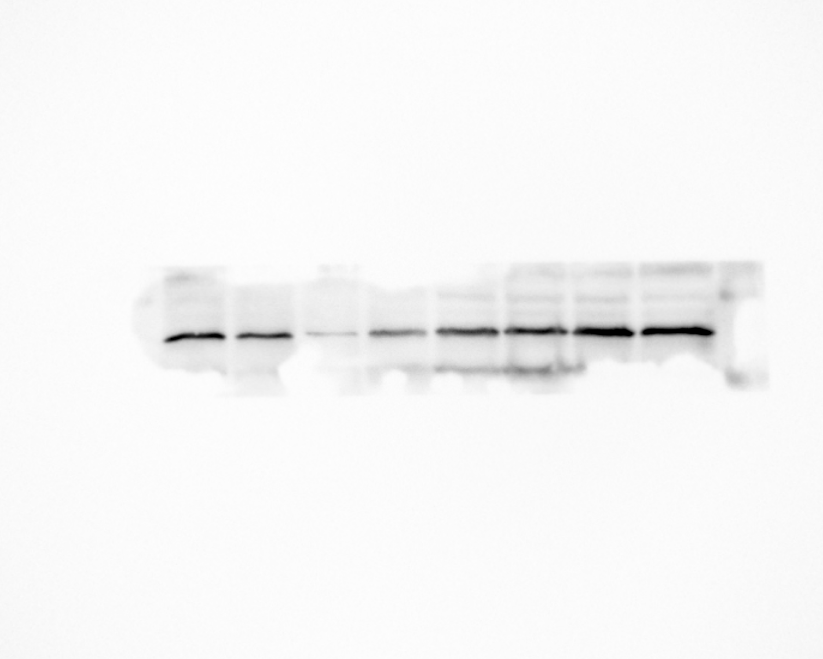

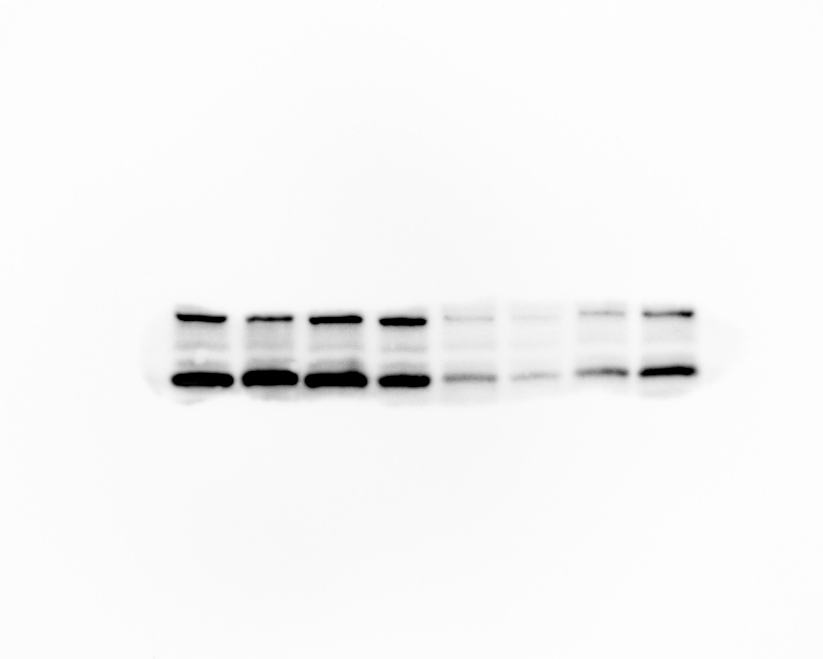

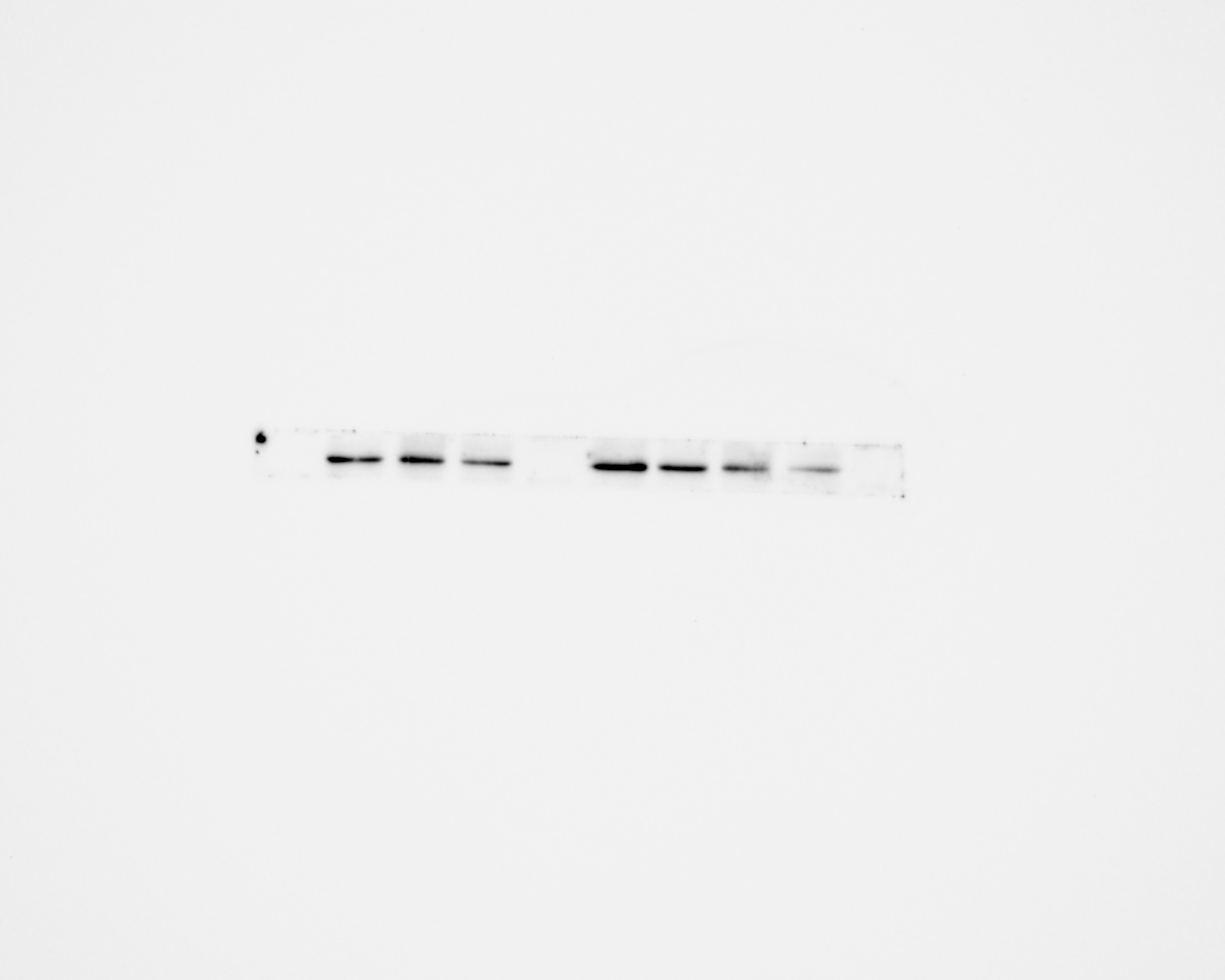

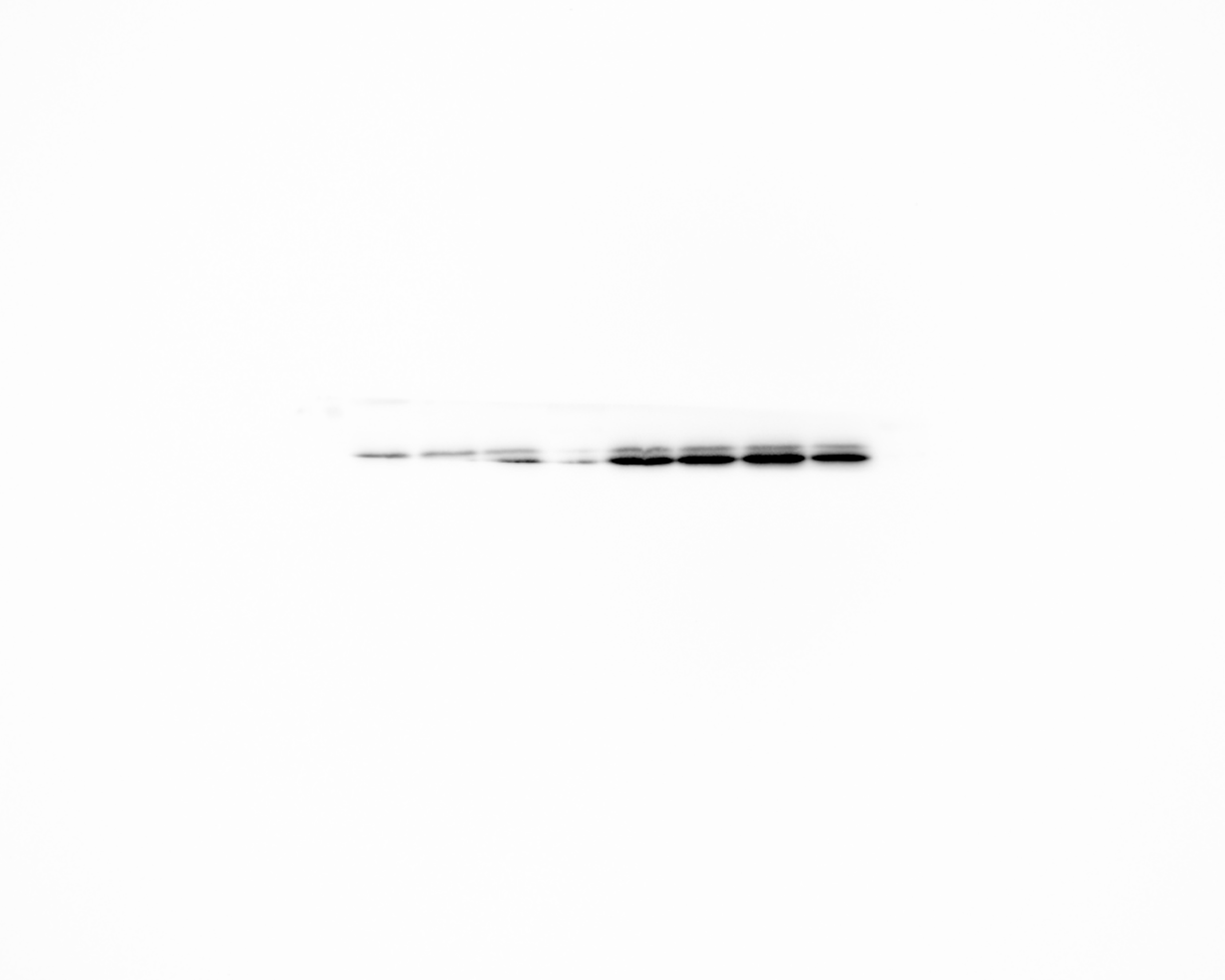

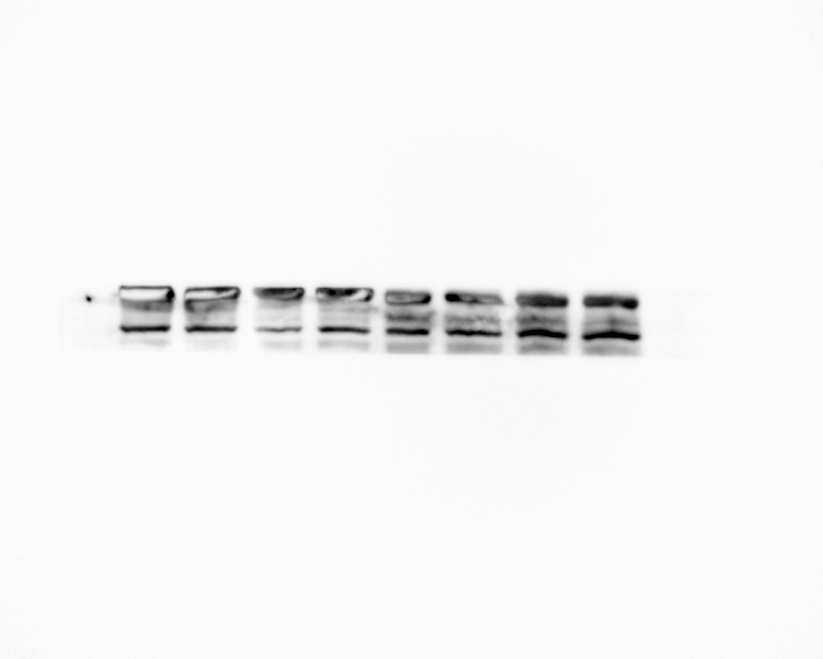

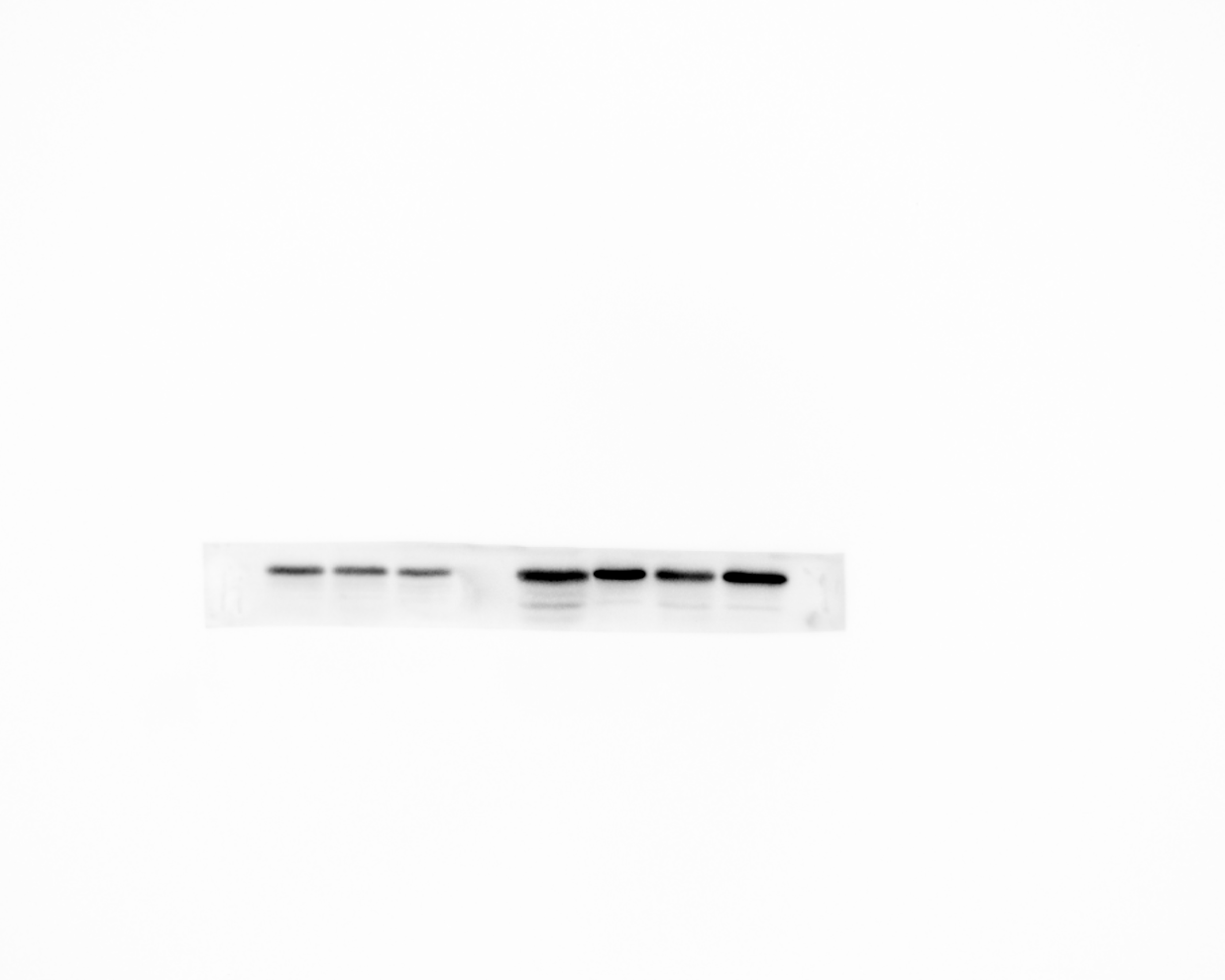

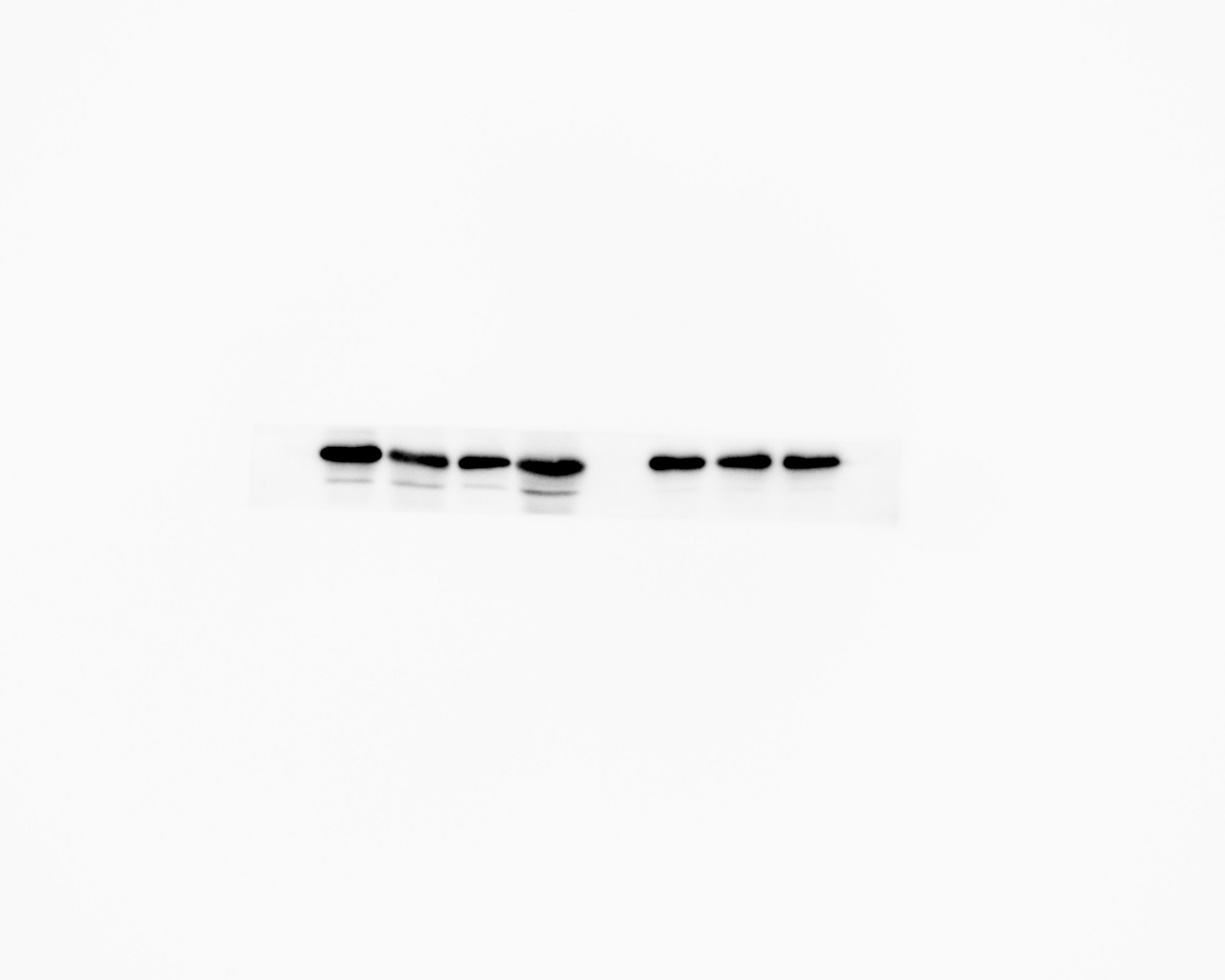

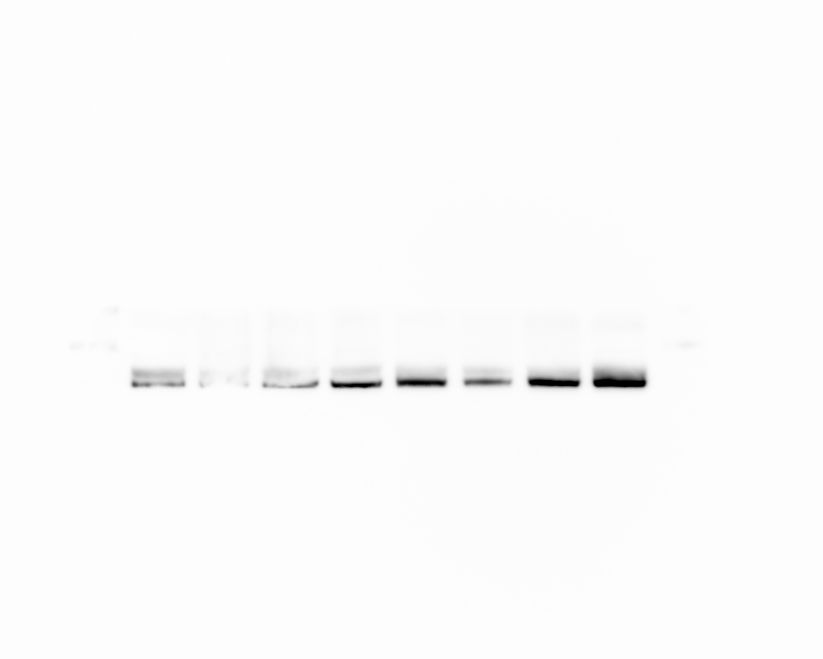

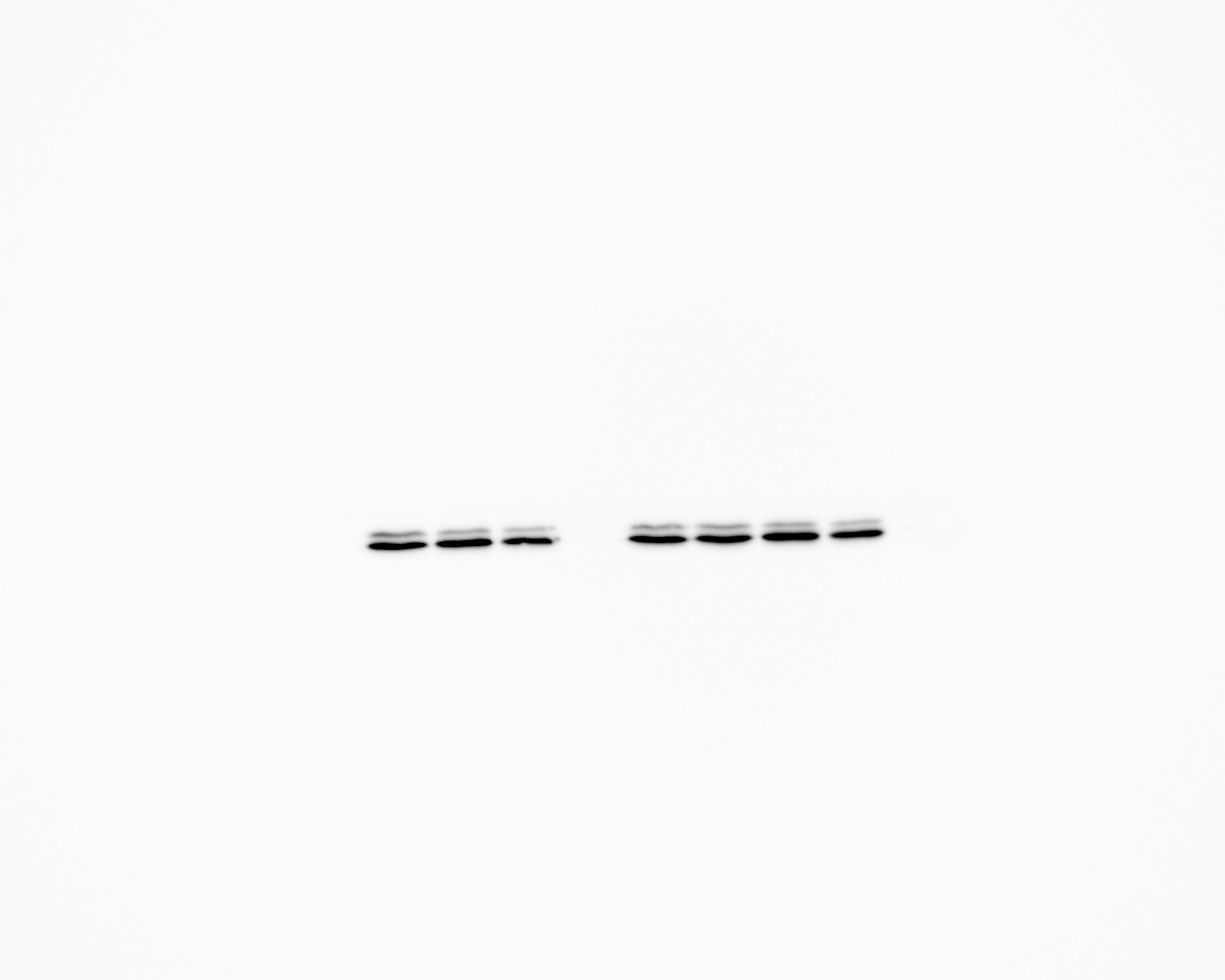

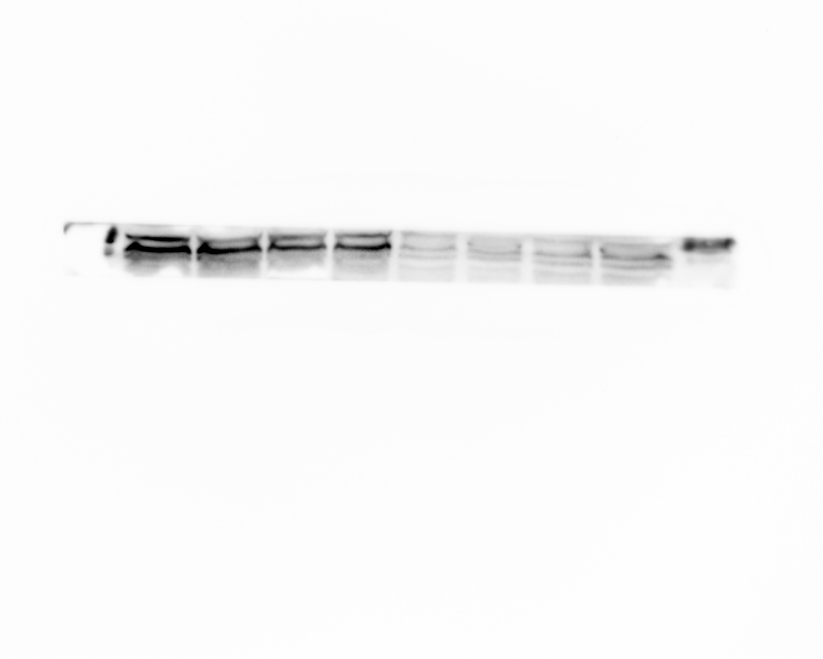

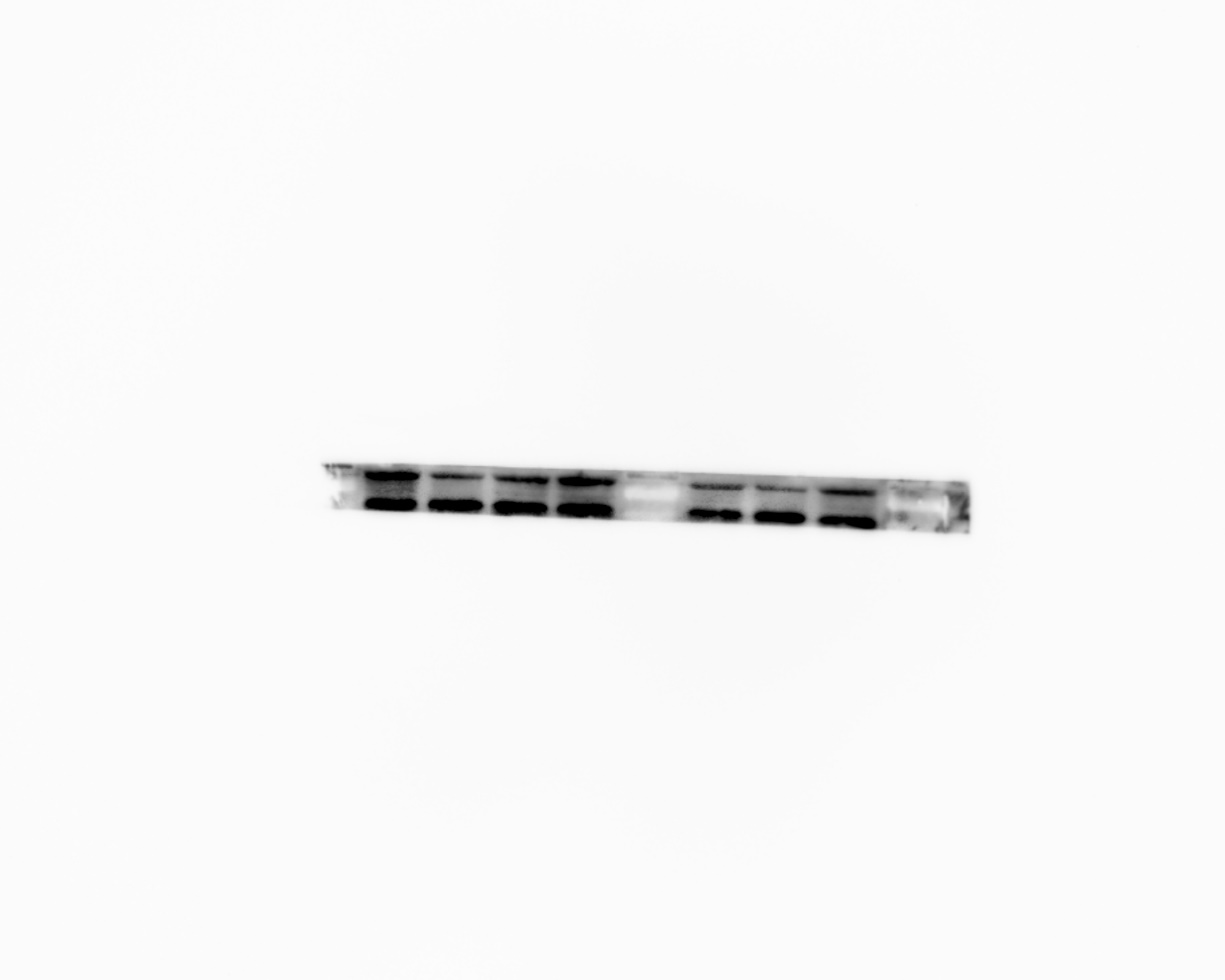

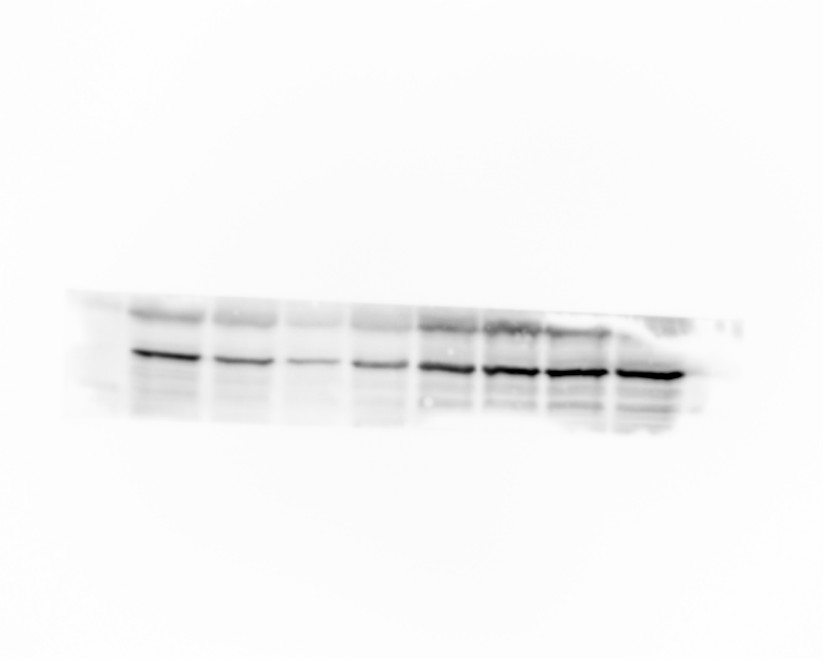

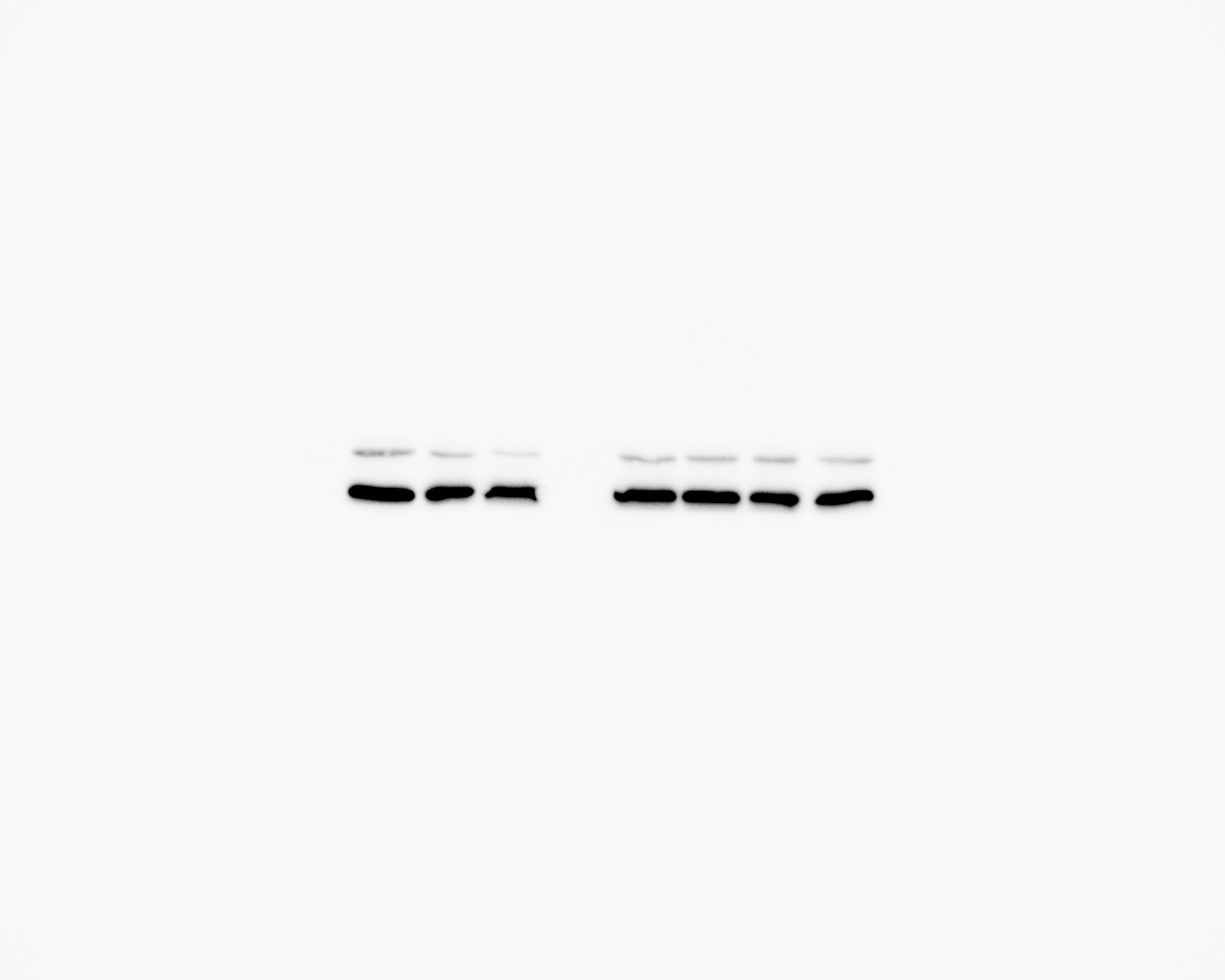

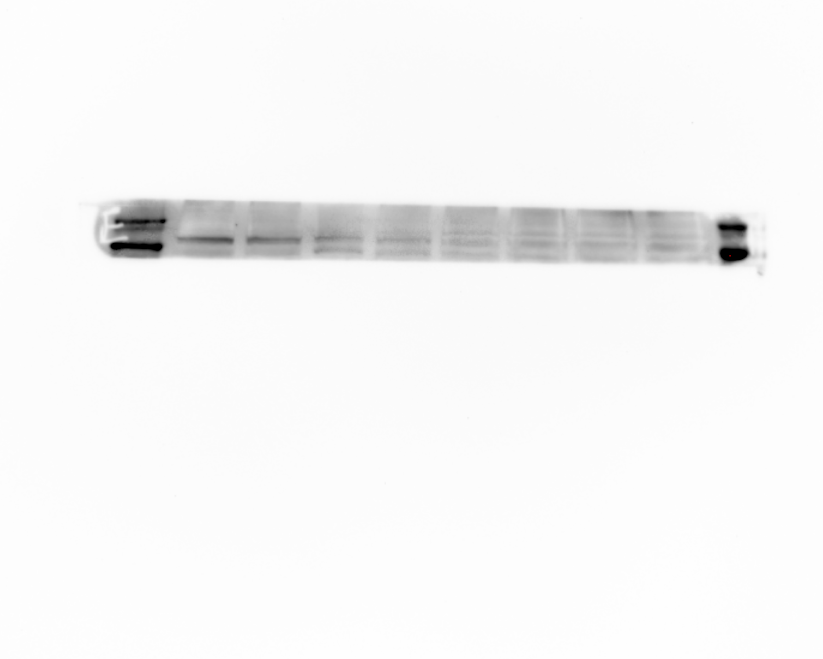

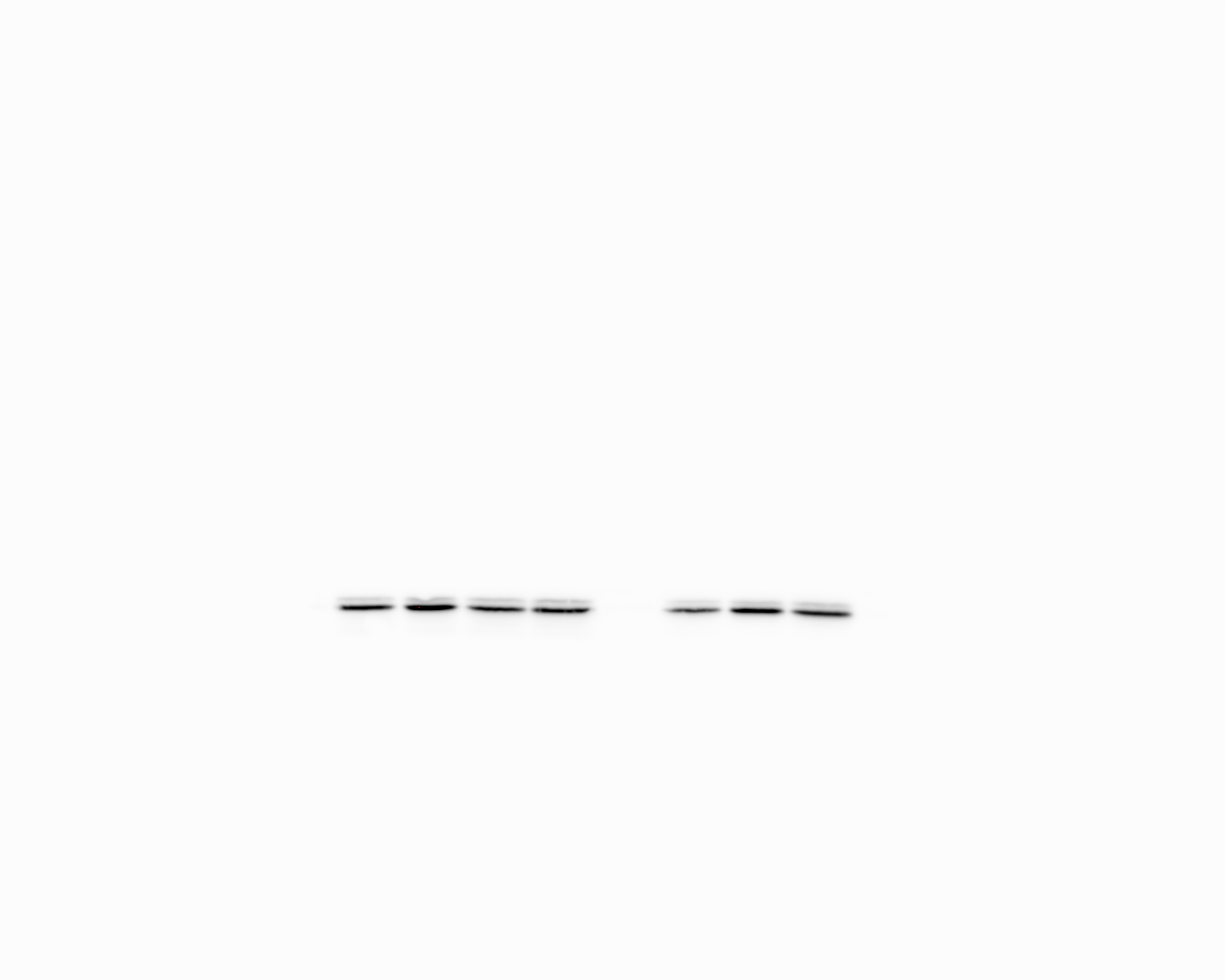

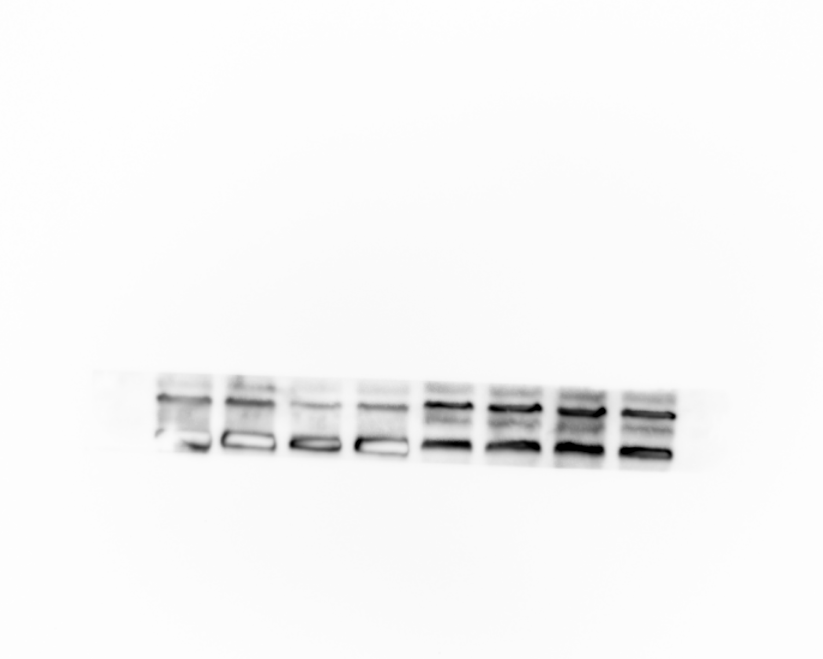

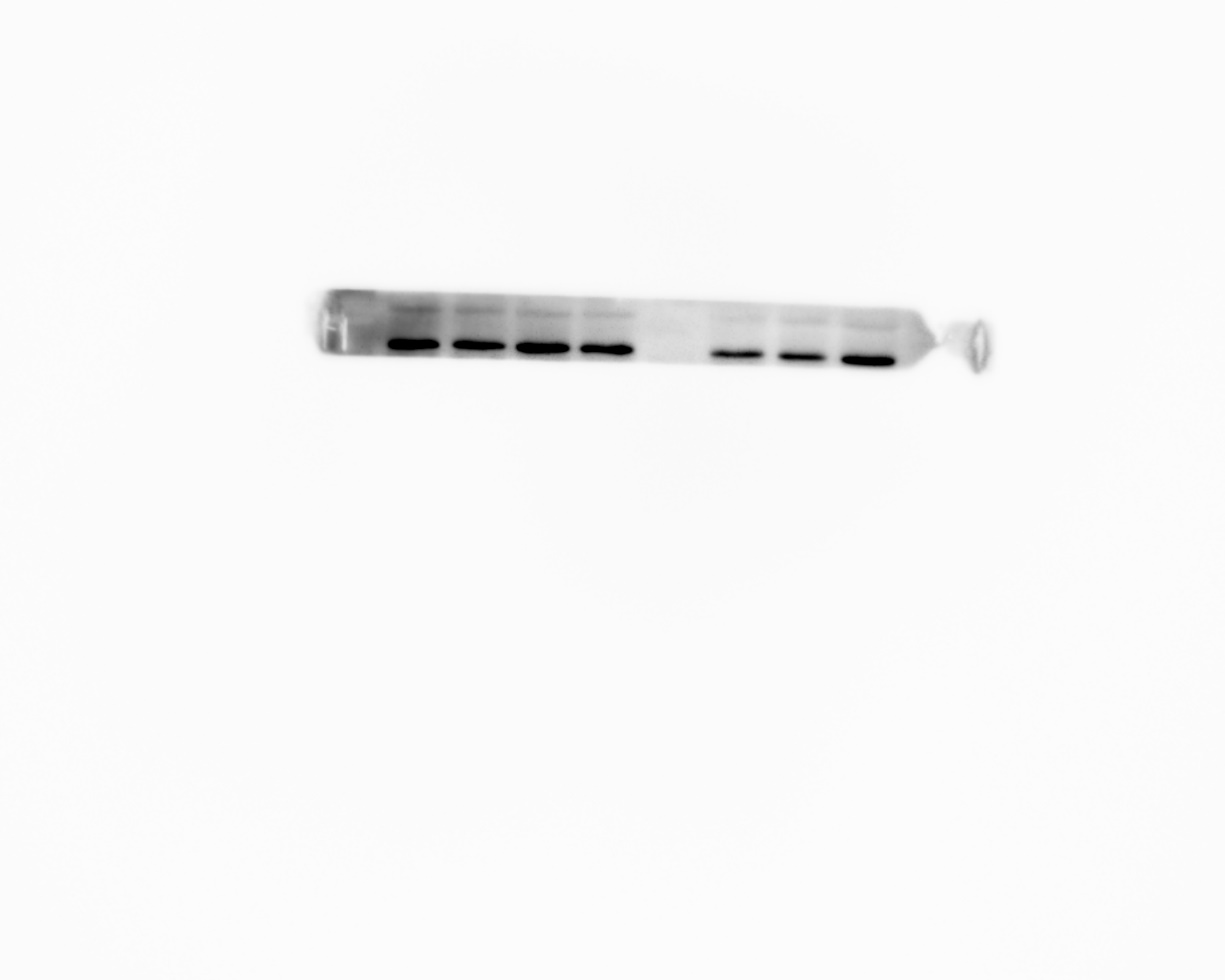

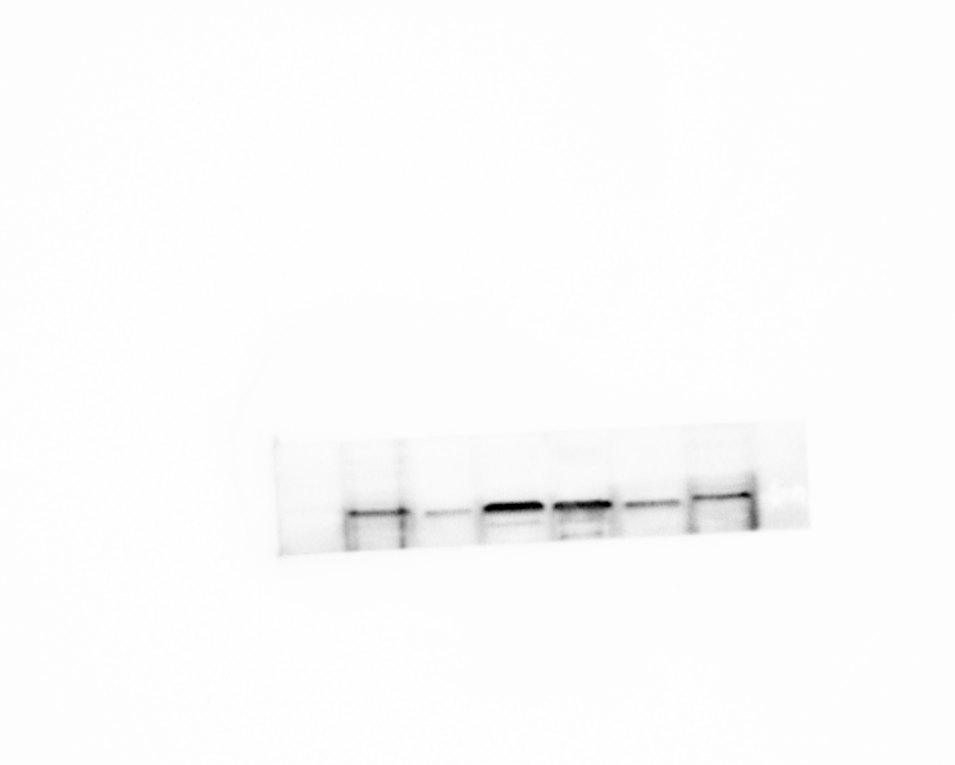

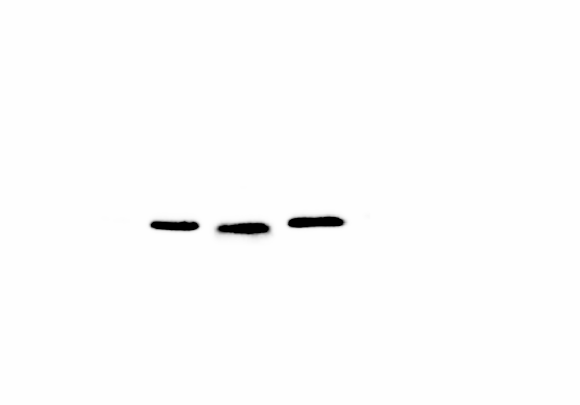

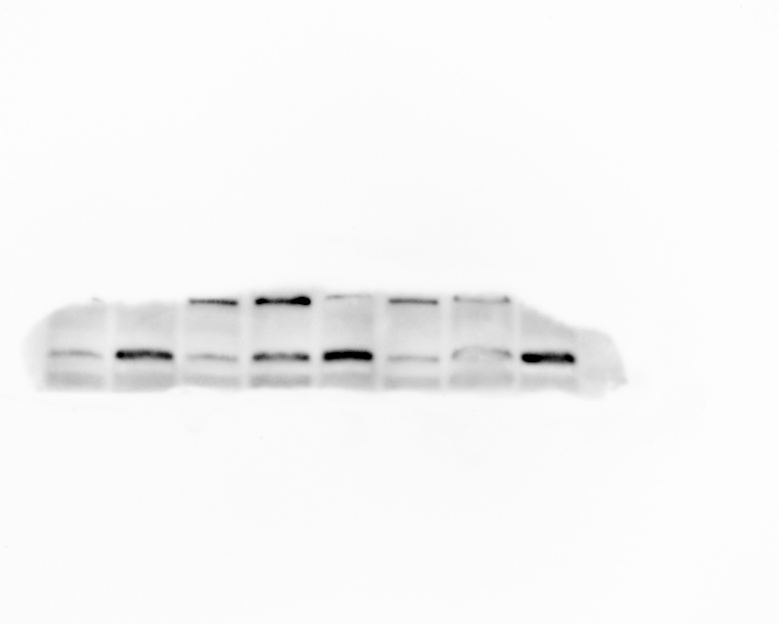

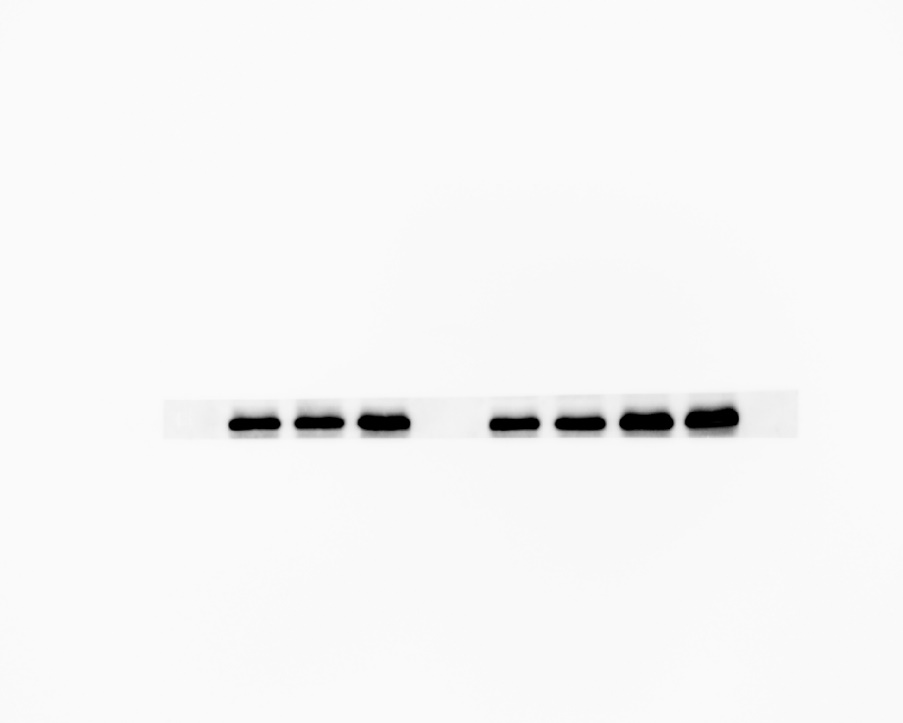

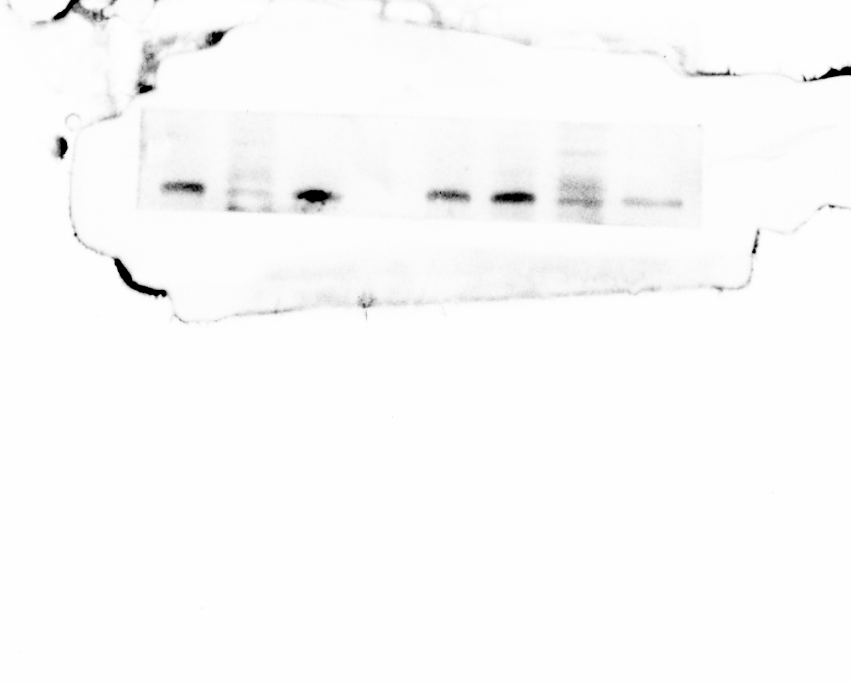

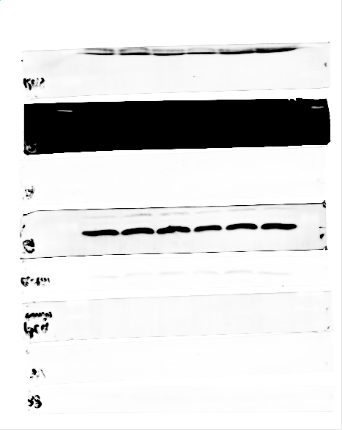

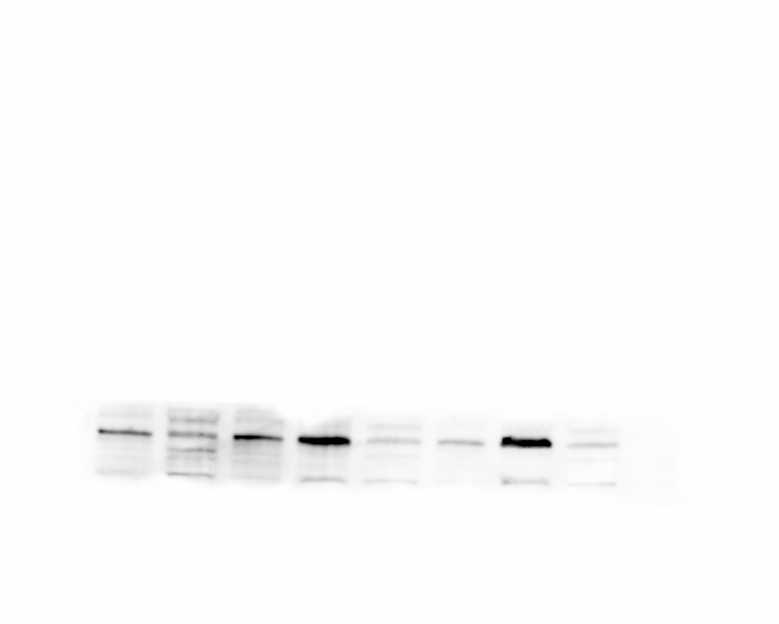

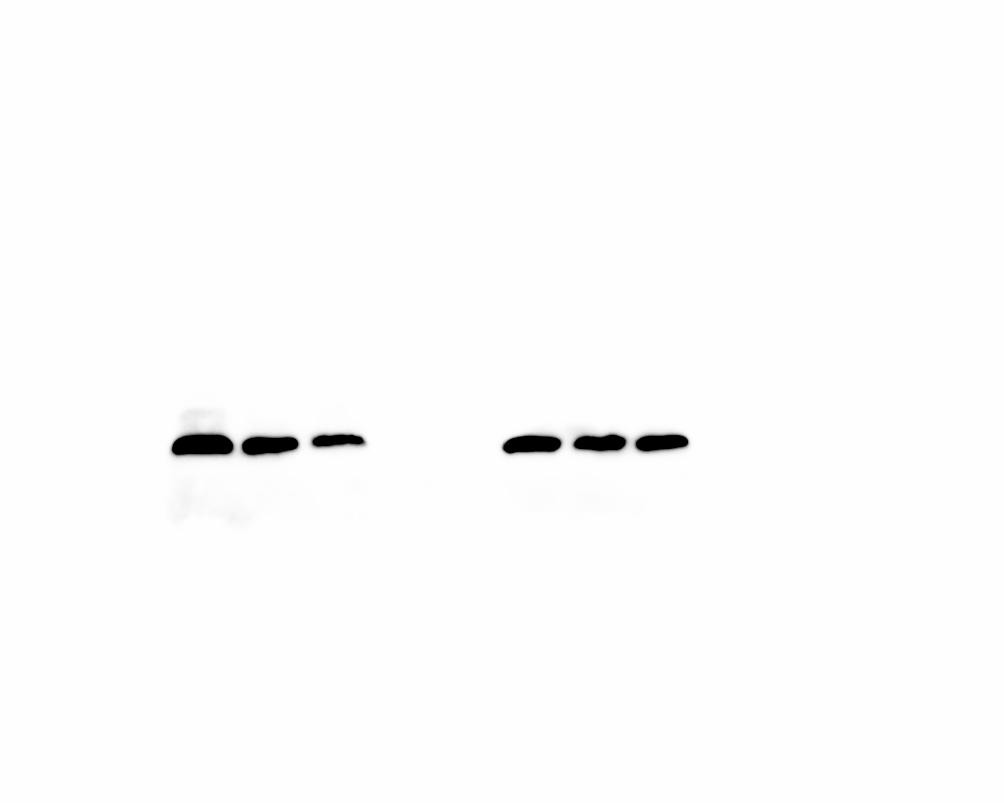

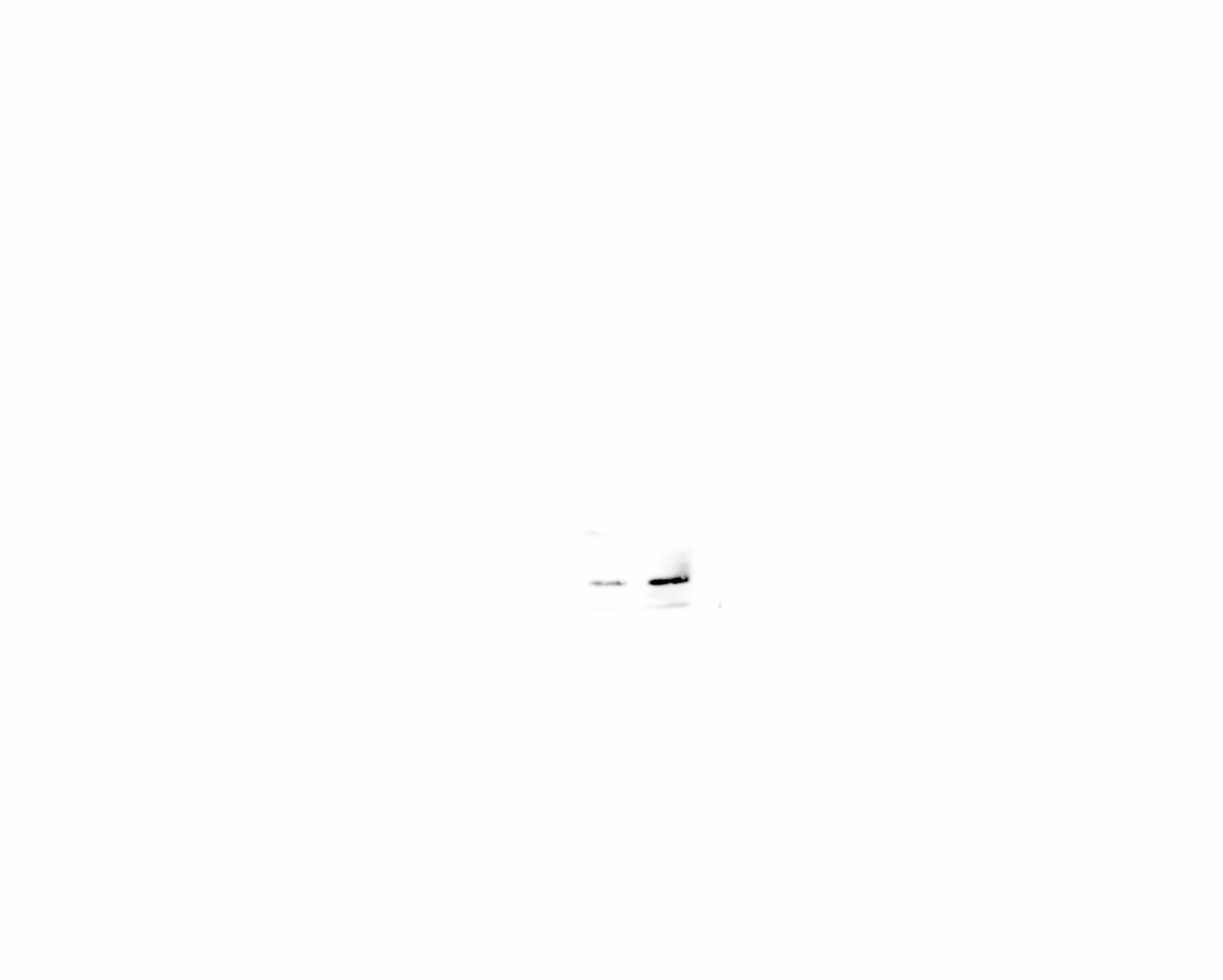

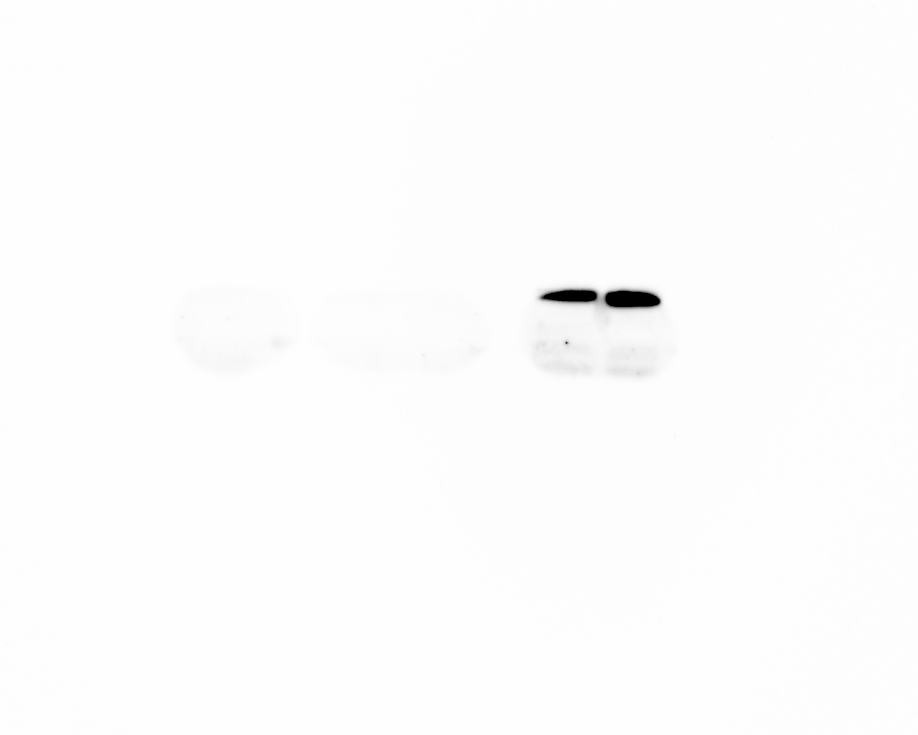

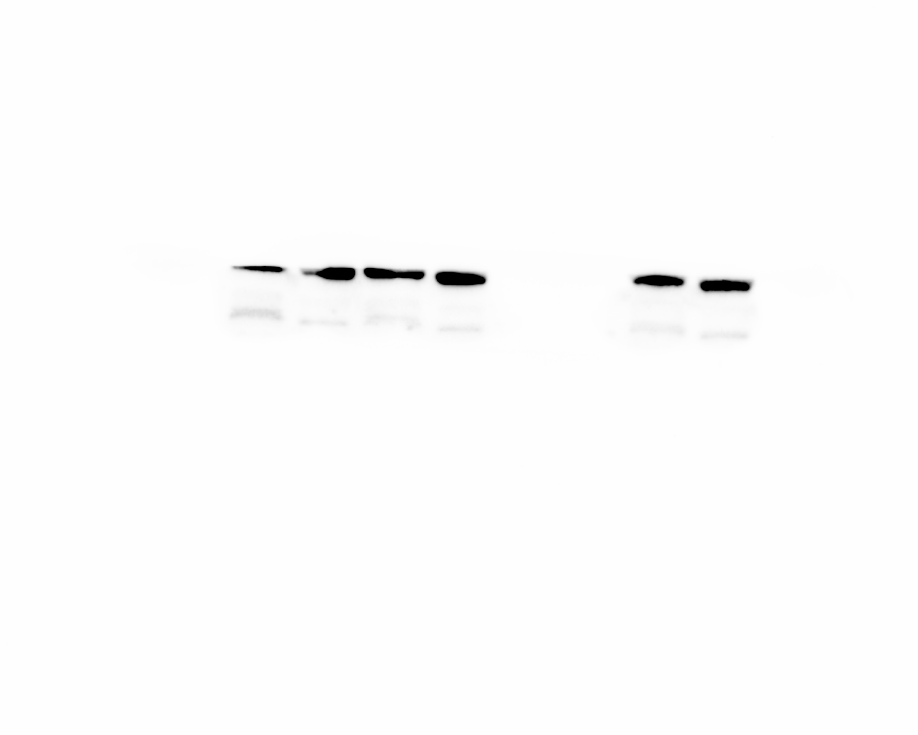

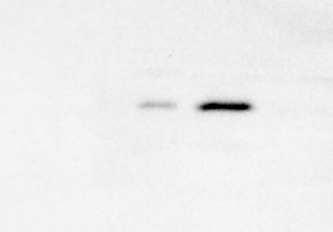

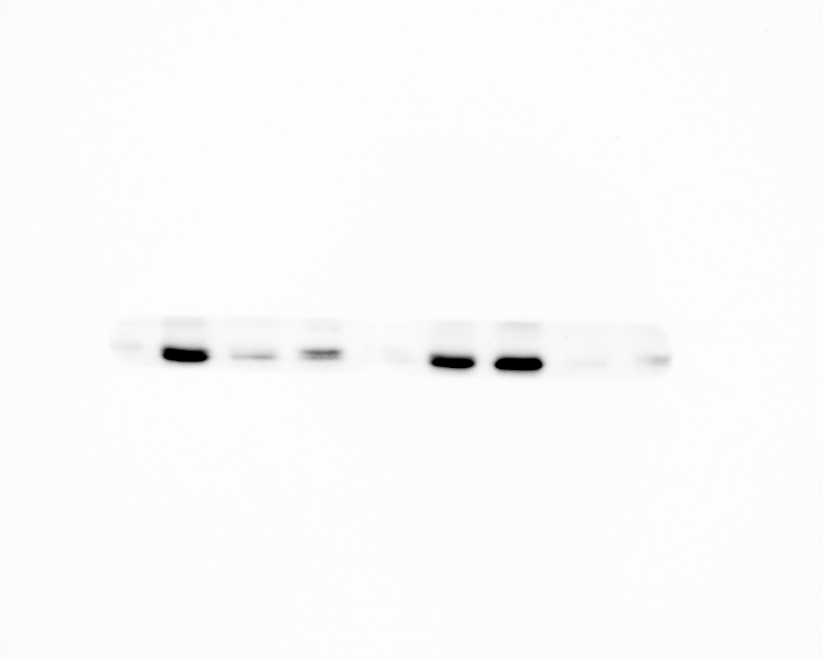

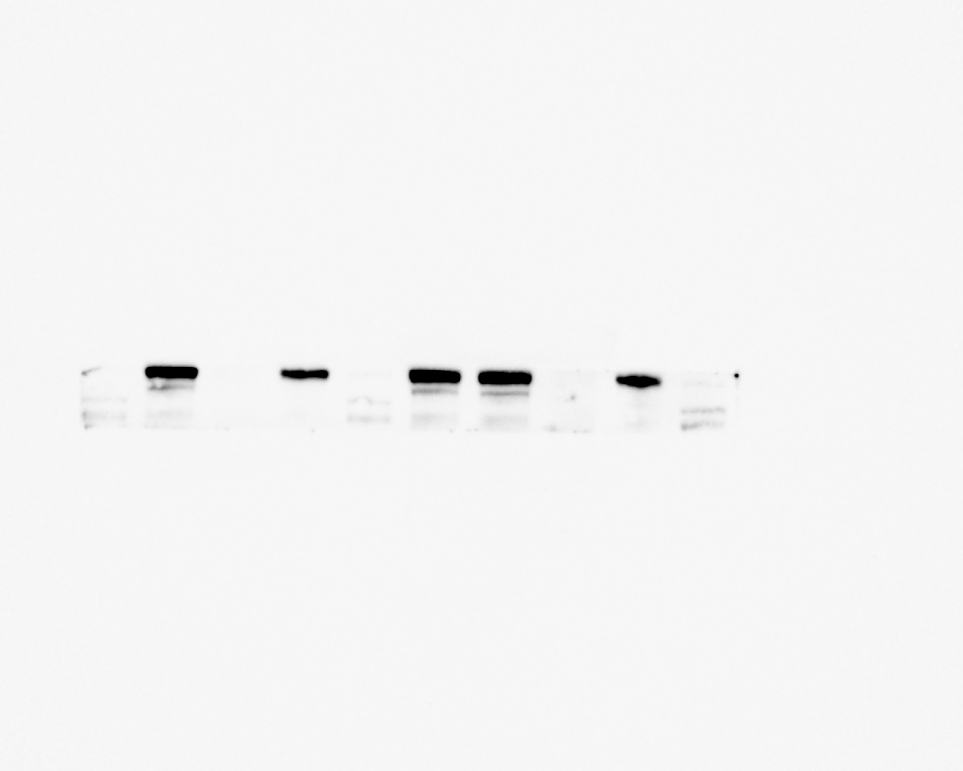

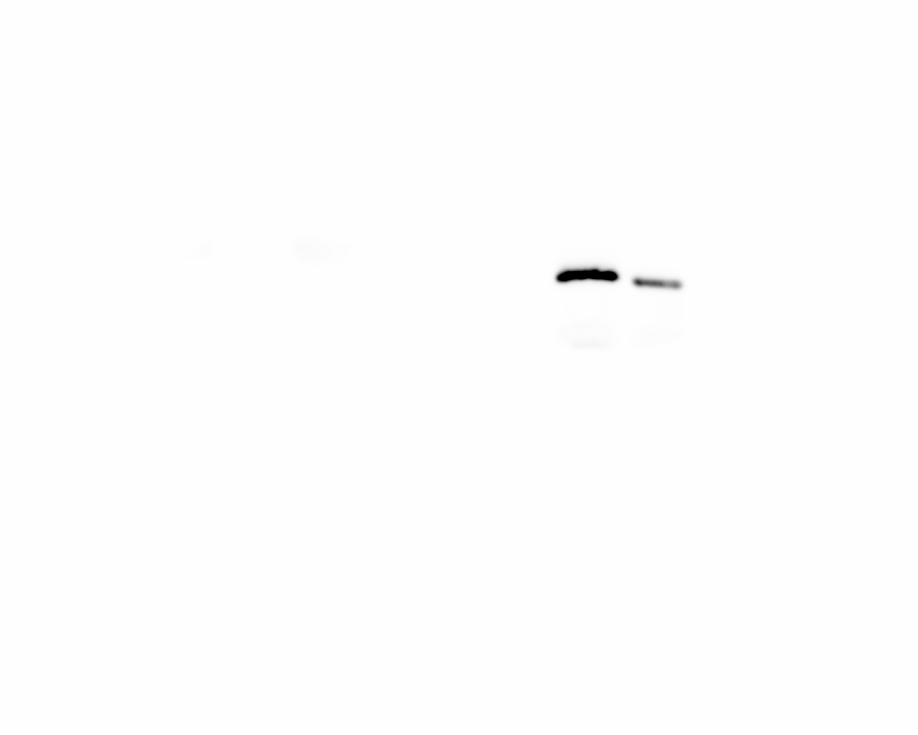

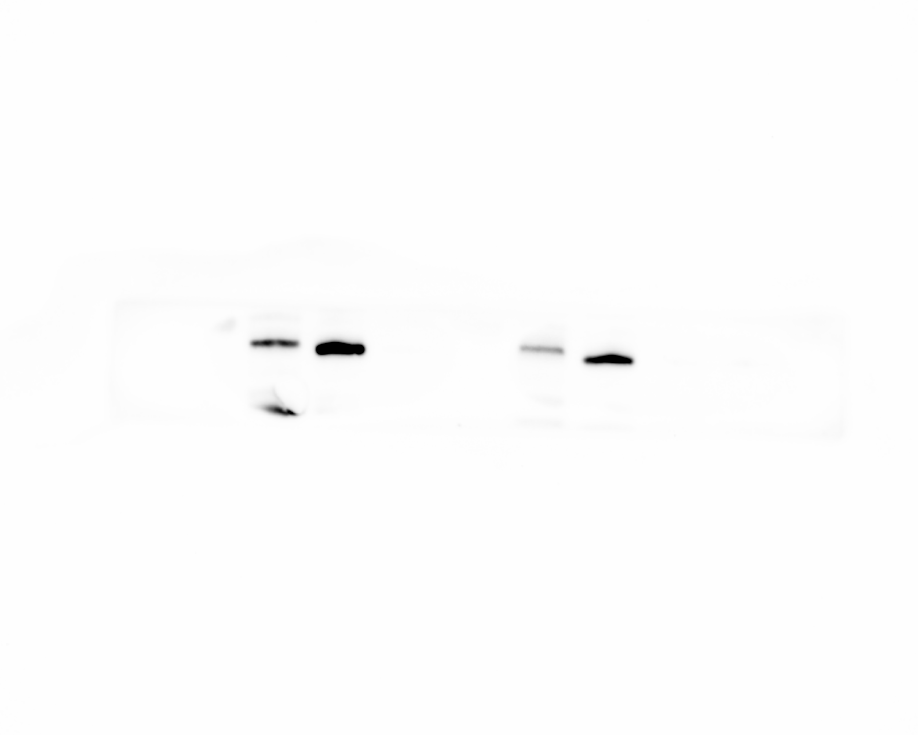

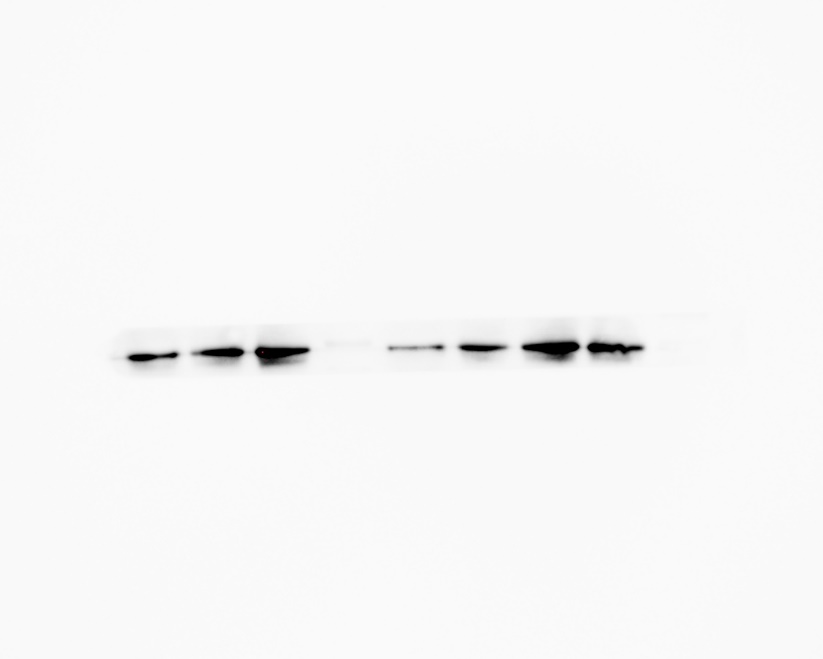

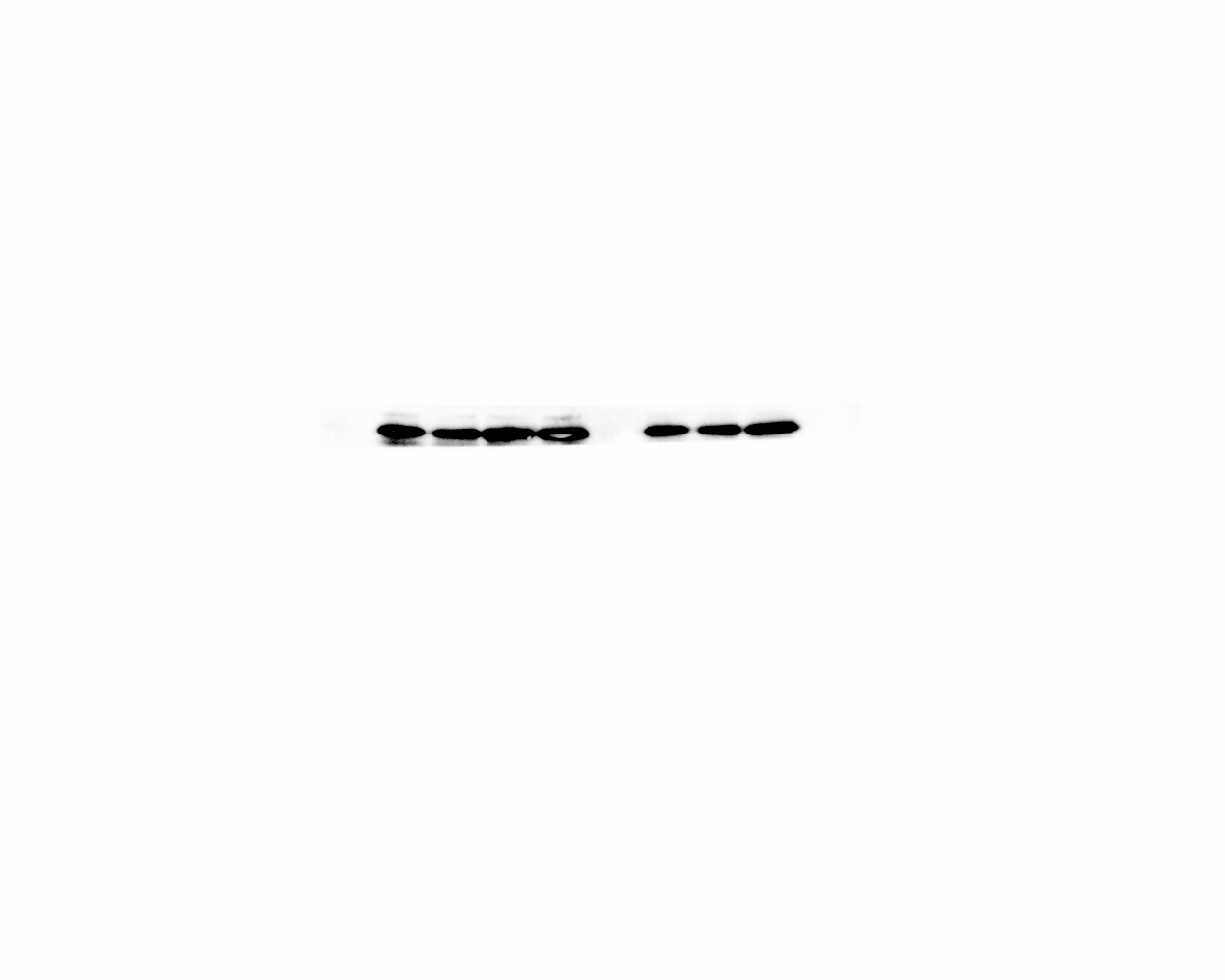

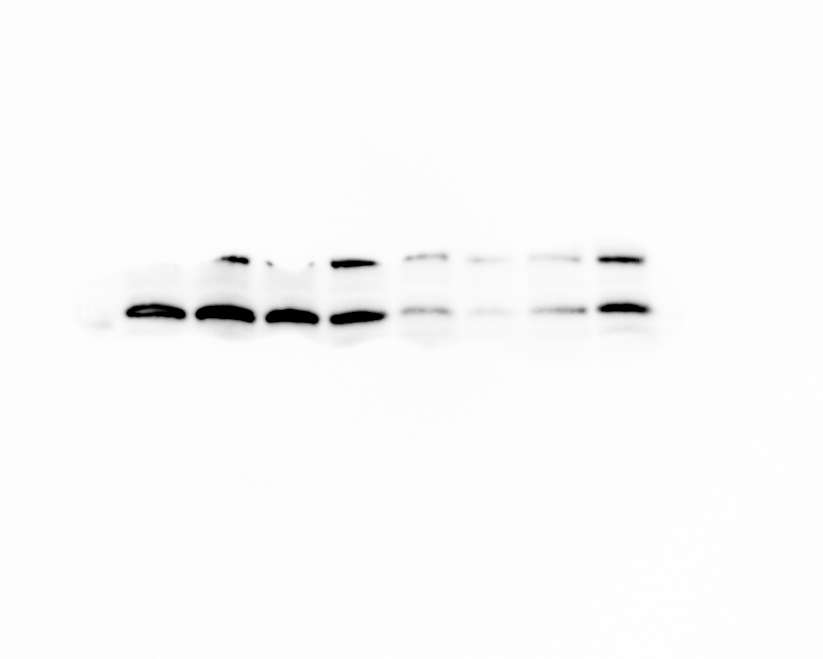

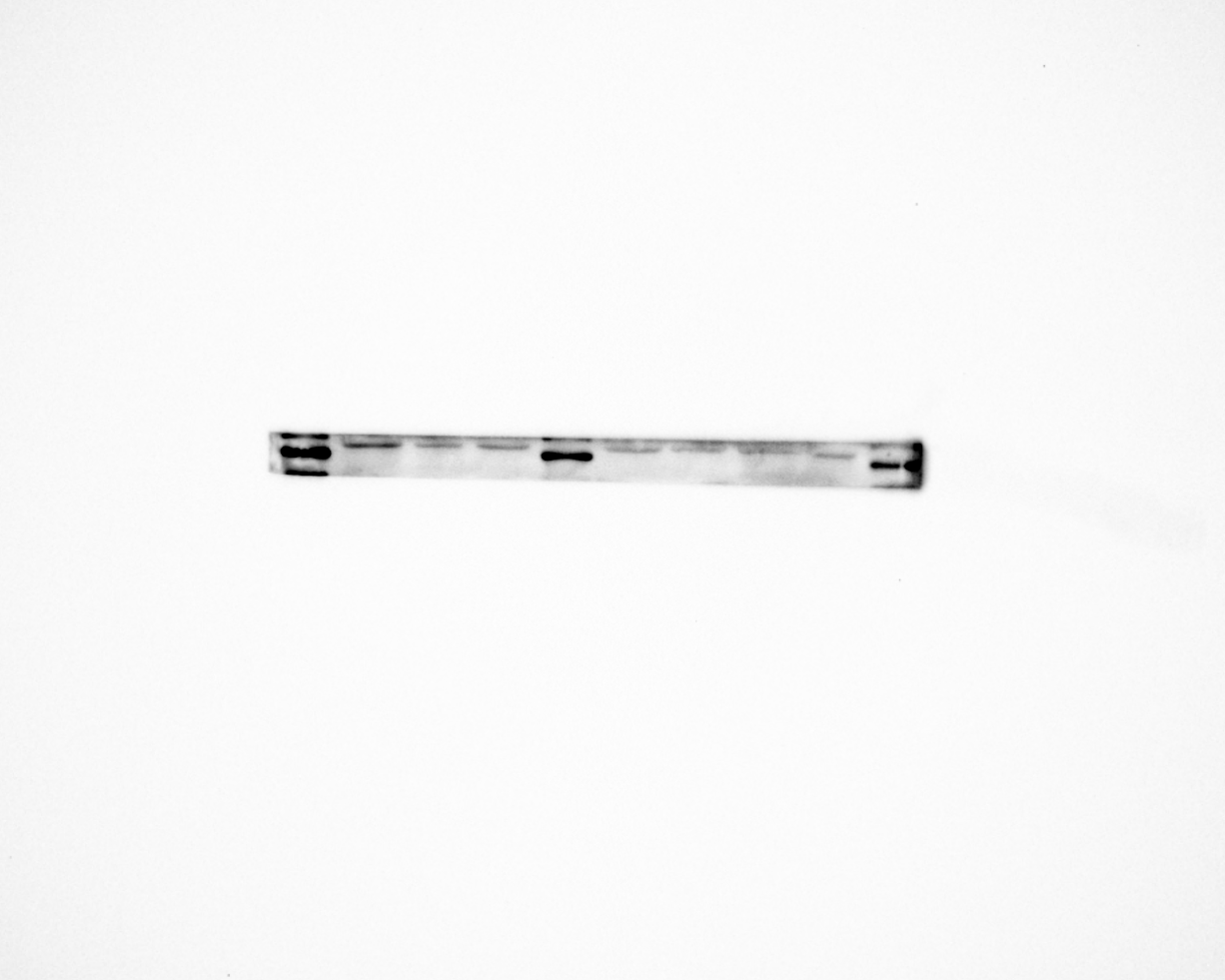

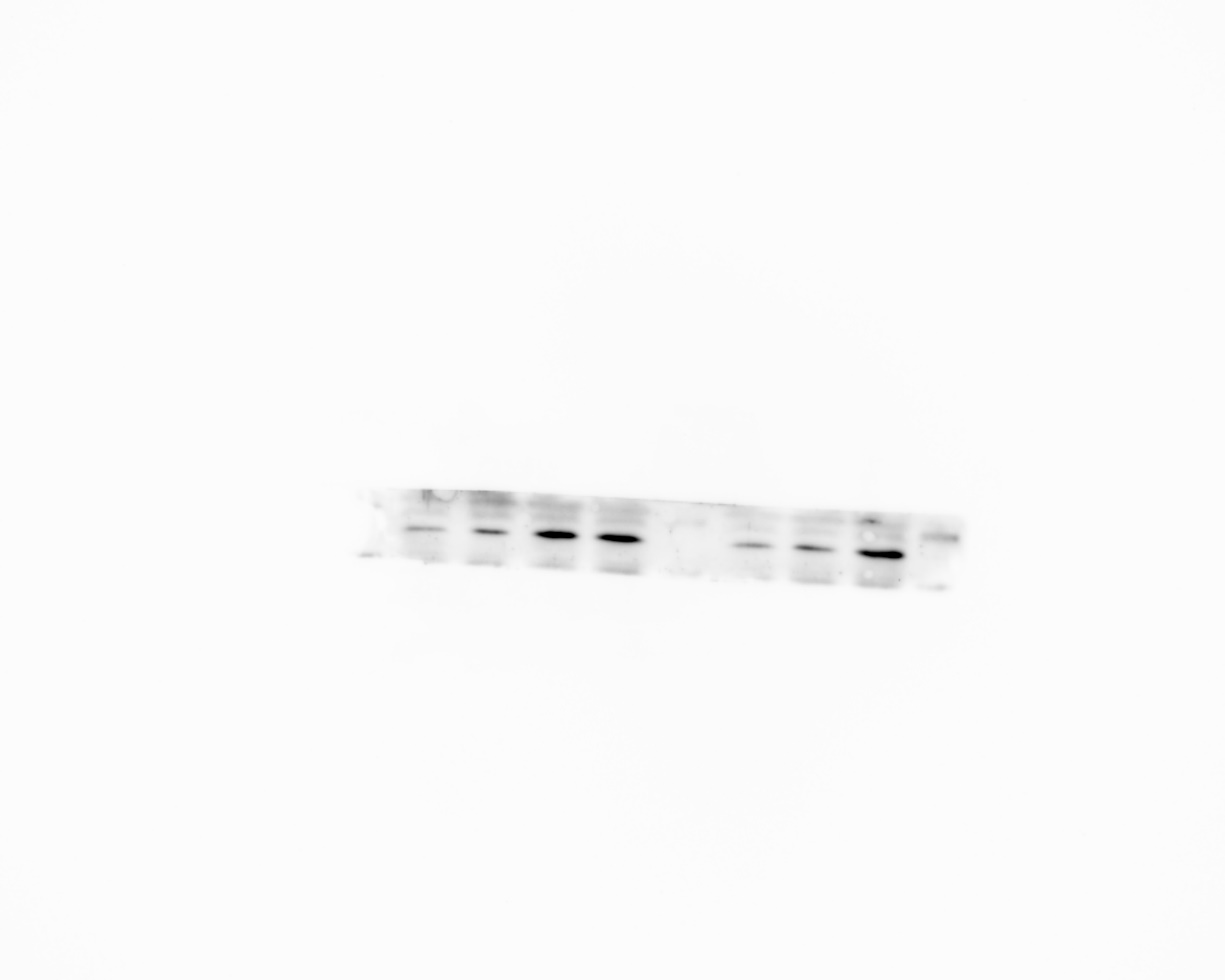

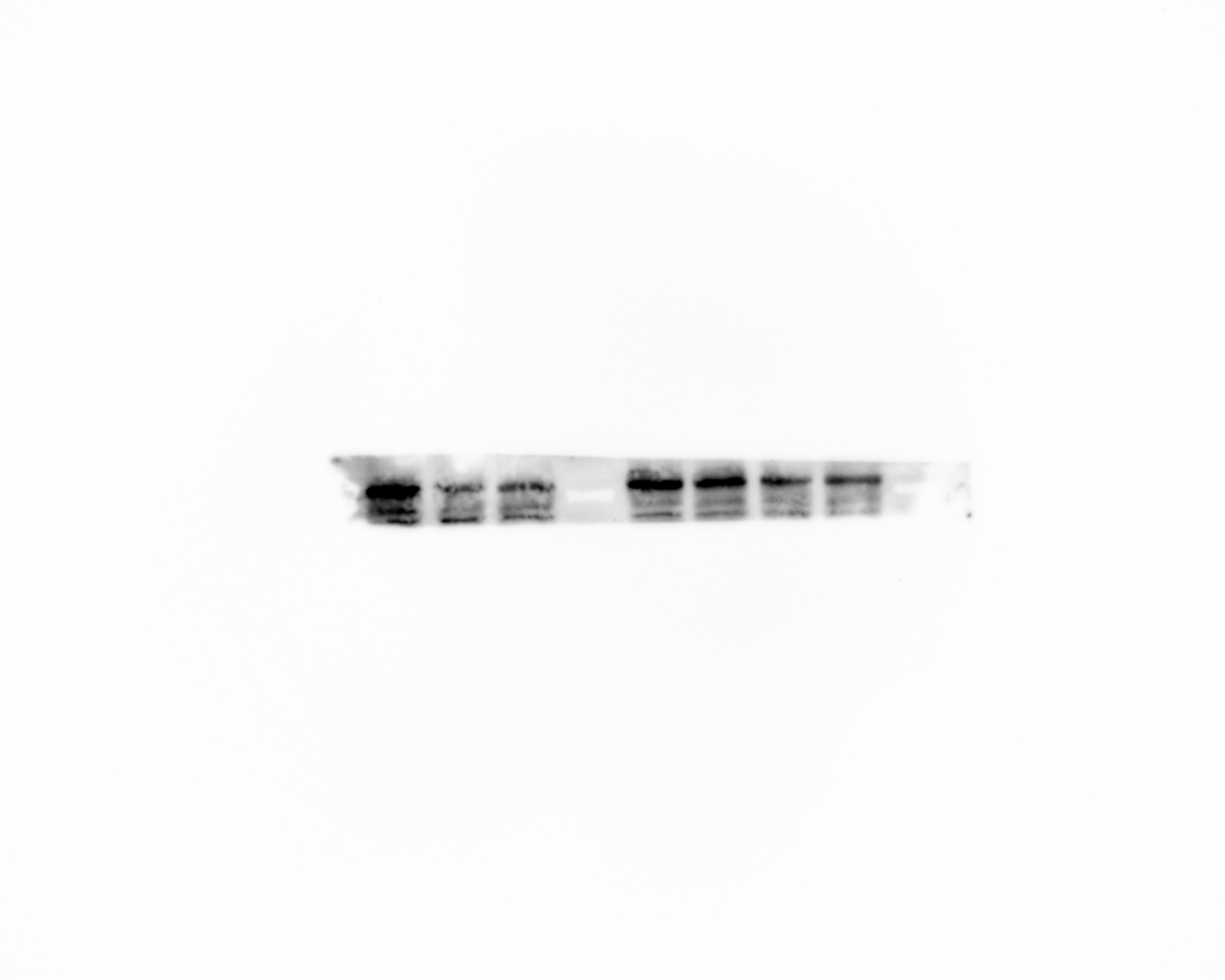

Supplement: Supplementary file 1 — Western blotting [file 41419_2022_4788_MOESM1_ESM.docx]

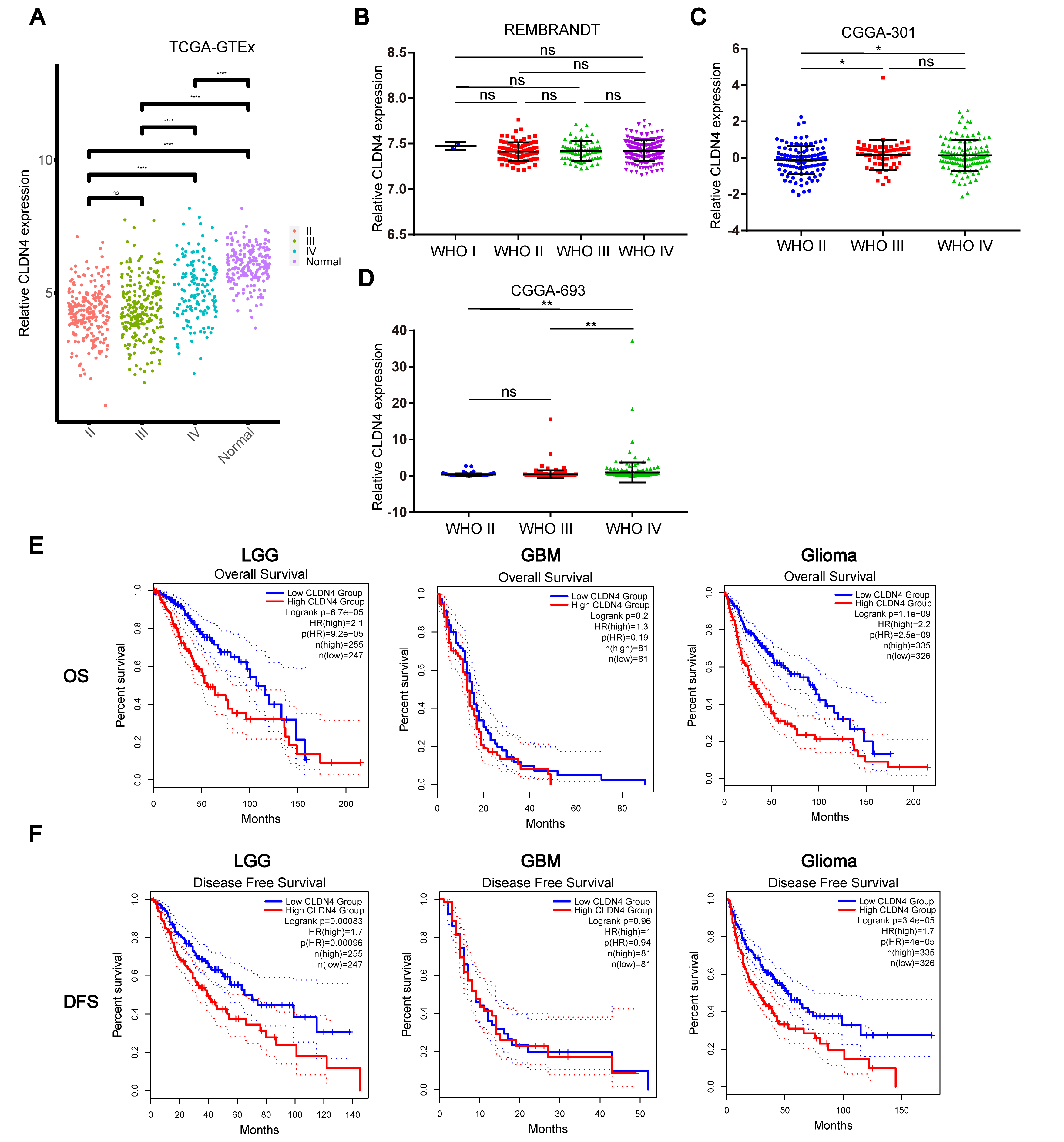

Supplement: Supplementary file 5 — Figure S1 [file 41419_2022_4788_MOESM5_ESM.tif]

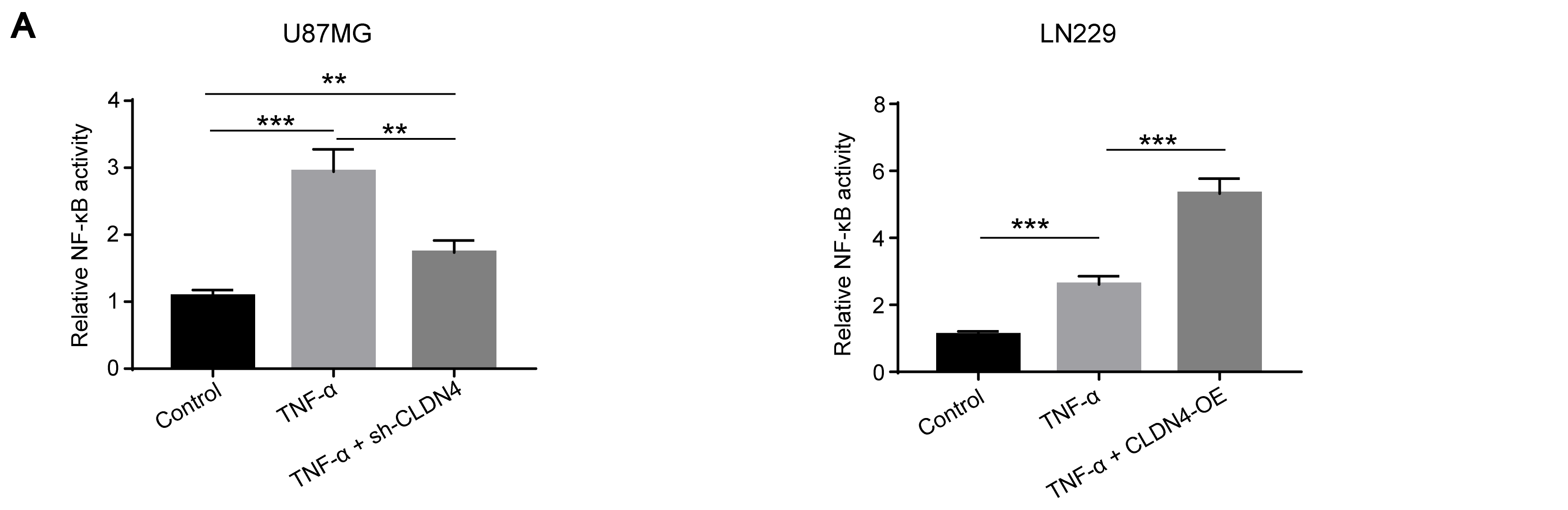

Supplement: Supplementary file 6 — Figure S2 [file 41419_2022_4788_MOESM6_ESM.tif]
