# Supplementary material for: TGF-β induces GBM mesenchymal transition through upregulation of CLDN4 and nuclear translocation to activate TNF-α/NF-κB signal pathway
Source: Cell Death Dis. 2022 Apr 13;13(4):339. doi: 10.1038/s41419-022-04788-8 (PMC9008023; doi:10.1038/s41419-022-04788-8)
Supplement: Supplementary file 3 — Supplementary FIGURE LEGENDS [file 41419_2022_4788_MOESM3_ESM.docx]

**Appendix A. Supplementary FIGURE LEGENDS**

**Figure S1. The prognostic values of CLDN4 in validated cohorts.** (A) The expression of CLDN4 in glioma tissues in TCGA and GTEx datasets. (B) The expression of CLDN4 in glioma tissues in REMBRANDT datasets. (C) The expression of CLDN4 in glioma tissues in CGGA-301 datasets. (D) The expression of CLDN4 in glioma tissues in CGGA-693 datasets. (E-F) The Kaplan-Meier survival curves were performed with TCGA data sets. The correlation between CLDN4 expression and overall survival (OS) and disease-free survival (DFS) in all glioma patients, Low grade glioma patients or GBM patients. **P* < 0.05, ***P* < 0.01, ****P* < 0.001.

**Figure S2. CLDN4 knockdown can inhibit the activation of NF-κB signal induced by TNF-α** (A) NF-κB reporter assays show up-regulation of NF-κB activity with the expression of CLDN4 in vitro. ***P* < 0.01, ****P* < 0.001.
