# Supplementary material for: TGF-β induces GBM mesenchymal transition through upregulation of CLDN4 and nuclear translocation to activate TNF-α/NF-κB signal pathway
Source: Cell Death Dis. 2022 Apr 13;13(4):339. doi: 10.1038/s41419-022-04788-8 (PMC9008023; doi:10.1038/s41419-022-04788-8)
Supplement: Supplementary file 7 — Supplemental Table [file 41419_2022_4788_MOESM7_ESM.docx]

**Supplementary materials-CDDIS-21-3200**

**Supplemental Table S1** Primers used in this study

| **Primer ID** | **Forward primer (5’-3’)** | **Reverse primer (5’-3’)** |
| --- | --- | --- |
| CLDN4 | AGAGTGGATGGACGGGTTT | GAAGGGGCAGAGGACTCA |

**Supplemental Table S2** Antibodies used in this study

| **Antibodies** | **Source** | **Category No.** |
| --- | --- | --- |
| Anti-GAPDH antibody | Cell Signaling Technology | 5174 |
| Phospho-IKKα/β (Ser176/180) Antibody Sampler Kit | Cell Signaling Technology | 9958T |
| Anti-CLDN4 antibody | Proteintech | 16195-1-AP |
| Anti-E-Cadherin antibody | Proteintech | 20874-1-AP |
| Anti-N-Cadherin antibody | Proteintech | 22018-1-AP |
| Anti-Vimentin antibody | Proteintech | 10366-1-AP |
| Anti-IKKβ antibody | Proteintech | 15649-1-AP |
| Anti-NF-ΚB P65 antibody | Proteintech | 66535-1-Ig |
| Phospho-NF-κB p65 (Ser536) (93H1) Rabbit mAb | Cell Signaling Technology | 3033 |
| Anti-IkB Alpha antibody | Proteintech | 10268-1-AP |
| Anti-SMAD2 antibody | Proteintech | 12570-1-AP |
| Phospho-SMAD2 (Ser465/Ser467) (E8F3R) Rabbit mAb | Cell Signaling Technology | 18338 |
| Anti-Lamin A/C antibody | Proteintech | 10298-1-AP |
| β-actin | Cell Signaling Technology | 8457T |
